# Immune checkpoint inhibitor-induced colitis is mediated by polyfunctional lymphocytes and is dependent on an IL23/IFNγ axis

**DOI:** 10.1038/s41467-023-41798-2

**Published:** 2023-10-23

**Authors:** Jonathan W. Lo, Domenico Cozzetto, James L. Alexander, Nathan P. Danckert, Matthew Madgwick, Naomi Knox, Jillian Yong Xin Sieh, Marton Olbei, Zhigang Liu, Hajir Ibraheim, Jesus Miguens Blanco, Hiromi Kudo, Rocio Castro Seoane, Lucia A. Possamai, Robert Goldin, Julian Marchesi, Tamas Korcsmaros, Graham M. Lord, Nick Powell

**Affiliations:** 1 Division of Digestive Diseases, Faculty of Medicine, Imperial College London, London, W12 0NN UK; 2 Organisms and Ecosystems, Earlham Institute, NR4 7UZ Norwich, UK; 3 Gut Microbes and Health Programme, Quadram Institute Bioscience, NR4 7UQ Norwich, UK; 4 School of Immunology and Microbial Sciences, King’s College London, London, SE1 9RT UK; 5 Faculty of Biology, Medicine and Health, University of Manchester, Manchester, M13 9NT UK

**Keywords:** Inflammatory bowel disease, Immunotherapy, Mucosal immunology, Computational biology and bioinformatics

## Abstract

Immune checkpoint inhibitors (CPIs) are a relatively newly licenced cancer treatment, which make a once previously untreatable disease now amenable to a potential cure. Combination regimens of anti-CTLA4 and anti-PD-1 show enhanced efficacy but are prone to off-target immune-mediated tissue injury, particularly at the barrier surfaces. To probe the impact of immune checkpoints on intestinal homoeostasis, mice are challenged with anti-CTLA4 and anti-PD-1 immunotherapy and manipulation of the intestinal microbiota. The immune profile of the colon of these mice with CPI-colitis is analysed using bulk RNA sequencing, single-cell RNA sequencing and flow cytometry. CPI-colitis in mice is dependent on the composition of the intestinal microbiota and by the induction of lymphocytes expressing interferon-γ (IFNγ), cytotoxicity molecules and other pro-inflammatory cytokines/chemokines. This pre-clinical model of CPI-colitis could be attenuated following blockade of the IL23/IFNγ axis. Therapeutic targeting of IFNγ-producing lymphocytes or regulatory networks, may hold the key to reversing CPI-colitis.

## Abstract (web summary)

Immune checkpoint inhibitors (CPI) could effectively target cancers that are resistant to traditional therapy but may initiate immune related adverse effects, such as colitis. Here, authors characterise the gut immune microenvironment during CPI-colitis by bulk RNA sequencing, single-cell RNA sequencing and flow cytometry, and find that interleukin 23 plays an important role in promoting inflammation via cytotoxic polyfunctional IFNγ-producing lymphocytes.

## Introduction

Immune checkpoint inhibitors (CPIs) are highly effective immunotherapeutics that have transformed treatment paradigms for several cancers^1–6^. They block inhibitory immune checkpoint molecules, such as CTLA4 and PD-1, restoring immune activation and bolstering anti-tumour immunity. Simultaneous administration of anti-CTLA4 and anti-PD-1 combination therapies is efficacious in advanced melanoma and renal cell carcinoma^6–9^. Unfortunately, CPIs, especially in combination, also trigger off-target immune activation in non-cancer tissues causing immune-mediated organ injury, causing significant morbidity and mortality^10–12^. Diarrhoea/colitis is arguably the most important, affecting as many as 50% of CPI-treated patients and is the most common cause of serious, life-threatening complications, treatment interruption and permanent discontinuation of CPI therapy^10–12^. CPI-induced colitis is treated with high-dose steroids, often for prolonged periods. Unfortunately, 40% of patients fail to respond to steroids and others develop severe complications of immunosuppression, including life-threatening infection^13,14^. There are important concerns that immunosuppression might impede the anti-cancer responses of CPI therapy. Two independent studies in different tumour settings, identified impaired survival in steroid exposed patients^15,16^. Treatment outcomes with biological therapies, such as anti-TNF monoclonal antibodies (mAbs) are also heterogeneous, especially if robust outcome measures are used. Not only do many patients fail to achieve steroid-free remission, side effects, such as severe infections, frequently complicate treatment^17^.

Single-cell sequencing of colonic immune cells has identified expansion and activation of both CD4^+^ and CD8^+^ T cells in CPI colitis, including upregulation of cytolytic programmes and increased expression of interferon-γ (IFNγ)^18^. A second study focussing on CD8^+^ cells replicated these findings and identified expansion of tissue-resident populations as key cytokine-producing cells^19^. There is now a pressing need for mechanistic insights to understand the critical immune drivers and regulators of CPI colitis to inform the development of targeted therapies. Here, we present a mouse model of CPI colitis which mimics the human disease in multiple manners both at protein and transcriptional levels. This preclinical model of CPI colitis is shown to be dependent on the composition of the microbiota driven by cytotoxic polyfunctional lymphocytes and reliant on the IL23/IFNγ axis.

## Results

### The composition of the intestinal microbiota regulates susceptibility to immune checkpoint inhibitor-induced colitis

**Fig. 1:**
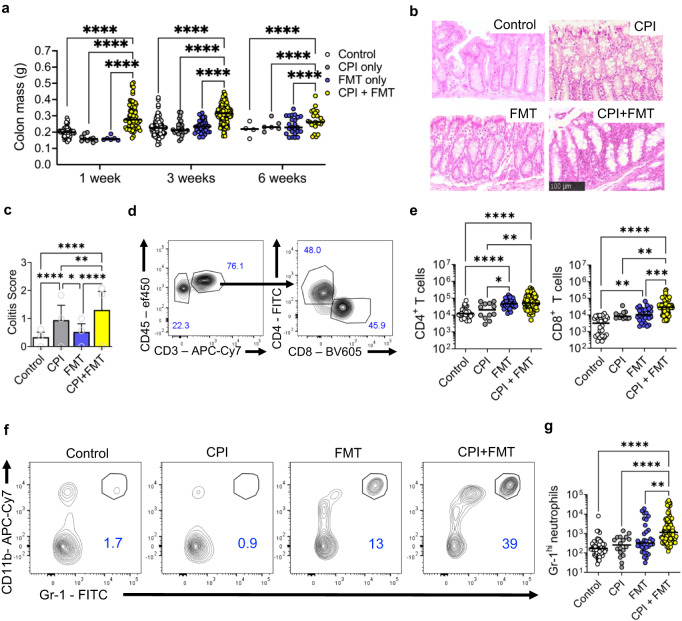
Intestinal microbiota regulates susceptibility to CPI-induced colitis. **a** Colon mass in wildtype female 6-week-old Balb/C mice at 1 week, 3 weeks and 6 weeks without treatment (Control: *n* = 56 at 1 week, *n* = 79 at 3 weeks, *n* = 4 at 6 weeks), treatment with combination anti-CTLA4/anti-PD-1 (CPI: *n* = 10 at 1 week, *n* = 25 at 3 weeks, *n* = 6 at 6 weeks), treatment with fecal microbiota (FMT: *n* = 6 at 1 week, *n* = 35 at 3 weeks, *n* = 24 at 6 weeks) and mice treated with both CPI and FMT (CPI + FMT: *n* = 68 at 1 week, *n* = 182 at 3 weeks, *n* = 23 at 6 weeks). Two-way ANOVA with Tukey’s multiple comparison test used ****P < 0.0001. CPI injections were given once a week intraperitoneally. **b** Representative colon sections cut at 3 µm thick FFPE from control, CPI, FMT and CPI + FMT treated wildtype female 6-week-old Balb/C mice stained with H&E (Leica). **c** Summary histological colitis score from mice treated with CPI-C (*n* = 11), plus controls (Control: *n* = 6, CPI: *n* = 7 and FMT: *n* = 7). Average colitis scored based on apoptosis (0–3), infiltrating lymphocytes (0–3), crypt abscess formation (0–3). Bar graph showing mean with SEM and Two-way ANOVA with Tukey’s multiple comparison test used whereby *P = 0.0106 **P = 0.0051 and ****P < 0.0001. **d** Representative flow cytometry plot showing the CD4^+^ and CD8^+^ T cell gating. **e** Number of CD4^+^ and CD8^+^ T cells from the colonic lamina propria of Control (*n* = 29), CPI (*n* = 12), FMT (*n* = 30) and CPI + FMT (*n* = 79) treated wildtype female 6-week-old Balb/C mice. Two-way ANOVA with Tukey’s multiple comparison test used ****P < 0.0001. **f** Representative flow cytometry plot showing Gr-1^hi^ neutrophils from Control, CPI, FMT and CPI + FMT treated wildtype female 6-week-old Balb/C mice. **g** Number of Gr-1^hi^ neutrophils from the colonic lamina propria of Control (*n* = 70), CPI (*n* = 18), FMT (*n* = 33) and CPI + FMT (*n* = 95) treated wildtype female 6-week-old Balb/C mice. Two-way ANOVA with Tukey’s multiple comparison test used **P<0.01 ****P < 0.0001.

To understand how immune checkpoint perturbation impacts the colonic immune system, we administered anti-CTLA4/anti-PD-1 combination therapy to wild-type (WT) Balb/C mice. Combination checkpoint inhibition triggered systemic immune activation with induction of splenomegaly, even after 1 week of treatment, however, there was no change in colonic mass, and only mild histological changes observed (Fig. 1a–c and Supplementary Fig. 1a–c). In CPI-treated cancer patients, the intestinal microbiome influences colitis susceptibility^20–23^, therefore, we investigated whether altering the intestinal microbiota impacted colitis susceptibility. To test this hypothesis, we transplanted the intestinal microbiota harvested from TRUC mice^24,25^, that are colonised with a pro-inflammatory microbiota to WT mice. Although fecal microbiota transplantation (FMT) alone did not induce colitis, subsequent challenge with combination anti-CTLA4/anti-PD-1 therapy triggered increased colon mass and more marked histological changes (Fig. 1a–c and Supplementary Fig. 1a–c). These changes were apparent after 1 week of treatment and maintained for up to 6 weeks (Fig. 1a and Supplementary Fig. 1a, b). Weight gain was also significantly diminished in mice receiving combination anti-CTLA4/anti-PD-1 and FMT in comparison with control mice (Supplementary Fig. 1b).

In mice treated with combination CPI, the overall histological changes in the colon at 3 weeks were still relatively mild but showed features consistent CPI colitis in patients, showing increased lymphocytes in the colonic lamina propria (cLP), increased intraepithelial lymphocytes, and increased epithelial apoptosis (Fig. 1b, c and Supplementary Fig. 1c). Colonic lymphocyte expansion was corroborated by flow cytometry, which showed expansion of CD4^+^ and CD8^+^ T cells in the cLP and intraepithelial compartment following treatment with combination anti-CTLA4/anti-PD-1 and FMT (Fig. 1d, e and Supplementary Fig. 1d–f).

Colonic neutrophil accumulation is a key feature of CPI colitis^26^. There was significant accumulation of CD11b^+^ Gr-1^high^ neutrophils, in the colon of mice treated with FMT and CPI, although there were no changes in the number of infiltrating Siglec F^+^ eosinophils (Fig. 1f–g and Supplementary Fig. 1g–i). Disease could also be induced in C57BL/6 mice with combination CPI and FMT (Supplementary Fig. 2a), indicating that this phenomenon occurs across different mouse strains.

We assessed whether anti-CTLA4 or anti-PD-1 was likely driving these pathological changes. Key clinical features, colitis scores and accumulation of CD11b^+^ Gr-1^high^ neutrophils were more severe in recipients of anti-CTLA4 and anti-PD-1 combination treatment than single agent treatment, and changes in anti-PD-1 recipients were generally milder than in anti-CTLA4 monotherapy (Supplementary Fig. 3a–d). Accumulation of CD4^+^ and CD8^+^ T cells in both the lamina propria and intraepithelial compartment of the colon were most pronounced in mice receiving combination treatment, although could also be observed in both monotherapies, especially in the intraepithelial layer (Supplementary Fig. 3e, f). Overall, these data are consistent with observations in human disease, where combination therapy causes more penetrant and severe colitis^8,27^.

### Transplantation of colitis-permissive microbiota results in durable shifts in the fecal microbiota community composition

**Fig. 2:**
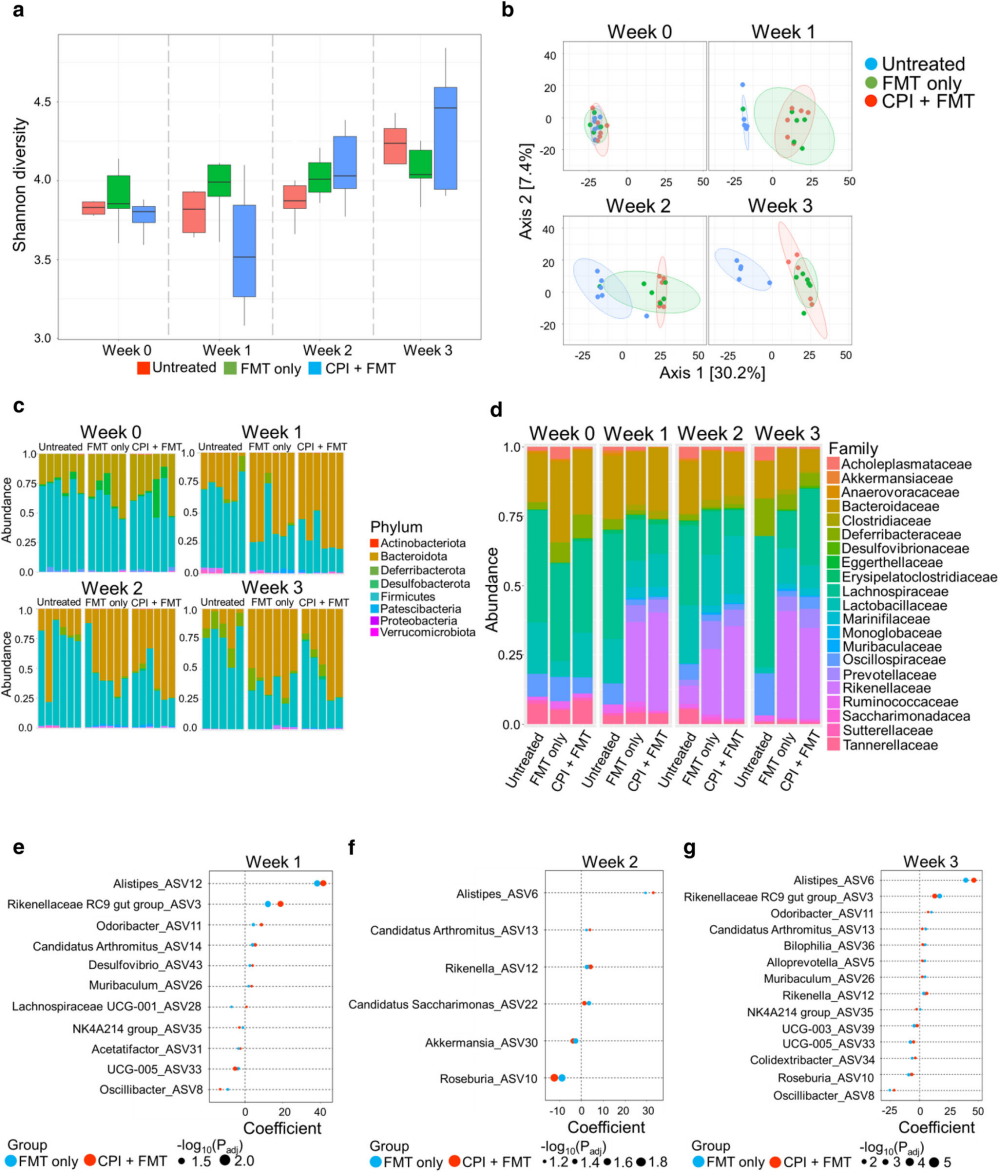
Longitudinal 16S analysis of microbiota shows early and persistent changes in composition of microbiota after FMT. **a** Alpha-diversity (Shannon diversity index) of the microbiota between untreated control Balb/C wildtype female 6-week-old mice (*n* = 6), Balb/C wildtype mice treated with FMT (*n* = 6) and Balb/C wildtype female 6-week-old mice treated with CPI + FMT (*n* = 6) longitudinally over a 3 week period. A liner mixed model was used, based on ANCOM-BC, to account for the repeated measures of the mice. FDR Bonferroni corrected Padj. Box plot is defined by: Week 0 – Untreated = “Min.:3.775” “1st Qu.:3.787” “Median :3.828” “Mean:3.856” “3rd Qu.:3.867” “Max.:4.051” Week 1 – Untreated = “Min. :3.640” “1st Qu.:3.670” “Median :3.819” “Mean :3.909” “3rd Qu.:3.929” “Max. :4.586” Week 2 – Untreated =“Min. :3.659” “1st Qu.:3.823” “Median :3.871” “Mean :3.911” “3rd Qu.:3.968” “Max. :4.255“Week 3 – Untreated =“Min. :3.148” “1st Qu.:4.105” “Median :4.236” “Mean :4.049” “3rd Qu.:4.331” “Max. :4.427” Week 0 – FMT-only = “Min.:3.603” “1st Qu.:3.823” “Median:3.852” “Mean :3.890” “3rd Qu.:4.033” “Max. :4.137” Week 1 – FMT-only = “Min. :3.610” “1st Qu.:3.901” “Median :3.990” “Mean :4.007” “3rd Qu.:4.100” “Max. :4.445” Week 2 – FMT-only = “Min. :3.859” “1st Qu.:3.927” “Median :4.008” “Mean :4.021” “3rd Qu.:4.114” “Max. :4.206” Week 3 – FMT-only = “Min.:3.832” “1st Qu.:4.016” “Median :4.037” “Mean :4.069” “3rd Qu.:4.193” “Max. :4.250” Week 0 – CPI = “Min. :3.591” “1st Qu.:3.734” “Median :3.801” “Mean :3.772” “3rd Qu.:3.836” “Max. :3.878” Week 1 – CPI= “Min. :3.080” “1st Qu.:3.265” “Median :3.515” “Mean :3.557” “3rd Qu.:3.844” “Max. :4.097” Week 2 – CPI= “Min. :3.770” “1st Qu.:3.952” “Median :4.030” “Mean :4.084” “3rd Qu.:4.280” “Max. :4.384” Week 3 – CPI = “Min. :3.902” “1st Qu.:3.944” “Median :4.461” “Mean :4.348” “3rd Qu.:4.592” “Max. :4.839” **b** Non-metric dimensional scaling plot showing the beta diversity of the microbiota from untreated control Balb/C wildtype female 6-week-old mice (*n* = 6), Balb/C wildtype female 6-week-old mice treated with FMT (*n* = 6) and Balb/C wildtype female 6-week-old mice treated with CPI + FMT (*n* = 6) longitudinally over a 3 week period. **c** Phylum level relative abundance profiles from untreated control Balb/C wildtype female 6-week-old mice (*n* = 6), Balb/C wildtype female 6-week-old mice treated with FMT (*n* = 6) and Balb/C wildtype female 6-week-old mice treated with CPI + FMT (*n* = 6) longitudinally over a 3 week period. **d** Family level relative abundance profiles from untreated control Balb/C wildtype female 6-week-old mice (*n* = 6), Balb/C wildtype female 6-week-old mice treated with FMT (*n* = 6) and Balb/C wildtype female 6-week-old mice treated with CPI + FMT (*n* = 6) longitudinally over a 3 week period. Genus level coefficient changes comparing Balb/C wildtype female 6-week-old mice treated with FMT (*n* = 6) and Balb/C wildtype female 6-week-old mice treated with CPI + FMT (*n* = 6) at **e** 1 week, **f** 2 weeks and **g** 3 weeks of treatment. A liner mixed model was used, based on ANCOM-BC, to account for the repeated measures of the mice. FDR Bonferroni corrected *P*adj.

Longitudinal changes in the intestinal microbiome following treatment with FMT alone or FMT with combination CPI therapy were analysed (Supplementary Fig. 4a). Using 16S rRNA amplicon sequencing, compared to baseline, there were no significant changes in α-diversity following FMT alone (Shannon index mean 4.00 & *P* = 0.56) or FMT with combination CPI (Shannon index mean 3.92 & *P* = 0.56) throughout the duration of the experiment (Fig. 2a and Supplementary Fig. 4b); however, after 1 week β-diversity was significantly different between control and FMT groups (R2 = 0.283; *P* = 0.004, Fig. 2B) and between control and FMT with combination CPI groups (R2 = 0.379; *P* = 0.003, Fig. 2b), indicating a profound shift in microbiota community structure. These differences were durable throughout the 28-day course of the experiment (Fig. 2b), indicating persistent engraftment of the transplanted microbiota. No difference was seen between the FMT alone and the FMT with combination CPI groups (R2 = 0.118; *P* = 0.216, Fig. 2b), indicating that FMT was the primary driver of the shift in community structure. At phylum level, there was a shift in composition after 1 week in both the FMT alone and FMT with combination CPI groups towards increased *Bacteroidota* and reduced *Firmicutes* (Fig. 2c). This shift in the *Bacteroidota:Firmicutes* ratio persisted to the end of the experiment. At family level, an immediate and persistent increase in *Rikenellaceae and Prevotellaceae* was observed in both the FMT alone and FMT with combination CPI groups, with a concomitant reduction in *Tannerellaceae* and *Oscillospiraceae* (Fig. 2d). There were no changes observed in probiotic microbes, like *Bifidobacteria* and *Lactobacilli*, in these mice, especially with these having been shown to play a functional role in CPI-C^21^ (Fig. 2d and Supplementary Fig. 4c). A total of 17 genera were differentially abundant comparing between controls and FMT alone/FMT with combination CPI at week 1 after treatment (Fig. 2e). Genera more abundant in the two treatment groups included *Alistipes, Rikenella* RC9 gut group, *Odoribacter* and *Alloprevotella*. By contrast, *Lachnospiraceae NK4A136 group, Oscillibacter* and *Roseburia* were significantly reduced following both FMT alone and FMT with CPI combination therapy (Fig. 2e). In keeping with the longitudinal findings in phylum and family composition, genus level differences after 1 week were consistent at 2- and 3-weeks following treatment (Fig. 2f, g).

### Transcriptomic profiling demonstrates colonic epithelial dysfunction, interferon signalling and cytotoxicity in CPI colitis

**Fig. 3:**
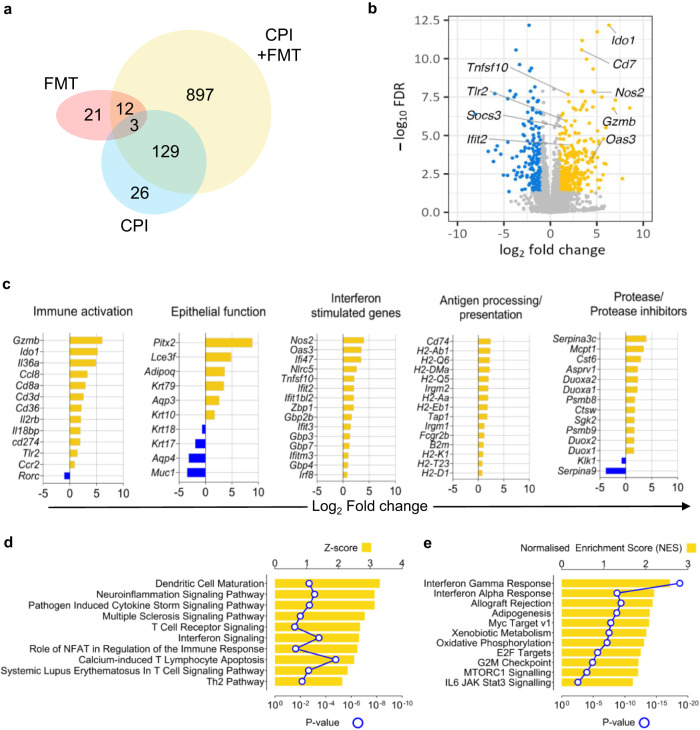
Transcriptome analysis shows colonic epithelial dysfunction and activation of T cells, cytotoxicity, and immune compartments in mice with CPI-induced colitis. **a** Venn diagram of the DEGs (FDR < 0.05) identified by comparing the expression profiles of whole colon biopsies from wildtype female 6-week-old mice with only CPI treatment (*n* = 4), only FMT (*n* = 3) or with both CPI + FMT (*n* = 3) against control wildtype female 6-week-old mice (*n* = 4). **b** Volcano plot highlighting the DEGs (FDR < 0.05) from the comparison of RNA samples from wildtype female 6-week-old mice treated with both CPI + FMT (*n* = 3) versus control wildtype female 6-week-old mice (*n* = 4). Positive log fold changes indicate over-expression in treated mice, while negative log fold changes indicate upregulation in wildtype mice. **c** The topmost significantly upregulated differentially expressed genes involved in epithelial function, protease/protease inhibitors, immune activation, interferon-stimulated genes and antigen processing/presentation in the distal colon of wildtype female 6-week-old mice with CPI-induced colitis (*n* = 3) in comparison with control wildtype female 6-week-old mice (*n* = 4). **d** Gene expression changes in the distal colon of wildtype female 6-week-old mice with CPI-induced colitis (*n* = 3) in comparison with control wildtype female 6-week-old mice (*n* = 4) were used to identify which biological pathways were significantly impacted, using IPA Canonical Pathways. *P* value adjustment for multiple comparisons was applied with the Benjamini and Hochberg method. **e** Hallmark pathway analysis using GSEA in the gene expression changes in the distal colon of wildtype female 6-week-old mice with CPI-induced colitis (*n* = 3) in comparison with control wildtype female 6-week-old mice (*n* = 4). *P* value adjustment for multiple comparisons was applied with the Benjamini and Hochberg method.

To investigate the immunopathology of this model of CPI-induced colitis, we analysed gene expression changes in the distal colon using bulk RNA sequencing. There were relatively minor transcriptional changes observed in mice treated with CPI or FMT (Fig. 3a). Following FMT, we observed significant induction (FDR < 0.05) of genes involved in humoral immunity, such as the activation-induced cytidine deaminase gene (*Aicda*), which plays a critical role in somatic hypermutation and class switching in B cells in response to microbial challenge, and over-expression of immunoglobulin chains, including selective variable regions (*Ighv7-3, Igkv1-117, Igkv15-103, Igkv9-124, Igkv8-21, Igkv4-61*) (Supplementary Fig. 5a).

Transcriptomic changes were more pronounced in mice treated with both FMT and combination CPI, with a greater number of differentially expressed genes (DEGs) and a greater magnitude of the differences, including 1041 genes significantly affected (FDR < 0.05), of which 339 showed at least doubled expression levels and 203 at least halved (Fig. 3a, b). Upregulated transcripts encoded proteins involved in immune activation, epithelial barrier function, extracellular matrix regulation and anti-microbial responses (Fig. 3c). Upregulated immune-related genes were involved in cell-mediated cytotoxicity (*Gzmb*), interferon-stimulated genes (*Nos2*, *Ido1, Oas3*, *Ifit2*, *Gbp3* and *Gbp7)*, antigen processing/presentation (*Cd74* and multiple MHC molecules) and multiple proteases and their inhibitors (*Serpina3c, Duoxa2, Cst6, Sgk2*). We also observed differential expression of genes involved in epithelial responses to tissue injury and infection at the barrier surfaces^28–30^, including keratin family genes, members of the late cornified envelope gene cluster, aquaporins and mucins (Fig. 3c). Some of these epithelial transcripts are dysregulated in the colon of patients with conventional inflammatory bowel disease (IBD)^31^.

Biological pathway analysis (Canonical Pathway Analysis, IPA, Qiagen) identified significant enrichment of biological processes, such as interferon signalling, dendritic cell maturation, T cell receptor signalling and the role of NFAT in regulation of the immune response in CPI colitis (Fig. 3d). These data were corroborated using Genome Set Enrichment Analysis (GSEA) to identify the most upregulated Hallmark pathways (Fig. 3e). Using this pathway tool, the most significantly activated pathway was the response to IFNγ.

To investigate which molecules might be responsible for regulating the transcriptional changes observed in the colon in CPI colitis, we used the Upstream Regulator Analysis tool (IPA). Molecules predicted to activate the pattern of gene expression observed in our model included cytokines (IFNγ, IL1β, IL21 and IL27), microbial products/TLR agonists (LPS, TLR3, TLR4, TLR7, TLR9, CpG and poly IC RNA), transcription factors (CEBPB and NFATC2) and signalling molecules (STAT1, STAT2, JAK1/2) (Supplementary Fig. 6a). The cytokine predicted to be most highly activated in CPI colitis was IFNγ (*z* Score= 6.028, FDR = 2.06 × 10^−23^) (Supplementary Fig. 6a).

To determine whether this model of CPI colitis mirrored aspects of human disease, we evaluated the similarity between our model and those reported in combination CPI colitis cancer patients developing colitis^32^. Consistent with our model recapitulating gene expression changes in human CPI-induced colitis, GSEA demonstrated that the mouse homologues of the most significantly upregulated genes probed in patients were among the most over-expressed ones in our model (Supplementary Fig. 7a). Similar to the situation in human disease, colitis could also be significantly attenuated by in vivo administration of anti-TNF mAbs (Supplementary Fig. 8a–c).

### Shared and distinct patterns of mucosal immune activation following CTLA4 or PD-1 blockade

**Fig. 4:**
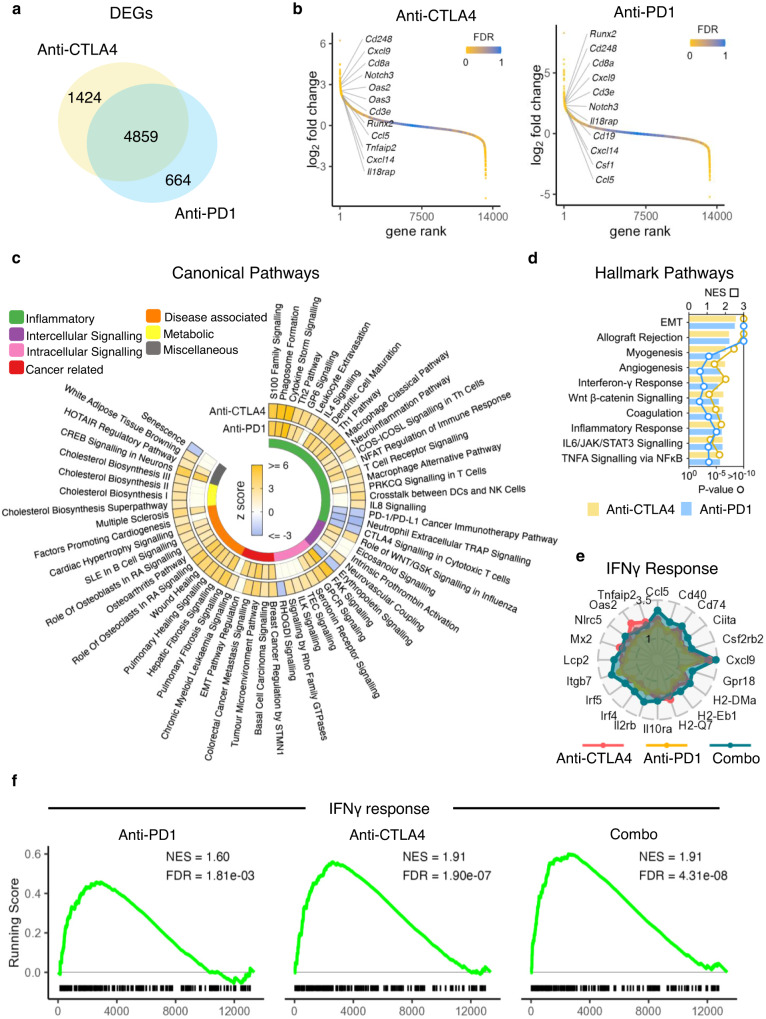
Comparison between anti-CTLA4 and anti-PD-1 monotherapy treated mice showed shared and distinct patterns of mucosal immune activation. **a** Venn diagram of the DEGs (FDR < 0.05) identified by comparing the expression profiles of whole colon biopsies from wildtype female 6-week-old mice with only anti-CTLA4 treatment (*n* = 3) or anti-PD-1 treatment (*n* = 3) in comparison to control wildtype female 6-week-old mice (*n* = 3). **b** Chair plots showing differentially expressed transcripts ranked by decreasing log fold change and coloured by estimated FDR in wildtype female 6-week-old mice with only anti-CTLA4 treatment (*n* = 3) or anti-PD-1 treatment (*n* = 3) in comparison to control wildtype female 6-week-old mice (*n* = 3). **c** Circular plot showing activation score of biological pathways which were significantly impacted, using IPA Canonical Pathways, in wildtype female 6-week-old mice with only anti-CTLA4 treatment (*n* = 3) or anti-PD-1 treatment (*n* = 3) in comparison to control wildtype female 6-week-old mice (*n* = 3). **d** Hallmark pathway analysis using GSEA in the gene expression changes in the distal colon of wildtype female 6-week-old mice with only anti-CTLA4 treatment (*n* = 3) or anti-PD-1 treatment (*n* = 3) in comparison to control wildtype female 6-week-old mice (*n* = 3). *P* value adjustment for multiple comparisons was applied with the Benjamini and Hochberg method. **e** Radar plot showing log2 fold change for the top 20 genes associated to the IFNγ responsive pathway in the gene expression changes in the distal colon of wildtype female 6-week-old mice with only anti-CTLA4 treatment (*n* = 3), anti-PD-1 treatment (*n* = 3) or combination treatment (*n* = 3), in comparison to control wildtype female 6-week-old mice (*n* = 3). **f** GSEA running score for the IFNγ responsive pathway in the gene expression changes in the distal colon of wildtype female 6-week-old mice with only anti-CTLA4 treatment (*n* = 3), anti-PD-1 treatment (*n* = 3) or combination treatment (*n* = 3), in comparison to control wildtype female 6-week-old mice (*n* = 3).

Next, we asked whether anti-CTLA4 and anti-PD-1 treatment regulated different biological processes in the colonic microenvironment. Molecular profiling of the colon following induction of colitis with either anti-CTLA4 or anti-PD-1 monotherapy was performed using RNA-seq. Although the majority of significantly DEGs were common to both anti-CTLA4 or anti-PD-1 treatment, anti-CTLA4 treatment induced more than twice as many unique DEGs as compared to anti-PD-1 treatment (1424 vs 664 transcripts) (Fig. 4a, b). Consistent with anti-CTLA4 having a more dominant effect, for the 4859 genes significantly impacted by both anti-PD-1 and anti-CTLA4 treatment, the latter induced larger expression changes on 3159 of them (65%) (median difference in absolute fold change equal to 1.05, *P* < 2.2e-16 from paired Wilcoxon signed rank sum test).

A similar pattern was observed at pathway level, with both anti-CTLA4 and anti-PD-1 treatment activating common biological pathways. Canonical biological pathways (Ingenuity) simultaneously engaged by both treatments included inflammatory pathways, such as activation of T cells (TCR signalling, T_H_1 pathway and T_H_2 pathway) and myeloid cells (dendritic cell maturation and macrophage classical pathway), and pathways associated with other diseases, such as other autoimmune diseases (systemic lupus erythematosus (SLE) and multiple sclerosis) and fibrotic diseases (pulmonary fibrosis, hepatic fibrosis and wound healing) (Fig. 4c). Anti-CTLA4 was associated with significant enrichment of 17 unique pathways, including activation of inflammatory pathways (IL8 signalling, DC/NK cross-talk and leucocyte extravasation), signalling pathways (Tec, ILK and Rho family GTPase signalling) and immunometabolism, most notably concerning cholesterol biosynthesis (Fig. 4c). There were fewer pathways uniquely modulated by anti-PD-1 treatment, and these pathways included activation of NFAT signalling and eicosanoid signalling (Fig. 4c).

Pathway analysis using GSEA Hallmark also highlighted shared patterns of regulation of pro-inflammatory by both anti-CTLA4 and anti-PD-1. For example, both treatments upregulated pathways driven by pathogenic cytokines, including IFNγ, TNF and IL6 (Fig. 4d). Typically, enrichment scores were greater for anti-CTLA4 treatment as compared to anti-PD-1 treatment, for several key pathways, including IFNγ response and TNF signalling (Fig. 4d, e and Supplementary Fig. 9a).

Next, we investigated the impact of combination anti-CTLA4/anti-PD-1 treatment on key pro-inflammatory pathways in comparison with anti-CTLA4 and anti-PD-1 monotherapies. Although the normalised enrichment score (NES) for IFNγ response was higher following anti-CTLA4 treatment (NES = 1.91, *P* = 1.9 × 10^−7^) than in anti-PD-1 treatment (NES = 1.6, *P* = 1.8 × 10^−3^), overall enrichment scores for combination treatment (NES = 1.91, *P* = 4.3 × 10^−8^) was similar to anti-CTLA4 monotherapy (Fig. 4f). However, the expression of some key IFNγ-responsive genes, including *Ccl5*, *Cxcl9*, *Cd40*, *Irf4*, *Irf5*, *Gbp8* and *Gbp4*, were further increased by combination therapy compared to either monotherapy (Supplementary Table 1). Other pathways, including IL6 JAK/STAK signalling and TNF signalling via NFκB, were mostly enriched to broadly comparable levels across the 3 different treatments (Supplementary Fig. 9b). Taken together, these data indicate that anti-PD-1 and anti-CTLA4 monotherapies regulate a very similar spectrum of genes and biological pathways in the colon during CPI-induced colitis, although the magnitude of the transcriptional response tends to be greater with anti-CTLA4 than anti-PD-1, and for some genes is further increased when anti-CTLA4 and anti-PD-1 are combined. These data are consistent with clinical observations, which show that anti-CTLA4 is a more potent driver of colitis than anti-PD-1^27^.

### High-resolution single-cell transcriptomics reveals colonic lymphocyte remodelling and emergence of polyfunctional, cytolytic lymphocyte responses in CPI-induced colitis

**Fig. 5:**
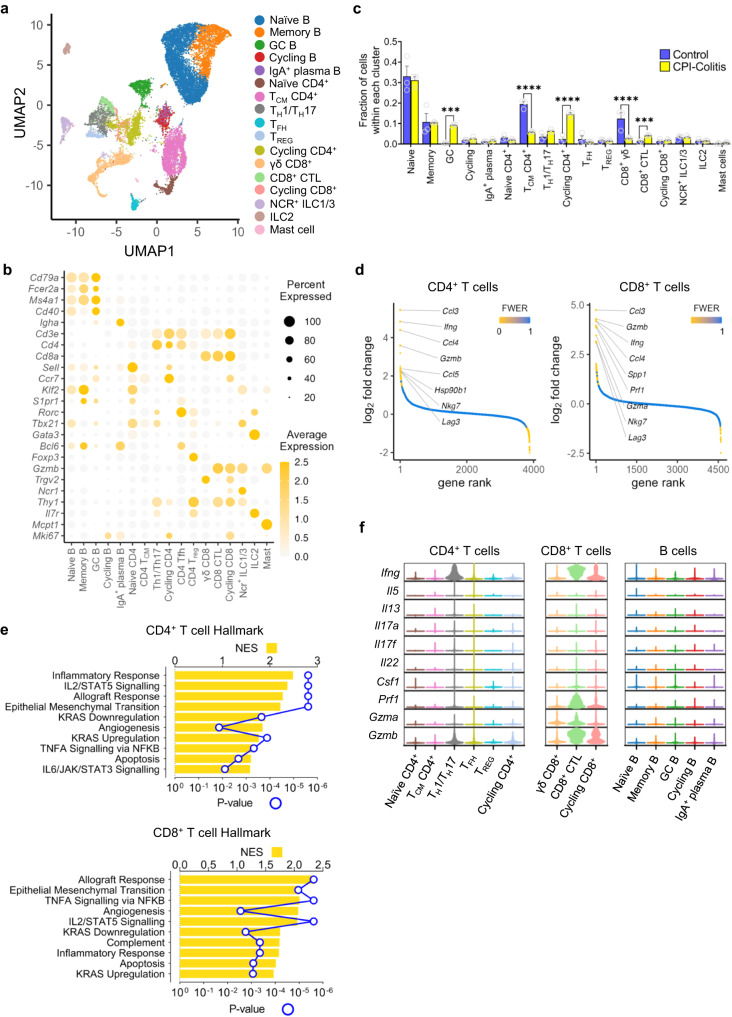
Transcriptomic landscape of colonic lymphocytes in CPI-induced colitis at single-cell resolution. **a** UMAP plots of the 17 lymphocyte populations labelled according to Louvain clustering, coloured based on SingleR cell-type assignments and split by the two conditions under consideration. **b** Dot plot of the key genes used to identify each of the 17 clusters as shown by average expression and percentage expressed in each cluster. **c** Average proportional changes shown across all clusters in wildtype female 6-week-old mice with CPI-induced colitis (*n* = 3) vs control wildtype female 6-week-old mice (*n* = 3). Bar graph showing mean with SEM and Two-way ANOVA with Sidak’s multiple comparison test used whereby ***P = 0.0004 and ****P < 0.0001. **d** Chair plots showing differentially expressed transcripts ranked by decreasing log fold change and coloured by estimated false discovery rate (FDR) across all CD4^+^ and CD8^+^ T cell clusters in wildtype female 6-week-old mice with CPI-induced colitis (*n* = 3) vs control wildtype female 6-week-old mice (*n* = 3). **e** Pathways, identified using GSEA Hallmark, upregulated in across all CD4^+^ and CD8^+^ T cell clusters in wildtype female 6-week-old mice with CPI-induced colitis (*n* = 3) vs control wildtype female 6-week-old mice (*n* = 3). *P* value adjustment for multiple comparisons was applied with the Benjamini and Hochberg method. **f** Violin plots showing the expression levels of cytokines across CD4^+^ and CD8^+^ T cell and B cell clusters in wildtype female 6-week-old mice with CPI-induced colitis (*n* = 3) vs control wildtype female 6-week-old mice (*n* = 3).

To further probe immune mechanisms of CPI-induced colitis, we performed single-cell RNA-sequencing (scRNA-seq) from FACS purified live CD45^+^ lymphocytes from the colons of mice with CPI colitis and control mice. We identified 17 different immune clusters based on their differing gene expression, based on established curated scRNA-seq databases^18,33,34^ (Fig. 5a, b). This initial clustering was based on the gene expression of canonical immune markers to identify B cells (*Cd19, B220*), CD4^+^ T cells (*Cd3, Cd4*), CD8^+^ T cells (*Cd3, Cd8*), mast cells (*Mcpt1*) and innate lymphoid cells (ILCs) (defined by the lack of any of these markers). B cells within our dataset were comprised of naïve (*Cd79a, Fcer2a, Ms4a1, Cd40* and *Sell*), memory (*Cd79a, Fcer2a* and *Ms4a1)*, germinal centre B cells (*Cd79a, Fcer2a, Ms4a1* and *Cd40*), cycling B cells (*Mki67*) and IgA^+^ plasma B cells (*Igha* and *Bcl6*) (Fig. 5b). CD4^+^ T cells clusters were then further subclustered into naïve (*Sell*, *Ccr7, Klf2* and *S1pr1*), central memory T cells (CD4^+^ T_CM_) (*Ccr7*), T_H_1/T_H_17 (*Cd4, Rorc, Tbx21*), cycling CD4^+^ T cells (*Mki67*), CD4^+^ follicular helper T cells (*Bcl6, Rorc*) and CD4^+^ regulatory T cells (*Foxp3*). CD8 T cells clusters which were identified were γδ CD8^+^ T cells (*Trgv2*), cytotoxic CD8^+^ T cells (*Gzmb, Thy1*) and cycling CD8^+^ T cells (*Mki67*) (Fig. 5b). ILCs were subclustered into NCR^+^ ILC1/ILC3s (*Ncr1, Thy1, Tbx21, Gzmb*) and ILC2 clusters (*Il7r, Gata3*) (Fig. 5b). Cluster analysis demonstrated shifts in the proportional abundance of colonic lymphocyte populations, including a significant expansion of cycling CD4^+^ T cells, cytotoxic CD8^+^ T cells and germinal centre B cells, and a significant decrease in the proportion of T_CM_ CD4^+^ T cells and γδ CD8^+^ T cells in CPI colitis (Fig. 5c). Although there was no significant change in the proportional abundance of the Foxp3^+^ CD4^+^ T_REG_ cluster between control and CPI colitis (Fig. 5c), there were a few qualitative changes in the transcriptome. For example, there was significant induction of *Traf1, Tnfrsf9, Hif1a, Slc2a1* and *Pim1* in T_REG_ clusters in CPI colitis in comparison with T_REG_ clusters in control mice. Transcript and biological pathway level changes in many of the colonic lymphocyte clusters identified in our model also strongly correlated with gene expression changes observed in analogous cell populations described in human CPI colitis^18^ (Supplementary Fig. 10a, b).

Analysis of the main lymphocyte lineages identified remodelling of the transcriptome in T cells and B cells (Fig. 5d and Supplementary Fig. 11a), with significant upregulation (Bonferroni corrected *P* value < 0.001) of *Ifng*, *Gzmb*, *Gzma, Nkg7*, and the chemokines *Ccl3*, *Ccl4* and *Ccl5* in both CD4^+^ and CD8^+^ T cell compartments in CPI-induced colitis (Fig. 5d). In B cells, there was upregulation of transcripts involved in protein synthesis and endoplasmic reticulum stress, such as *Calr*, *Hspd1*, *Hspa5*, in keeping with B cells adopting a secretory state in CPI-induced colitis (Supplementary Fig. 11a).

In both CD4^+^ and CD8^+^ T cells, GSEA Hallmark pathway analysis identified significant enrichment of processes such as inflammatory responses, IL2/STAT5 signalling, allograft rejection, TNF signalling and apoptosis in CPI colitis (Fig. 5e). GSEA PID pathway analysis identified significant enrichment of type 1 inflammatory cytokine pathways, including the IL12 pathway, and in CD4^+^ T cells there was also enrichment of IL23 pathway signalling (Supplementary Fig. 11b). In B cell clusters the most upregulated biological pathways were Myc target and Wnt signalling (Supplementary Fig. 11c). Cluster-specific analysis of lymphocytes in CPI colitis demonstrated expression of *Ifng* across multiple T cell clusters, including T_H_1/T_H_17, cycling CD4^+^ T cells, cytotoxic CD8^+^ T cells and cycling CD8^+^ T cells (Fig. 5f). These clusters also expressed high levels of cytotoxicity molecules (*Gzma*, *Gzmb* and *Prf1*) (Fig. 5f).

### IFNγ producing CD4^+^ and CD8^+^ T cell populations express multiple pro-inflammatory cytokines and cytotoxicity molecules in CPI colitis

**Fig. 6:**
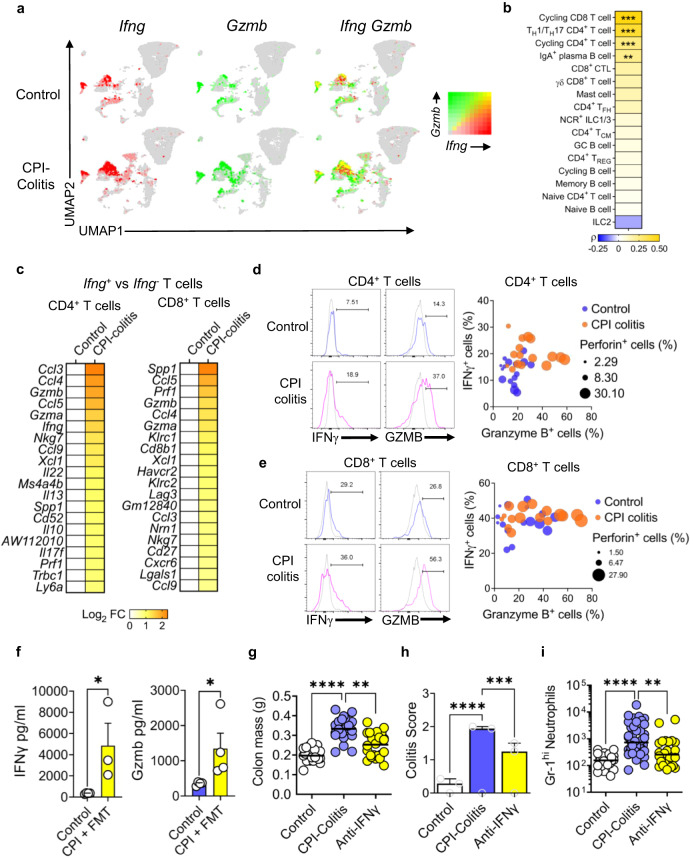
CPI-induced colitis exhibits a profound and increased cytotoxic phenotype. **a** UMAP plot and **b** the Pearson correlation showing the increased occurrence of *Ifng* and *Gzmb* expressing cells in CPI-induced colitis (*n* = 3) vs control samples (*n* = 4). *P* value adjustment for multiple comparisons was applied with the Benjamini and Hochberg method. **c** Heatmap of cytokine and chemokine expression shown by log_2_ fold changes between CPI-induced colitis (*n* = 3) and control samples (*n* = 4) in colonic CD4^+^ and CD8^+^ T cell clusters. **d** Representative flow cytometry histograms and bubble plots showing the percentage of granzyme B, IFNγ and perforin expressing CD3^+^ CD4^+^ T cells and **e** CD3^+^ CD8^+^ T cells in wildtype female 6-week-old mice with CPI-induced colitis (*n* = 16) and control wildtype female 6-week-old mice (*n* = 16). **f** Concentrations of IFNγ and granzyme B were determined via Luminex of supernatants from cultured colonic lamina propria cells at 2 × 10^6^/ml for 24 h with plate bound anti-CD3 (2 µg/ml) for untreated Balb/C wildtype female 6-week-old mice (Control: *n* = 4) and wildtype female 6-week-old mice treated with CPI colitis model (CPI + FMT: *n* = 4). Mean and standard deviation are shown. *P < 0.05 two-sided Mann–Whitney U T test performed. **g** Colon mass (Control: *n* = 21) or wildtype female 6-week-old CPI colitis mice treated with an isotype control (CPI colitis: *n* = 21) or anti-IFNγ (Anti-IFNγ: *n* = 24), Kruskal-Wallis Test with Dunn’s multiple corrections performed where **P = 0.0014 and ****P < 0.0001. **h** Average colitis score, using the same previous colitis score parameters (Control: *n* = 7) or wildtype female 6-week-old CPI colitis mice treated with an isotype control (CPI colitis: *n* = 17) or anti-IFNγ (Anti-IFNγ: *n* = 8). Bar graph showing mean with SEM and Two-way ANOVA with Tukey’s multiple comparison test used whereby ***P = 0.0003 and ****P < 0.0001. **i** Number of Gr-1^hi^ neutrophils of untreated wildtype female 6-week-old Balb/C mice (Control: *n* = 25) or wildtype female 6-week-old CPI colitis mice treated with an isotype control (CPI colitis: *n* = 43) or anti-IFNγ (Anti-IFNγ: *n* = 24) Kruskal–Wallis Test with Dunn’s multiple corrections performed where **P = 0.0025 and ****P < 0.0001.

Given the prominent IFNγ footprint observed in CPI colitis, we further probed the transcriptome of *Ifng*-expressing lymphocytes in CPI colitis in comparison with their *Ifng*-expressing counterparts in control mice. As well as being expanded in CPI colitis, *Ifng*-expressing lymphocytes co-expressed *Gzmb*, and this was found to be most highly co-expressed and correlated within the cycling CD8 T cells, T_H_1/T_H_17 and cycling CD4^+^ T cells clusters (Fig. 6a, b and Supplementary Fig. 12a). We, also, tested for differential abundance of *Gzmb*^*+*^ *Ifng*^*+*^ cells in each cell population and found that the populations with the most increased expression of both *Gzmb*^*+*^ *Ifng*^*+*^ genes were NCR^+^ ILC1/3 s, T_H_1/T_H_17, CD8^+^ γδ T cells, CD8^+^ CTLs and cycling CD4^+^ T cells (Supplementary Fig. 12b).

In comparison with *Ifng*-expressing CD4^+^ T cells in control mice, the top 20 most highly upregulated transcripts in *Ifng*-expressing CD4^+^ T cells in CPI colitis, included chemokines (*Ccl3, Ccl4, Ccl5, Ccl9*), other cytokines (*Il22, Il13, Il17f, Il10, Spp1*), and cytotoxicity molecules (*Gzma, Gzmb, Prf1, Nkg7*) (Fig. 6c). In CPI colitis, *Ifng*-expressing CD8^+^ T cells upregulated a similar pattern of transcripts among the most highly upregulated 20 transcripts (albeit with a more limited repertoire of cytokines than CD4^+^ T cells) (Fig. 6c).

To determine whether these changes were observed at protein level, we performed multiparameter flow cytometry. An increased proportion of colonic T cells co-expressing IFNγ granzyme B and perforin was observed in CPI colitis, especially in CD4^+^ and CD8^+^ T cells (Fig. 6d, e, Supplementary Fig. 13a, b). Similar findings were observed in the supernatants of lamina propria mononuclear cells (LPMCs) stimulated with agonistic anti-CD3 antibodies. In LPMCs from mice with CPI colitis there was significantly increased production of IFNγ and granzyme B in comparison to control mice (Fig. 6f and Supplementary Fig. 14a).

To determine whether IFNγ was functionally important in CPI colitis, we administered neutralising anti-IFNγ monoclonal antibodies, or isotype-matched control antibodies, to mice during induction of CPI colitis. Antibodies were administered simultaneously at the same time as combination CPI at weeks 0, 1 and 2. In keeping with IFNγ playing an important role in CPI colitis, neutralisation of this cytokine significantly reduced colon mass, improved histological appearances, and reduced infiltration of Gr-1^hi^ neutrophils (Fig. 6g–i).

### IFNγ producing T cell clusters have increased expression of co-stimulatory and immune checkpoint molecules

**Fig. 7:**
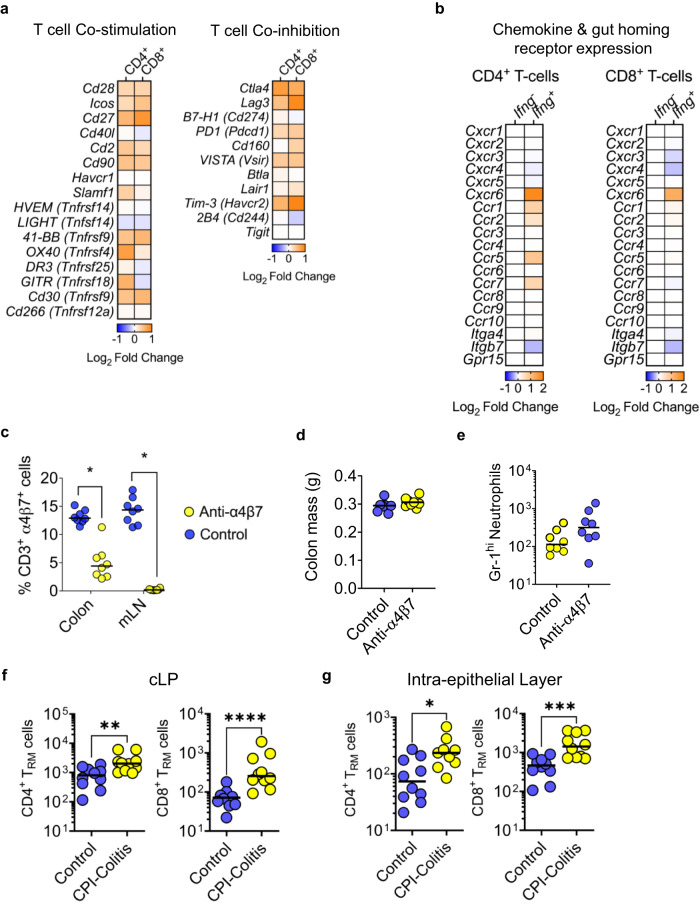
CPI-induced colitis is mediated by CXCR6 expressing tissue-resident T cells. **a** Heatmap of co-stimulatory and co-inhibitory expression from *Ifng*^+^ expressing CD4^+^ and CD8^+^ T cells in comparison to *Ifng*^*-*^-expressing CD4^+^ and CD8^+^ T cells. **b** Heatmap of chemokines and gut homing gene expression from *Ifng*^+^ expressing CD4^+^ and CD8^+^ T cells in comparison to *Ifng*^*-*^-expressing CD4^+^ and CD8^+^ T cells. **c** Percentage of CD3^+^ α4β7^+^ in the colon and mesenteric lymph node (mLN), **d** colon mass and **e** number of Gr-1^hi^ neutrophils in wildtype female 6-week-old CPI colitis mice treated with an isotype control (*n* = 8) or anti-α4β7 depleting antibody (*n* = 8). **f** Number of CD4^+^ and CD8^+^ T cells from the colonic lamina propria and **g** intraepithelial layer of untreated wildtype female 6-week-old Balb/C mice (Control: *n* = 10) and CPI + FMT (CPI colitis: *n* = 10). For both **f**, **g**, a two-tailed Mann–Whitney *U* Test was used where *P = 0.0172 **P = 0.0039 ***P = 0.0007 and ****P < 0.0001.

To further analyse the phenotype of IFNγ producing lymphocytes in CPI colitis, we examined their expression of co-stimulatory molecules, co-inhibitory molecules, chemokine receptors and gut-homing integrins. In both CD4^+^ and CD8^+^ T cells, there was an increased expression of co-stimulatory molecules in IFNγ producing lymphocytes in CPI colitis. In CD4^+^ T cells this included classical co-stimulatory molecules, such as *Cd28* and *Cd2*, and numerous members of the TNF receptor superfamily, including *Tnfrsf4*, *Tnfrsf18* and *Tnfrsf9* (Fig. 7a) There was a similar pattern of co-stimulatory molecule upregulation in CD8^+^ T cells, although *Cd27* was the most upregulated receptor in these cells (Fig. 7a). In CPI colitis, IFNγ producing lymphocytes also upregulated co-inhibitory receptors. In CD4^+^ T cells, the most upregulated checkpoints molecules, included *Ctla4* (fold change = 2.2, FDR = 7.0 × 10^−46^), *Hacvr2* (Tim-3, fold change 1.8, FDR = 3.7 × 10^−58^) and Lag3 (fold change = 1.7, FDR = 1.2 × 10^−38^). In CD8^+^ T cells *Hacvr2* (fold change 2.6, FDR = 1.8 × 10^−61^) and *Lag3* (fold change = 2.5, FDR = 6.7 × 10^−27^) were the most upregulate checkpoint molecules (Fig. 7a and Supplementary Fig. 15a). These data imply that both CD4^+^ and CD8^+^ IFNγ producing lymphocytes are very tightly regulated in CPI colitis.

To further evaluate the phenotype of *Ifng*-expressing lymphocytes in CPI colitis, we compared chemokine and gut-homing receptors in cells in comparison with lymphocytes that do not express *Ifng*. In both CD4^+^ cells and CD8^+^ T cells, the most upregulated chemokine receptor was *Cxcr6 (*6.3-fold, FDR = 2.1 × 10^−105^ in CD4^+^ T cells and 2.2-fold, FDR, and 3.6-fold, 1.1 × 10^−19^ in CD8^+^ T cells), and in the case of CD8^+^ T cells, *Cxcr6* was the only upregulated chemokine receptor (Fig. 7b and Supplementary Fig. 16a, b). Gut homing receptors, including *Itga4* and *Itgb7* that encode the classical gut homing integrin heterodimer α4β7 was downregulated in both *Ifng*-expressing CD4^+^ and CD8^+^ lymphocytes (Fig. 7b). These data maintained the hypothesis that tissue-resident cells, or cells arising from tissue-resident cells, are key drivers of CPI colitis rather than newly recruited T cells, especially since *Cxcr6* was the most highly upregulated chemokine receptor in both *Ifng*-expressing CD4^+^ and CD8^+^ lymphocytes. To determine whether de novo recruitment of lymphocytes is required to initiate colitis, and since vedolizumab is an effective drug used for patients with CPI-induced colitis, we administered anti-integrin α4β7 mAbs to mice prior to, and during, induction of CPI colitis. Although anti-integrin α4β7 mAbs substantially reduced the number of integrin α4β7 expressing CD3^+^ T cells in the colon and mesenteric lymph nodes (Fig. 7c), it failed to prevent or even reduce the severity of CPI colitis (Fig. 7d, e). These data are consistent with an important pathogenic role of CXCR6^+^ lymphocytes likely arising from tissue resident memory cells in CPI-induced colitis, rather than lymphocyte populations that have been newly recruited to the gut^18,19^. To evaluate this, tissue resident memory (defined by their surface marker expressions of CD44^+^ CD25^−^ CD69^+^ CD103^+^ CXCR6^+^) CD4^+^ and CD8^+^ T cells were analysed using flow cytometry in CPI colitis in cLP and intraepithelial layers of the colon of mice (Supplementary Fig. 17a). Consistent with CXCR6 being the most highly upregulated chemokine, we found that CD69^+^ CD103^+^ CXCR6^+^ CD4^+^ and CD8^+^ T cells were expanded in both the cLP and intraepithelial layer in mice with CPI colitis in comparison to control mice (Fig. 7f, g). Furthermore, analysis of the typical tissue resident markers, CD69^+^, CD103^+^ and CXCR6^+^, from CD44^+^ CD25^−^ CD4^+^ and CD8^+^ T cells, showed that there was a significant increase in CD69 and CXCR6 in both the cLP and intraepithelial layer in CD4^+^ and CD8^+^ T cells (Supplementary Fig. 17b–f). In the intraepithelial layer, a significant increase in CD103 in CD8^+^ T cells was observed.

We re-interrogated a previously published dataset of human colonic immune cells that had also been analysed by scRNA-seq^18^. In agreement with our findings, we observed increased expression of *IFNG*, *GZMB CXCR6* and *CTLA4* in T cell clusters in with patients with CPI colitis patients in comparison with healthy controls (Supplementary Fig. 18a, b).

### IL23 blockade suppresses IFNγ-producing CD4^+^ colonic T cells and attenuates the development of CPI colitis

**Fig. 8:**
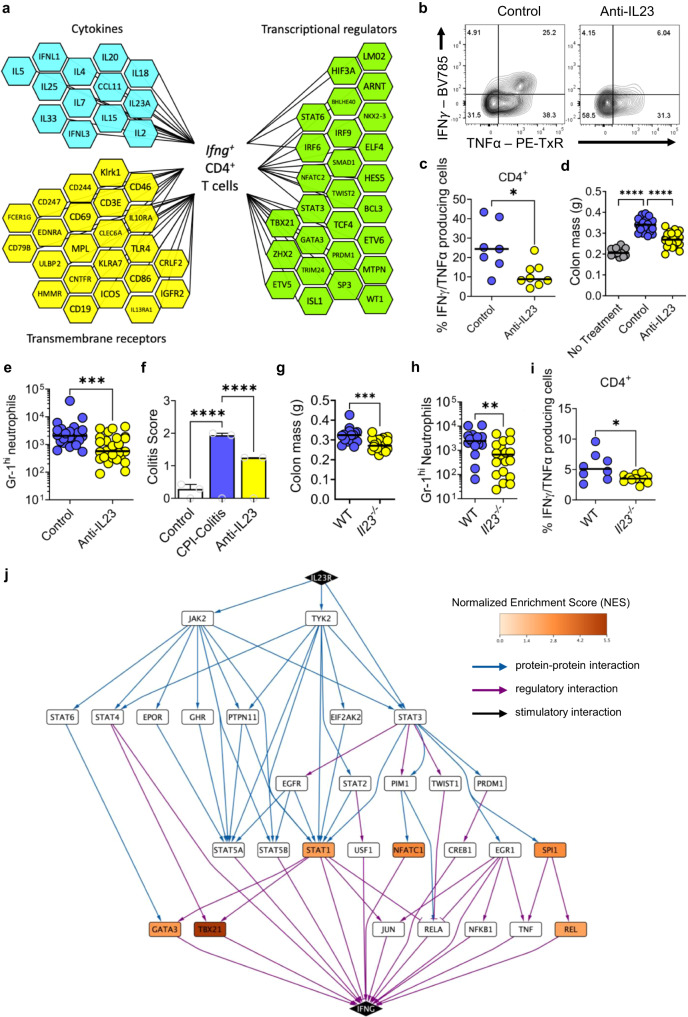
IL23 blockade attenuates the development of CPI colitis. **a** Network analysis, using Enhanced Causal Network using Qiagen IPA, of predicted upstream regulators of *Ifng*-expressing CD4^+^ T cell in CPI colitis. Multiple cytokines, transmembrane receptors and transcriptional regulators were predicted to significantly (FDR < 0.05) regulate *Ifng*-expressing CD4^+^ T cell. **b** Representative flow cytometry plot and **c** percentage of IFNγ^+^/TNFα^+^ CD4^+^ T cells from wildtype female 6-week-old CPI colitis mice treated with an isotype control (*n* = 7) or an IL-23 blocking antibody (*n* = 8). Two-tailed Mann–Whitney Test performed where *P = 0.019. **d** Colon mass, in either control mice (*n* = 12) or wildtype female 6-week-old CPI colitis mice treated with an isotype control (*n* = 23) or an IL-23 blocking antibody (*n* = 24). Kruskal-Wallis test with Dunn’s multiple comparison used where ****P < 0.0001. **e** Gr-1^hi^ neutrophil counts in either wildtype female 6-week-old CPI colitis mice treated with an isotype control (*n* = 24) or an IL-23 blocking antibody (*n* = 26), using a two-tailed Mann–Whitney Test to show ***P = 0.0006 and **f** Average colitis score, using the same previous colitis score parameters, in either control mice (*n* = 7) or wildtype female 6-week-old CPI colitis mice treated with an isotype control (*n* = 17) or an IL-23 blocking antibody (*n* = 8). Bar graph showing mean with SEM and Two-way ANOVA with Tukey’s multiple comparison test used whereby ****P < 0.0001. **g** Colon mass, using a two-tailed Mann–Whitney Test to show ***P = 0.0003, and **h** Gr-1^hi^ neutrophil counts in either wildtype female 6-week-old mice (*n* = 17) or female 6-week-old *Il23*^*−/−*^ mice (*n* = 20) treated with CPI colitis. A two-tailed Mann–Whitney Test to show **P = 0.005, **i** Percentage of IFNγ^+^/TNFα^+^ CD4^+^ T cells from CPI colitis treated wildtype female 6-week-old mice (*n* = 8) or female 6-week-old *Il23*^*−/−*^ mice (*n* = 12). A two-tailed Mann–Whitney Test to show *P = 0.0, **j** PathLinker network analysis of *Ifng*-expressing CD4^+^ T cell in CPI colitis showing the highest-ranking shortest paths involving differentially expressed genes linking *Il23r* to *Ifng* through regulatory and protein-protein interactions queried from OmniPath. Paths with wider edges rank higher with upregulated genes shown in orange, downregulated in blue.

There is a major unmet need to identify therapeutically mediators in CPI colitis, therefore, we conducted an Enhanced Causal Network Analysis (IPA) to identify upstream mediators responsible for activating the pattern of gene expression that we observed in *Ifng* expressing lymphocytes in CPI colitis. Analysis of cytokines predicted to activate *Ifng* expressing CD4^+^ T cells in CPI colitis included IL2 (*z* score 4.7, *P* < 1.0 × 10^−27^), IL7 (*z* score 3.8, *P* < 9.8 × 10^−19^), IL15 (*z* score 3.4, *P* < 5.2 × 10^−34^), IL18 (z-score 2.0, *P* < 4.0 × 10^−25^) and IL23A (z-score 2.6, *P* < 2.1 × 10^−31^) (Fig. 8a, Supplementary Table 2). Other predicted regulators included transcriptional regulators (TBX21, NFATC2, STAT3, IRF6 and IRF9) transmembrane receptors, like CD244, cytotoxicity receptors (NKG2D), CD69 and the complement receptor CD46 (Fig. 8a, Supplementary Table 2).

From a therapeutic perspective targeting cytokines predicted to activate these pathogenic cells is an attractive strategy, as many neutralising monoclonal antibodies have been, or are currently, in clinical development for other immune-mediated inflammatory diseases. IL23 is an especially attractive target in CPI colitis with multiple reagents being used to treat conventional IBD. To determine whether IL23 is functionally important in CPI colitis, we administered mAbs that neutralise p19 subunit of the IL23 heterodimer at the same time as combination immunotherapy. IL23 blockade reduced the number of IFNγ producing CD4^+^ T cells in the colon in CPI colitis and especially IFNγ/TNFα co-producing cells (Fig. 8b, c). The proportion of cytokine-producing CD8^+^ T cells were unaffected by the blockade of IL23 (Supplementary Fig. 19a, b). IL23 blockade attenuated key disease features, including significantly reduced colon mass, significantly reduced histology score and reduced recruitment of colonic neutrophils (Fig. 8d–f). Similar findings were observed in *Il23*^*−/−*^ mice following induction of disease, with reduced colon mass and reduced numbers of infiltrating neutrophils in comparison with control mice (Fig. 8g, h). Furthermore, CD4^+^ T cells had reduced number of IFNγ/TNFα co-producing cells (Fig. 8i and Supplementary Fig. 19c). The proportions of cytokine-producing CD8^+^ T cells were unaffected by the genetic ablation of *Il23* (Supplementary Fig. 19d–e).

To further understand how IL23 might regulate pathogenic effector CD4^+^ T cells in CPI colitis, we constructed a regulatory network predicted to be activated by IL23 based on downstream gene expression changes in *Ifng* expressing CD4^+^ T cells. The network identified activation of transcription factors (STAT3, NFATC1, T-bet), MAP kinase pathways (ERK1/2, p38 MAPK), signalling molecules (Phospholipase C gamma 2) and other kinases (PRKCD, Syk) implicated in inflammatory diseases (Fig. 8j), providing a potential regulatory roadmap for IL23-mediated regulation of IFN-expressing T cells in CPI colitis.

To investigate whether IL23 could be a viable target in human CPI-induced colitis, we interrogated a gene expression in the colon from a recently published dataset of patients developing CPI colitis following treatment with combination immunotherapy^32^. Consistent with our analyses there was significant upregulation of cytotoxicity molecules (*PRF1, GZMB*, *GZMA*), interferon-responsive genes (*CXCL10*), as well as significant upregulation of *IL23* and its key signalling molecule *STAT3* (Supplementary Fig. 19f). Together our data support the rationale for therapeutic targeting of IFNγ producing, polyfunctional T cells in CPI colitis, including neutralisation of their upstream regulators, such as IL23.

## Discussion

This study describes a model of CPI-induced colitis that resembles aspects of CPI colitis and provides new mechanistic insights into the immunopathogenesis of CPI-induced colitis. Preclinical models that accurately recapitulate clinically important irAEs are urgently needed to aid the search for effective, targeted therapies to treat these severe side effects. Other preclinical models of irAEs have been described but are hampered by their limited clinical or immunological resemblance to human disease. Depletion of T_REG_ cells and in vivo activation of co-stimulatory molecules together with CPIs can induce multi-system inflammation^35^, but is far removed from the immune context of irAEs. Indeed, there is no evidence of T_REG_ depletion in human CPI colitis^18^, as was observed in our model. Another published model relies on application of combination immunotherapy to mice following induction of graft versus host disease (GvHD) following adoptive transfer of human PBMCs to severely immunodeficient *Rag2*^*−/−*^ *Il2rg*^*−/−*^ mice^32^, which even in the absence of CPI-exposure may result in multi-organ inflammatory disease. Multiple irAEs can also be induced in mouse strains genetically prone to autoimmunity, such as B6/lpr mice, when combination immunotherapies are co-administered with complete Freund’s Adjuvant^36^. Although this model results in multiple organ toxicities, it is not known whether the resulting colitis has any immunological similarities with human CPI colitis, as the immunopathology has yet to be described in detail.

In the model we have described there are close clinical, histological, transcriptional, and immunological similarities with human CPI colitis. We identified polyfunctional mucosal CD4^+^ and CD8^+^ T cells that co-produce IFNγ, other pro-inflammatory cytokines (e.g. IL22, IL17A) and cytotoxic molecules, including granzyme B, as key effector cells in CPI colitis. Crucially, these pathogenic, polyfunctional T cells share features with colonic T cell clusters described in patients with CPI colitis and resemble T cell phenotypes observed in anti-viral responses and anti-tumour immunity^18,19,37–40^. Crucially, we identify the IL23 and IFNγ as a functionally important cytokine axis in CPI colitis.

Our data, and data from human studies, provide insights into the likely origins of pathogenic effector T cells in CPI colitis. Both *Ifng*-expressing CD4^+^ and CD8^+^ clusters highly expressed *Cxcr6*, consistent with pathogenic effector CD4^+^ and CD8^+^ T cells arising from T_RM_ cells^18,19,41^. Luoma et al. identified a marked overlap in TCR clonotypes of the key colitis-associated T cell clusters with T_RM_ cells^18^, implying that the effector and cycling clusters are likely derived from proliferating T_RM_ cells, rather than newly recruited cells. These observations are consistent with our data showing that prevention of T cell trafficking with α4β7 blockade failed to prevent the onset of colitis. Although our data, and the 2 published studies describing T cell phenotypes in patients with CPI colitis^18,19^, all point to a key role for T_RM_ cells as potentially important cells giving rise to pathogenic effector cells, these data are potentially in contradiction to the apparent efficacy of vedolizumab, which blocks the gut homing integrin α4β7^42^. There are a number of potential explanations for this apparent contradiction. So far, the efficacy of vedolizumab in CPI colitis has only been reported in small, retrospective, observational studies, and accordingly its effectiveness in this setting should be regarded with caution^43^. It is also possible that this disconnect is underpinned by differences in the cellular mediators at different stages of this disease. For instance, it is possible that disease is initially triggered by effector lymphocytes originating from proliferating T_RM_ cells, in which case anti-trafficking drugs like vedolizumab would not prevent disease onset. However, once colitis is fully established, it is plausible that further inflammation may arise by newly recruited T cells that migrate via the α4β7 integrin/MAdCAM-1 axis. Notably, the 28 patients treated with vedolizumab all had long-standing colitis and had already received on average 3 months of high-dose steroid therapy, and additional treatment with infliximab in many patients prior to vedolizumab treatment^42^.

Another important characteristic of the IFNγ producing, polyfunctional T cells identified in this study is that they significantly upregulated multiple co-stimulatory and co-inhibitory receptors, indicating that these cells are very tightly regulated. This phenomenon has also been observed in the expanded T-cell populations described in patients with CPI colitis^18,19^. We hypothesise that under physiological circumstances these potentially pathogenic cells are kept in check by engagement of immune checkpoint molecules. However, in patients undergoing checkpoint blockade, these crucial regulatory mechanisms are lost, and these potentially pathogenic effector cells escape repression enabling them to mediate disease. Given that these cells also express high levels of co-stimulatory molecules, they may be pre-primed for rapid activation and induction of pro-inflammatory effector responses. Notably, CTLA4 was one of the most highly expressed immune checkpoint molecules in both CD4^+^ and CD8^+^ T cells and was expressed at higher levels than PD-1, indicating that CTLA4 may be especially important in restraining the pathogenic potential of these cells. Notably, cancer patients treated with anti-CTLA4 antibodies have increased penetrance and severity of colitis than patients treated with anti-PD-1 antibodies^8,27^. Data from our preclinical model support this notion, with mice receiving CTLA4 blockade experiencing more severe colitis than mice treated with anti-PD-1 alone.

Computational approaches predicted several cytokines, including IL23 as potentially important upstream regulators of mucosal T cells in CPI colitis. Here, we show that IL23 blockade, or genetic ablation, reduced pathogenic cytokine production by CD4^+^ T cells, but not CD8^+^ T cells in CPI colitis, and significantly attenuated disease development. Notably, targeting IL23 did not completely prevent colitis, probably because pathogenic cytokine production was only reduced in CD4^+^ T cells, but not CD8^+^ T cells. Nevertheless, IL23 is a plausible and attractive target for CPI colitis. As well as being highly expressed in the colon of patients with CPI colitis^32^, its receptor (IL23R) is also expressed by multiple T cell clusters identified in single-cell sequencing experiments of patients with CPI colitis^18^. Notably, mucosal T_RM_ cells also have increased expression of the IL23R^44^. IL23 plays an important role in other immune-mediated inflammatory diseases, especially at the barrier surfaces, and clinical reagents targeting this cytokine are already used for conventional IBD, with promising results^45,46^. Unlike with blockade of IFNγ, which would impact the anti-tumour immunity^47^, IL23 blockade is unlikely to impede systemic, anti-cancer immunity, and neutralisation of the IL23 might even promote favourable cancer outcomes^48–50^. A case report of successful treatment of two cases of CPI colitis following treatment with ustekinumab, which blocks the p40 subunit, common to both IL12 and IL23, further supports the rationale for this approach. Other potentially targetable cytokines implicated in causal network analysis include IL7, IL15 and IL18. One of the key transcriptional regulators predicted to activate the gene expression changes observed in polyfunctional mucosal lymphocytes was NFATc2, which is also highly upregulated in the colon of patients with CPI colitis. Calcineurin inhibitors, that target NFATc2 also appear to be effective in patients with CPI colitis.

In agreement with observations in human disease, susceptibility to CPI-induced colitis is dependent on the composition of the intestinal microbiota^20–23^. Barrier surfaces, especially at the colon, are challenged with maintaining immunological restraint against a multitude of different commensal bacteria that vary across individuals and time^51^, whilst remaining poised to repel invading pathogens. Within the limits of the sparsity of existing human data charting longitudinal changes in CPI-induced colitis, we note a degree of commonality and difference between the microbiota composition of our model and that seen in patients. For example, depletion of *Lachnospiraceae*, which was reduced in our model, is associated with onset of colitis in patients with CPI-induced colitis^20,52^. In another study, which only reported baseline microbiota profiles, a higher relative abundance of *Bacteroidota* was correlated with protection against CPI-induced colitis^22^. Conversely, FMT treatment led to increased *Bacteroidota* abundance, which was associated with the onset of checkpoint-induced colitis as seen in our model. Microbiota analysis of our model points to potential mechanisms through which colitis might be triggered. The most over-expressed genus post-FMT was *Alistipes*, which has been shown to promote colonic inflammation in an IL-10 knockout model of colitis^53^. Conversely, post-FMT fecal samples were depleted in short-chain fatty acid-producing bacteria, including *Lachnospiraceae, Roseburia* and *Oscillibacter*. Short-chain fatty acids augment intestinal barrier function^54^, regulate the size and function of the colonic regulatory T cell pool^55^ and down-regulate pro-inflammatory macrophage activity via inhibition of histone deacetylases^56^. Other studies have indicated a possible remedial role of probiotic strains of bacteria, such as *Bifidobacteria* and *Lactobacillus*, in murine models of DSS colitis with the presence of an anti-CTLA4 blockade antibody^21,57,58^. In our model, *Bifidobacteria* are not present in the microbiota of mice before or after induction of colitis. *Lactobacilli* are present at baseline, but do not alter significantly over the course of colitis. It is possible that the absence of *Bifidobacteria* in our model facilitates the onset of colitis and that administration of a probiotic strain such as *Bifidobacterium breve* might ameliorate the phenotype. It is also conceivable that the mechanism of colitis in our model is independent of previous studies due to the differences in using DSS compared to the pro-inflammatory microbiota colitis induction from the TRUC mice. In our data, transcriptomic analysis of the colon following colonisation with colitis-permissive microbiota did trigger activation of some immune pathways, however, in the presence of intact immune checkpoint regulation overt colitis was averted. However, when these key checkpoints are inhibited, dysbiosis primes unchecked activation of polyfunctional, cytotoxic lymphocyte responses. Accordingly, immune checkpoint molecules are likely important immune rheostats in the regulation of host perception of microbial colonisation in the colon. Patients with germline deletions in the *CTLA4* gene spontaneously develop severe enterocolitis^59,60^.

Although our study has important strengths, including assessment of the functional importance of proximal and effector cytokines in a disease model resembling key aspects of human disease, we also acknowledge some limitations. Our single-cell sequencing experiment predominantly focussed on the LP compartment, which could potentially overlook important changes occurring in the intraepithelial layer. Notably, in patients with CPI colitis the major changes were upregulation of cytotoxicity programs in cycling and effector T cell subsets residing in the LP^18^. Our model has also been conducted in the absence of a concomitant tumour, which could potentially influence immune responses in other organs, including the colon.

In conclusion, this study provides fresh mechanistic insights into immune regulation at the mucosal barrier surfaces with important implications for the prevention and treatment of CPI-induced autoimmunity. In the context of a colitis-permissive intestinal microbiota, combination of immune checkpoint blockade with anti-CTLA4 and anti-PD-1 results in the emergence of IL23-dependent, polyfunctional, cytolytic, CD4^+^ and CD8^+^ T-cell responses that are functionally important in CPI colitis development. Effective strategies to target these pathogenic effector cells could herald improved clinical outcomes for patients affected by this severe, immune-mediated complication of life-saving CPI therapy.

## Methods

### Animal husbandry

C57BL/6 and Balb/C wild-type mice (both Charles River C57BL/6 code: 027 and Balb/C code: 028) and *Il23*^*−/−*^ mice (ModelOrganisms NM-KI-00057) were sourced commercially. TRUC mice have been described previously^24,25,61^. All mice were co-housed in specific pathogen-free facilities at King’s College London Biological Services Unit, Imperial Hammersmith CBS, or Charles River Laboratories. All animal experiments were performed in accredited facilities in accordance with the UK Animals (Scientific Procedures) Act 1986 (Home Office Licence Numbers PPL: 70/6792, 70/8127, 70/7869, P8999BD42).

### Isolation of cells

Mice were culled using an approved Home Office Schedule 1 method at the end of an experiment. Mouse colons were excised and placed in cold Phosphate Buffered Saline (PBS) solution. Colonic LPMCs were isolated, as described previously^62^. Briefly, the epithelium was removed in HBSS (Invitrogen) supplemented with 5 mM of EDTA and 10 mM HEPES (Fisher Scientific). For investigating cells in the intraepithelial layer, the method was adapted from a previous protocol^63^. The tissue was digested in HBSS with 2% FCS and supplemented with 0.5 mg/ml collagenase D, 10 μg/ml DNase I and 1.5 mg/ml dispase II (all Roche). The digested lymphocyte-enriched population was harvested using a 40%–80% Percoll (GE Healthcare) gradient centrifugation for cLP and a 40%–70% for the cell suspension from the intraepithelial layer.

### Flow cytometry

Single suspension extracted cells, as described above, were plated into flow cytometry tubes (Sarstedt) at a concentration of 1 × 10^6^ per ml. Cells were stimulated with 50 ng/ml phorbol 12-myristate 13-acetate (PMA), 1 μg/ml ionomycin, 2 μM monensin (all Sigma Aldrich) for 3–4 h as indicated. (Sigma Aldrich) FcR receptor-blocking antibodies were added before staining with antibodies. Surface staining antibodies were added with live/dead stain (Invitrogen). For intracellular staining, cells were fixed and permeabilised using the Foxp3 fixation/permeabilization buffer kit (Thermo Fisher) according to the manufacturer’s instructions. Samples were acquired using a BD LSRFortessa (BD Biosciences). Sample data was recorded in FCS 3.0 data format using BD FACSDiva 6.0 software (BD Biosciences). Analysis of the data was performed using FlowJo software (Treestar Inc., Ashland, OR, USA). Supplementary Table 3 provides the antibodies used and their dilutions.

### Histology

Whole mouse colon samples were rolled using the Swiss roll technique^64^ fixed in 10% paraformaldehyde and embedded in paraffin blocks. 3 μm sections were stained for haemotoxylin and eosin.

### FMT treatment

Fecal content extracted from the caecum of colitis-observed TRUC mice was spun down and reconstituted in sterile PBS with 25% glycerol and then 200 µl was orally gavaged into mice at the beginning of FMT treatment. All mice used for these FMT experiments were 6 weeks of age at the start of experiments. All experimental mice were co-housed in specific pathogen–free facilities at King’s College London Biological Services Unit or Imperial Hammersmith CBS. Untreated non-FMT control mice were housed in a separate box to FMT treated mice.

### In vivo murine antibody treatment

Mice used were 6–7 weeks old at the start of experiments and gender-matched. Female mice were used in most experiments for this study. Mice treated with immune checkpoint blockade drugs were intraperitoneally administered anti-CTLA4 (9H10, BioXCell), using doses of 200 μg, and anti-PD-1, (RMP1-14, BioXCell) at a dose of 250 μg, once per week^40^. All experimental mice were co-housed in specific pathogen–free facilities at King’s College London Biological Services Unit or Imperial Hammersmith CBS. Untreated control mice were housed in a separate box from CPI + FMT treated mice. Mice treated with depleting antibodies were intraperitoneally administered once a week, at the same time of giving anti-CTLA4 and anti-PD-1 antibodies, either 500 μg anti-TNFα (XT3.11, BioXCell), 500 μg anti-IFNγ (H22, BioXCell) or 150 μg anti-IL-23(p19) (G23-8, BioXCell). Control-isotype clones used were 2A3 (rat IgG2a) and HRPN (rat IgG1).

### Luminex multiplex assay

Colonic LPMCs were cultured at 2 × 10^6^/ml for 24 h with plate-bound anti-CD3 (2 µg/ml) at 37 °C. Analysis of cytokine concentrations were done using Milliplex MAP Mouse CD8^+^ T Cell Magnetic Bead Panel Premix 15 Plex (MCD8MAG48K-PX15) according to the manufacturer’s instructions. A MAGPIX system (Luminex) was used for data acquisition.

### DNA extraction and 16S rRNA gene sequencing of the microbiota

Fecal samples were collected from untreated controls, FMT treated and FMT with CPI-treated mice at baseline and at one, two and 3-weeks and frozen at –80 °C pending DNA extraction. Samples were thawed to room temperature. DNA was extracted from stool using the DNeasy Powerlyzer Powersoil Kit (Qiagen) following manufacturer instructions with an additional bead beating step for three minutes at speed 8 in a Bullet Blender Storm (Chembio Ltd, St Albans, UK). DNA was quantified using a Qubit Fluorometer (ThermoFischer, UK) and was stored at −80 °C awaiting downstream analysis.

Sample libraries were prepared according to Illumina’s 16S Metagenomic Sequencing Library Preparation protocol. with modifications as previously described. Two modifications to this protocol were implemented. Firstly, the V1-V2 regions of the 16S rRNA gene were amplified using primers as follows: the forward primer was constructed with (5’−3’) the Illumina i5 sequencing primer (TCGTCGGCAGCGTCAGATGTGTATAAGAGACAG) and the gene-specific primer combination (28F-YM: GAGTTTGATYMTGGCTCAG + 28F-Borrellia: GAGTTTGATCCTGGCTTAG + 28F-Chloroflex: GAATTTGATCTTGGTTCAG + 28F-Bifido: GGGTTCGATTCTGGCTCAG) in a 4:1:1:1 ratio. The reverse primer was constructed with (5’−3’) the Illumina i7 sequencing primer (GTCTCGTGGGCTCGGAGATGTGTATAAGAGACAG) and the gene-specific reverse primer (388 R: TGCTGCCTCCCGTAGGAGT). Secondly, a clean-up and normalisation step was performed on the index PCR reactions using AMPure XP magnetic beads (Beckman Coulter, Indiana, USA). Libraries were quantified using the NEBNext Library Quant Kit for Illumina (New England Biolabs, Hitchin, UK). Sequencing was performed on an Illumina MiSeq platform (Illumina Inc., Saffron Walden, UK) using the MiSeq Reagent Kit v3 (Illumina) and paired-end 300 bp chemistry.

Sequencing data were processed using the DADA2 package in R, following the DADA2 pipeline as previously described^65^. Taxonomy was assigned by mapping sequence variants to the SILVA v138.1 database^66^. Pre-processed assigned sequence variants (ASVs) data were cleaned and filtered using the decontam (v1.16) pipeline removing contaminating DNA features^67^, Data were further filter before analysis, removing samples with less than 1000 sequence reads, in an approach to remove sequencing artefacts. Alpha-diversity metrics were then calculated on the filtered data, before prevalence filtering removed ASVs that were not present in at least 15% of samples. After prevalence filter, a total of 188 ASVs were compared in beta diversity and differential abundance analyses. A combination of R packages was used to analyse and visualise fecal microbiota sequencing data including Phyloseq^68^, Vegan and ggplot2. To assess changes in alpha-diversity, mixed effects models^69^ compared Shannon’s diversity index, adjusting for sequencing depth. Aitchison distance was used for beta diversity analyses^70^ after centre log-ratio data transformation (CLR)^71^; principal coordinates analyses (PCoA) were generated to visualise the (dis)similarity between groups. Permutational multivariate analysis of variance (PERMANOVA) was used to statistically compare groups. Differential abundance analysis of ASVs was calculated using ANCOM-BC^72^ with Holm-Bonferroni *p* value correction and a mixed effects model (adjusting for aforementioned covariates) with false discovery rate (FDR) correction. A *p* value of 0.05 or a *q* value of 0.05 were considered significant^73^.

### RNA extraction

RNA was extracted from colon segments or purified cells with Qiazol reagent (Invitrogen). In this method, distilled solutions of RNA were purified using chloroform and isopropanol and eluted with ethanol before being resuspended in RNAse-free water. RNA was cleaned up using the Qiagen RNeasy Microkit and performed the DNA clean-up protocol as listed in the manufacturer’s guidelines. RNA samples were then checked for quality, contamination and concentration using a NanoDrop, Qubit spectrophotometer and Bioanalyzer. RNA with only a RIN score of above 7 were used for RNA sequencing. RNA was then stored at −80 °C awaiting further analysis.

### Bulk RNA-seq data analysis

The quality of the raw library files was inspected with fastQC. Raw reads were trimmed and filtered to remove adaptor contamination and poor-quality bases using trimmomatic^74^. The resulting read files were mapped to the GRCm38 (mouse) or GRCh38 (human) genome assembly using Hisat2^75^ with default parameters. The number of reads mapping to the genomic features annotated in Ensembl^76^ with a MAPQ score higher than or equal to 30 was calculated for all samples using htseq-count^77^ with default parameters. Data for Ensembl genes with no associated ENTREZ gene identifier were discarded from downstream analyses; the read counts for Ensembl genes mapped to the same ENTREZ gene identifier were summed up sample wise. Differential expression analysis between sample groups was performed in R using the Wald test as implemented in the DESeq2 package^63^, adjusting for the spread of the transcriptomic profiles along the first principal component. P-values were adjusted for multiple testing with the Benjamini and Hochberg procedure^64^. The P- and corresponding FDR values were re-estimated empirically with fdrtool^40^, when the *P* value distributions showed that the assumptions of the test were not met.

### Droplet-based single-cell RNA sequencing and library preparation

Colonic LPMCs from mice were initially sorted using a FACS Aria machine (BD Biosciences) based on live CD45^+^ gates and taken immediately to be run on the 10×. Cells were suspended at 1 × 10^6^/mL in PBS and 10,000 cells were loaded onto the Chromium^TM^ Controller instrument within 15 min after completion of the cell suspension preparation using GemCode Gel Bead and Chip, all from 10x Genomics (Pleasanton, CA), and following the manufacturer’s recommendations. Briefly, cells were partitioned into Gel Beads in Emulsion in the Chromium^TM^ Controller instrument where cell lysis and barcoded reverse transcription of RNA occurred. Libraries were prepared using 10x Genomics Library Kits (3’ end V3 kit) and sequenced on an Illumina HiSeq2500 according to the manufacturer’s recommendations. Read depth of more than 200 million reads per library, or an approximate average of 20,000 reads per cell was obtained with a recovery of 5000 cells.

### Single-cell RNA gene sequencing analysis

The raw 10X Genomics sequencing libraries were processed using the Cell Ranger suite v.3.0.1 to demultiplex base call files, generate single-cell feature counts for each library, and finally combine these data into one feature by barcode matrix. Read alignment and gene expression quantification made use of the CellRanger pre-built mouse (mm10 v. 3.0.0) reference data. The individual UMI count matrices were normalised to the same effective sequencing depth before they were aggregated. The merged UMI count matrix was imported in R v.3.6.1 and quality checks were carried out to mitigate the effects of technical artefacts. Filtering steps were taken to remove genes detected in less than 3 barcodes, and barcodes with: (1) more than 12,400 UMIs (determined as three median absolute deviations above the median barcode library size); or (2) less than 198 detected genes (determined as three median absolute deviations below the median number of genes for all barcodes after log2 transformation); or (3) expression of the epithelial cell adhesion molecule (*Epcam*) or collagen alpha-1(I) chain (*Col1a1*) that would suggest infiltration of epithelial cells or fibroblasts; or (4) co-expression of genes encoding chains of the CD3 complex (*Cd3d*, *Cd3e* and *Cd3g*) and those of the B cell antigen receptor (*Cd79a*, *Cd79b*) to limit further the impact of multiplets. The filtered dataset was imported into Seurat v3.1.5^78^. and the anchor-based integration workflow was followed to account for biological and technical batch differences between mice and sequencing libraries. For each sample, the UMI counts were normalised using the LogNormalize method, and the most highly variable genes were identified using the *vst* method. Up to 10,000 integration anchor cells were identified for each pair of count matrices after dimensionality reduction to 20 coordinates via Canonical Component Analysis. These anchor sets guided the process of integrating the individual sample data into one shared space with all genes passing initial quality checks. The expression data were scaled after regressing out the following sources of biological and technical variation: mouse id, sequencing library preparation and sequencing batches, number of UMIs detected for each cell and percent of UMIs for mitochondrial genes in each cell. After running Principal Component Analysis, 30 coordinates were used to embed the whole dataset into two dimensions using UMAP.

For clustering purposes, first the *k* = 20 nearest neighbours of each cell were determined using the Euclidean distance of their expression profiles projected onto the first 30 principal components previously identified. A shared nearest neighbour graph was then built to represent the neighbourhood overlap between cell pairs using the Jaccard similarity index. From this graph, 24 cell clusters (identified with integers from 0 to 23) were computed by the Louvain algorithm for modularity optimisation with a resolution parameter equal to 0.8. Markers for each cluster were identified by differential expression analysis using MAST^79^. Only genes expressed in at least 5% of the cells in the cluster under consideration or the rest of the cell population were tested. Genes were considered differentially expressed if the absolute value of the log fold change was greater than 0.25 and the Bonferroni-adjusted *P* value was <0.001.

Clusters were annotated to broad cell types using the SingleR (v.1.0.6) package^80^ and the Immunologic Genome Project (ImmGen) transcriptomic datasets for sorted populations of mouse immune cells^81^. To this end, SingleR was used to calculate cluster-level gene expression profiles from the individual cells data, and then to classify them using its correlation-based iterative algorithm with default parameter settings. More fine-grained cell-type labels were assigned based on the combination of domain knowledge and the patterns of differential expression between each cluster and the remaining ones.

Quality checks of these data in combination with the results of the differential expression tests highlighted the following discrepancies. The T cell clusters 4 and 9 significantly over-expressed *Cd4* and *Cd8a* at the same time. Comparison of their expression levels across the cells of each such cluster confirmed the occurrence of separate *Cd4*^*+*^ and *Cd8a*^*+*^ sub-populations. Cluster 11 could not be confidently labelled, being considered a B cell population and a T-cell population before and after fine-tuning, respectively. Inspection of *Cd79a* and *Cd3e* expression across the cells revealed two separate groups of T and B cells. Therefore, cells in clusters 4 and 9 with higher *Cd4* expression were kept in the original clusters, while those with higher *Cd8a* expression were manually assigned to two new clusters identified as 24 and 25. Cells in cluster 11 expressing *Cd79a* were placed into a new cluster identified as 26, while those expressing *Cd3e* were kept in cluster 11.

### Pathway analyses

Enrichment analyses by over-representation tests were conducted using IPA (QIAGEN). Enrichment of the input DEG lists (FDR < 0.05 for bulk RNA-Seq data and FWER < 0.001 for scRNA-Seq) was calculated against user-defined background lists including all the genes tested for differential expression. Patterns of increased or decreased activity for individual pathways or upstream regulators were calculated automatically based on the concordance between the IPA knowledgebase and the directionality of gene expression changes in the input data^62^.

Enrichment of the hallmark gene signatures was assessed using gene-set enrichment analysis (GSEA) against the genes tested for differential expression. Ranked gene lists were built by taking the geometric mean between the absolute value of the log fold change and the p-value from the differential expression tests following log10 transformation and change of sign. The sign of the log fold change was then multiplied to the score. These calculations were executed in R (v.3.6.1) using the packages msigdbr and fsgsea with default parameters.

### Analysis of publicly available datasets

Log-normalised data from the Nanostring PanCancer Immune Profiling Panel used to sample mucosal biopsies from healthy controls and patients with CPI colitis were downloaded from the corresponding publication^32^. Differential expression analysis between the two donor groups was performed with the DESeq2 package^63^ and p-value adjustment for multiple comparisons was applied with the Benjamini and Hochberg procedure. GSEA was used to evaluate the enrichment of the mouse homologues of the human genes differentially expressed (FDR < 0.05) against the expression changes in the mouse model of CPI colitis presented in this study.

Processed data from droplet-based scRNA-seq profiles of CD45^+^ immune cells from healthy donors and patients with CPI colitis were downloaded from NCBI GEO (Series GSE144469). The UMI count matrices were filtered and normalised as described in the original publication using Seurat. The resulting dataset was integrated following the reciprocal PCA workflow and expression data were finally scaled by regressing out the percentage of mitochondrial transcripts and the number of UMIs detected in each cell. Cell-type assignments and UMAP coordinates were kindly provided by the authors. Average normalised gene expression for each population in CPI colitis was calculated and compared to equivalent data from the mouse data presented here by means of Spearman correlation. These expression profiles were also scanned for enrichment of hallmark signatures using single-sample GSEA. Spearman correlation between the output enrichment scores from the mouse and human data was also estimated.

### Statistical analysis

Results are expressed as median ± IQR or mean ± SEM. Data were analysed using Two-way Student’s *t* test, Two-way Mann–Whitney *U* test, and two-way ANOVA Kruskal-Wallis test, as appropriate, using GraphPad Prism 10.0 (GraphPad Inc., USA). No data were excluded from the analyses. All in vitro experiments were repeated twice independently with similar results. All in vivo experiments needed 5–8 mice in each group and were carried out at least three times.

### Reporting summary

Further information on research design is available in the Nature Portfolio Reporting Summary linked to this article.

### Supplementary information

Supplementary Information

Reporting Summary

### Source data

Source Data

## Data Availability

All raw data in this study are available in the Source Data file, which has been uploaded with this manuscript. NGS data presented in the manuscript have been made publicly available through the Gene Expression Omnibus (GEO) database. The GEO IDs are: GSE222843 “Transcriptomic profiling of an in vivo mouse model of immune checkpoint induced colitis” for the bulk RNA-seq data comparing untreated, FMT only, CPI only and FMT + CPI-treated wildtype mice in Fig. 3 (https://www.ncbi.nlm.nih.gov/geo/query/acc.cgi?acc=GSE222843). GSE241664 “Transcriptomic profile comparison of an in vivo model of dual combination immune checkpoint colitis or monotherapy immune checkpoint colitis” for the mouse RNA-seq data comparing the effects of mono and combination therapies in Fig. 4 (https://www.ncbi.nlm.nih.gov/geo/query/acc.cgi?acc=GSE241664). Lastly, GSE222959 “Single-cell transcriptomics reveals colonic lymphocyte remodelling and the emergence of polyfunctional, cytolytic lymphocyte responses in CPI-induced colitis” for the single-cell RNA-seq data comparing the CPI + FMT treated mice and untreated wildtype mice for Figs. 5–8 (https://www.ncbi.nlm.nih.gov/geo/query/acc.cgi?acc=GSE222959). The 16S rRNA gene data in Fig. 2 have been made publicly available and deposited on the ENA at the EBI with the accession number PRJEB65719. Source data are provided with this paper.
