# Supplementary material for: Immune checkpoint inhibitor-induced colitis is mediated by polyfunctional lymphocytes and is dependent on an IL23/IFNγ axis
Source: Nat Commun. 2023 Oct 23;14:6719. doi: 10.1038/s41467-023-41798-2 (PMC10593820; doi:10.1038/s41467-023-41798-2)
Supplement: Supplementary file 1 — Supplementary Information [file 41467_2023_41798_MOESM1_ESM.pdf]

Supplementary Information for “Immune checkpoint inhibitor-induced colitis is mediated by polyfunctional lymphocytes and dependent on IL23 and IFNγ”

By: Jonathan W. Lo et al.

Supplementary Figure 1

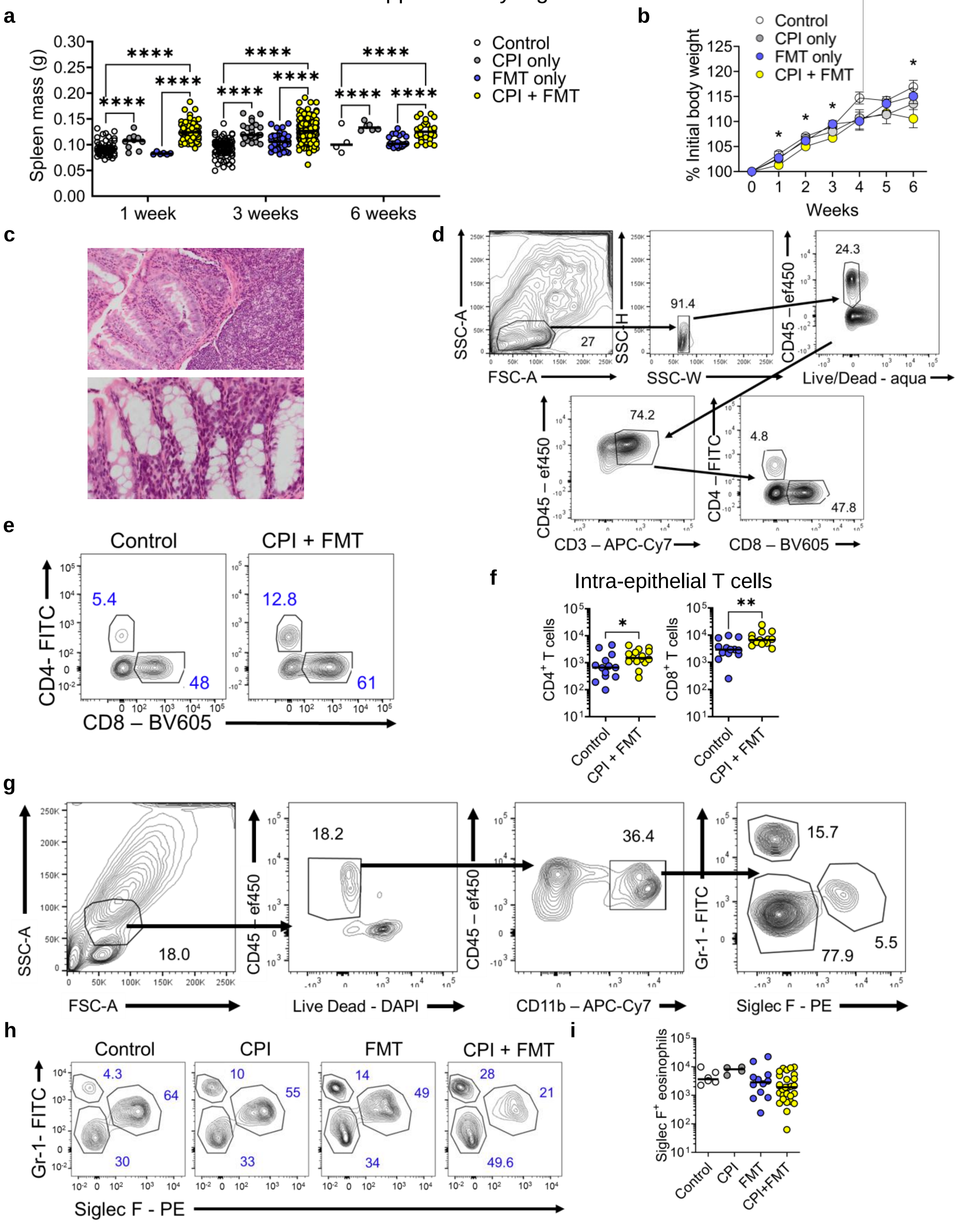

Supplementary Figure 1. The intestinal microbiota regulates susceptibility to immune checkpoint inhibitor-induced colitis
(a) Spleen mass and **(b)** change in body weight in wildtype female 6-week-old Balb/C mice at 1 week, 3 weeks and 6 weeks without treatment (Control: n=56 at 1 week, n=79 at 3 weeks, n=4 at 6 weeks), treatment with combination anti-CTLA4/anti-PD-1 (CPI: n=10 at 1 week, n=25 at 3 weeks, n=6 at 6 weeks), treatment with faecal microbiota (FMT: n=6 at 1 week, n=35 at 3 weeks, n=24 at 6 weeks) and wildtype female 6-week-old mice treated with both CPI and FMT (CPI + FMT: n=68 at 1 week, n=182 at 3 weeks, n=23 at 6 weeks). **(c)** Marked crypt apoptosis and lymphocyte infiltration of the lamina propria in mice treated with combination anti-CTLA/anti-PD-1 therapy and FMT. **(d)** Flow cytometry gating strategy used to identify $CD4^+$ and $CD8^+$ T cells in the lamina propria and intra-epithelial layer of the colon. **(e)** Representative flow plot and **(f)** number of $CD4^+$ and $CD8^+$ T cells from the intra-epithelial layer of the colon of Control (n=13) and CPI+FMT (n=15) treated wildtype female 6-week-old Balb/C mice. **(g)** Flow cytometry gating strategy used to identify Siglec F^+ eosinophils in the colon of mice. **(h)** Representative flow plot and **(i)** number of Siglec F^+ eosinophils (pre-gated on live $CD45^+$ $CD11b^+$ $Gr\text{-}1^-$) in the lamina propria of the colon in wildtype female 6-week-old Balb/C mice without treatment (control, n=6), treatment with combination anti-CTLA4/anti-PD-1 (CPI, n=3), treatment with faecal microbiota (FMT, n=13) and mice treated with both CPI and FMT (n=54). * P<0.05 ** P<0.01 *** P<0.001 **** P<0.0001 2-sided Kruskal-Wallis Test or 2-way ANOVA showing median.

Supplementary Figure 2

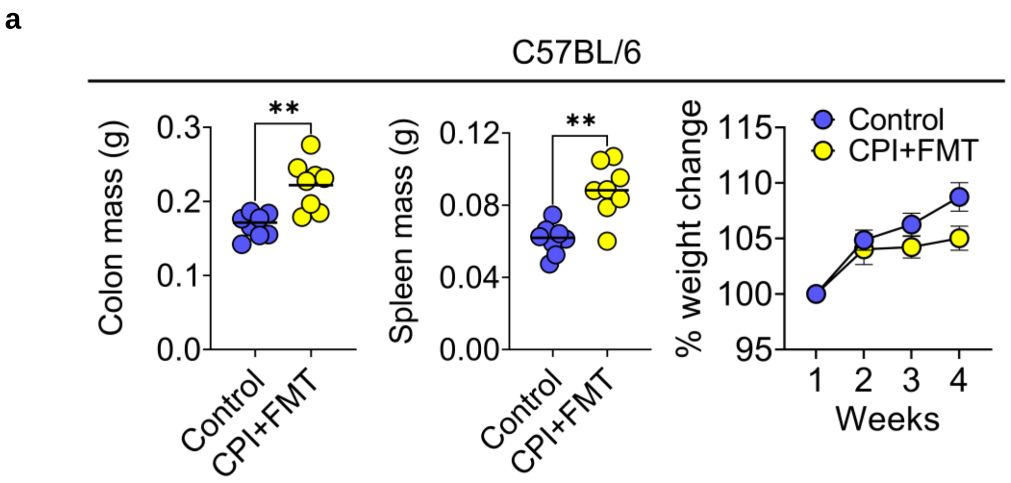

Supplementary Figure 2. C57BL/6 mice also develop CPI-induced colitis with combination CPI and FMT
(a) Colon mass, spleen mass and the percentage weight change between untreated 6-week-old C56BL/6 male mice (n=8) and 6-week-old C56BL/6 male mice treated with both CPI and FMT (n=8). Both colon and spleen had Mann Whitney Tests performed, for colon ** P=0.0011 and for spleen ** P=0.003

Supplementary Figure 3

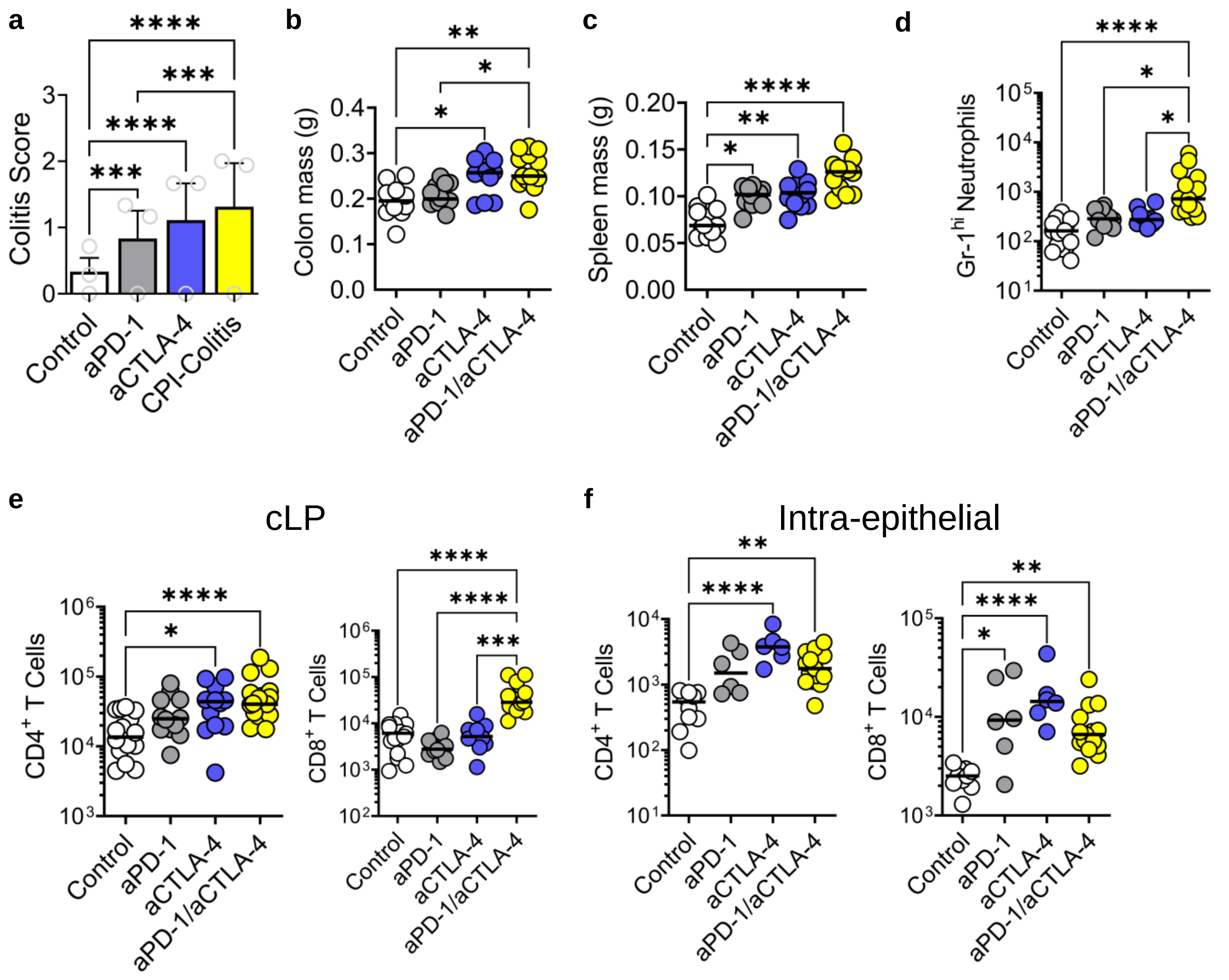

Supplementary Figure 3. Mice treated with combination checkpoint therapy develop worse CPI-induced colitis compared to monotherapy threated mice

(a) Summary average histological colitis score from untreated wildtype female 6-week-old Balb/C mice at 3 weeks without treatment (Control: n=7), treatment with anti-PD1 and FMT (aPD1: n=6), treatment with anti-CTLA4 and FMT (aCTLA4: n=6) and wildtype female 6-week-old Balb/C mice treated with combination anti-CTLA4/anti-PD1 and FMT (aPD1/aCTLA4: n=17). Colitis scored based on apoptosis (0-3), infiltrating lymphocytes (0-3), crypt abscess formation (0-3). **(b)** Colon mass, **(c)** spleen mass and **(d)** number of Gr-1^{hi} neutrophils in wildtype female 6-week-old Balb/C mice at 3 weeks without treatment (Control: n=12), treatment with anti-PD1 and FMT (aPD1: n=12), treatment with anti-CTLA4 and FMT (aCTLA4: n=12) and wildtype female 6-week-old Balb/C mice treated with combination anti-CTLA4/anti-PD1 and FMT (aPD1/aCTLA4: n=13). **(e)** Number of $CD4^+$ and $CD8^+$ T cells from the colonic lamina propria or **(f)** the intra-epithelial layer of wildtype female 6-week-old Balb/C mice at 3 weeks without treatment (Control: n=12), treatment with anti-PD1 and FMT (aPD1: n=12), treatment with anti-CTLA4 and FMT (aCTLA4: n=12) and wildtype female 6-week-old Balb/C mice treated with combination anti-CTLA4/anti-PD1 and FMT (aPD1/aCTLA4: n=13).

Supplementary Figure 4

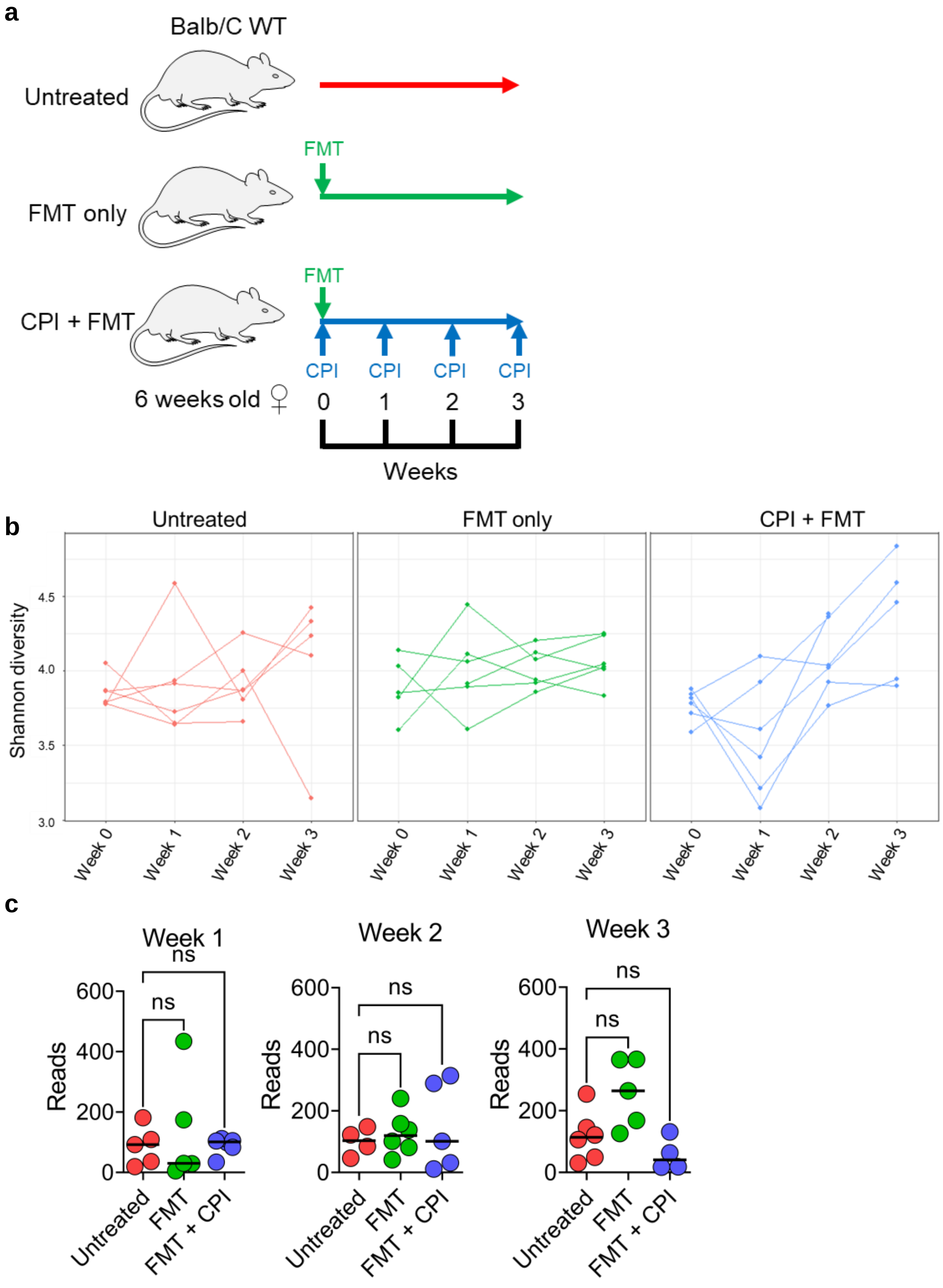

Supplementary Figure 4: Faecal microbiota transplantation alters the community composition of the intestinal microbiota

(a) Schematic of the experimental plan for the longitudinal FMT study. **(b)** Alpha diversity (Shannon diversity index) of the microbiota between untreated control female 6-week-old Balb/C wildtype mice (n=6), Balb/C wildtype female 6-week-old mice treated with FMT (n=6) and Balb/C wildtype female 6-week-old mice treated with CPI + FMT (n=6) longitudinally over a 3 week period. **(c)** Read counts of *Lactobacilli* between female 6-week-old Balb/C wildtype mice (n=6), Balb/C wildtype female 6-week-old mice treated with FMT (n=6) and Balb/C wildtype female 6-week-old mice treated with CPI + FMT (n=6) longitudinally over a 3 week period.

Supplementary Figure 5

a

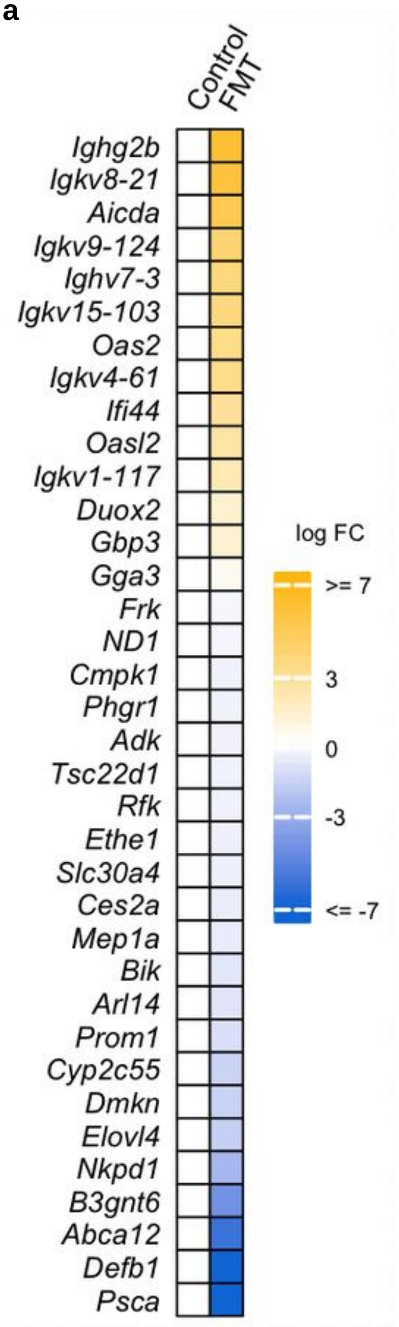

Supplementary Figure 5: Gene expression changes in the colon of WT mice following FMT
(a) DEGs (FDR<0.05) in the colon of Balb/C wildtype female 6-week-old mice following FMT (n=3) in comparison with control Balb/C wildtype female 6-week-old mice (n=4).

Supplementary Figure 6

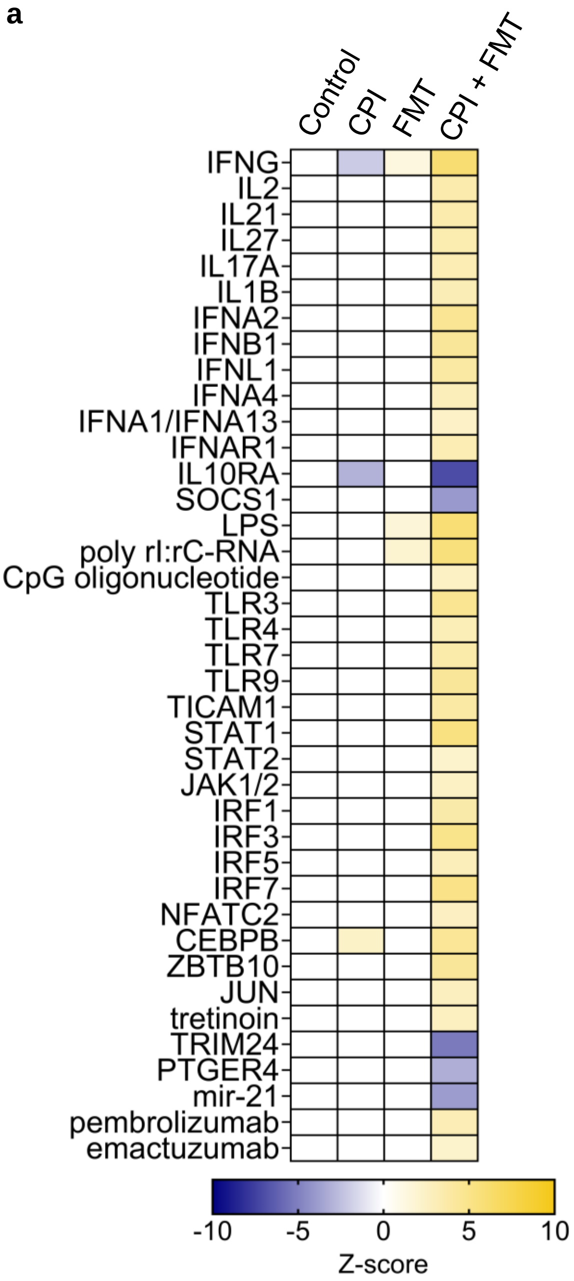

Supplementary Figure 6: The upstream regulators identified to predict the gene expression changes observed in CPI-induced colitis

(a) Upstream regulators predicted to control the gene expression changes observed in the colon of mice treated with CPI (n=4), FMT (n=3) and both treatments together (CPI+FMT, n=3), in comparison with control mice (n=4) were identified using IPA Upstream Regulator Analysis.

Supplementary Figure 7

a

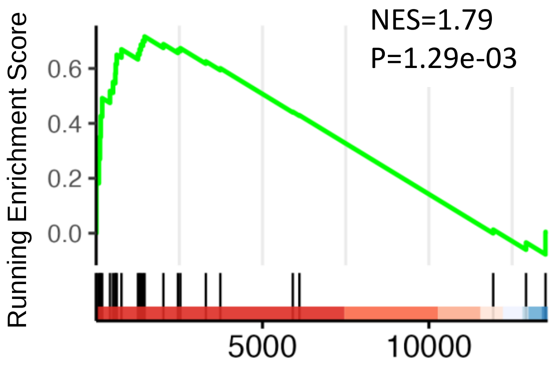

Supplementary Figure 7: Link between transcriptional features of CPI-induced colitis in human and mouse at bulk RNA-seq level

(a) GSEA results for the mouse homologs of the most significantly up-regulated genes in colon biopsies from patients affected by CPI-induced colitis. 32 human genes significantly up-regulated in CPI-induced colitis (log fold change > 1 and FDR < 0.05) were identified through differential expression analysis of a previously published dataset focusing on the nCounter PanCancer Immune Profiling Panel. The gene signature consisted of all their 39 mouse homologs expressed in Balb/C wildtype female 6-week-old mice (n=4) and in Balb/C wildtype female 6-week-old mice treated with FMT and anti-CTLA4/anti-PD-1 combination therapy (n=3). The mouse genes were ranked based on the estimated expression log fold changes between these conditions, using the control as reference. NES: Normalised Enrichment Score, P: P value of the gene set enrichment test.

Supplementary Figure 8

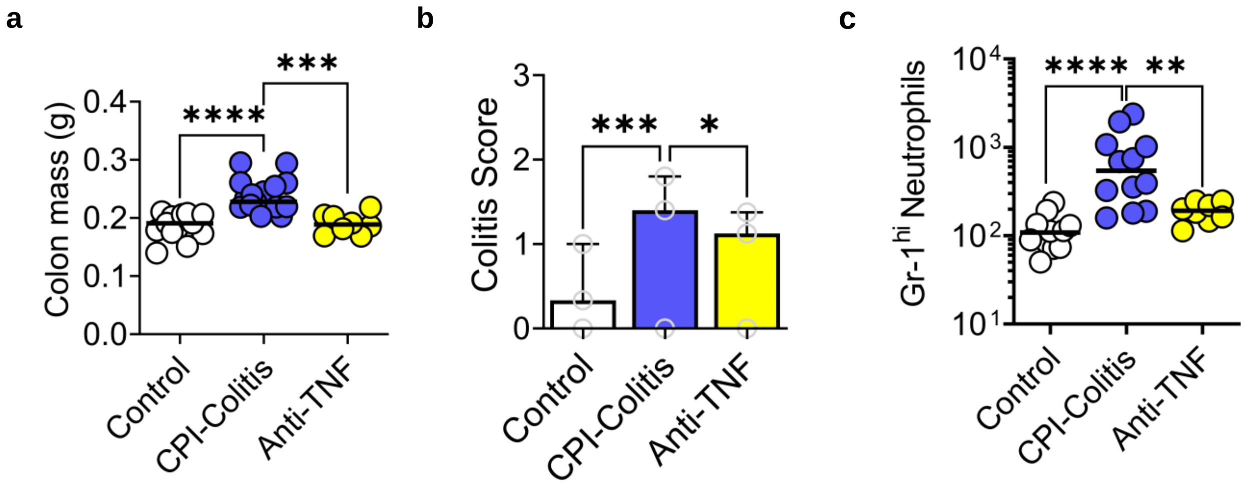

Supplementary Figure 8: Blockade of TNFα reduces the inflammation in CPI-induced colitis

(a) Colon mass, **(b)** colitis score, using the same previous colitis score parameters, and **(c)** number of Gr-1^{hi} neutrophils in untreated Balb/C wildtype female 6-week-old mice (Control: n=14), treatment with combination anti-CTLA4/anti-PD1 and FMT (CPI-Colitis: n=17) and treatment combination anti-CTLA4/anti-PD1 and FMT and anti-TNFα (Anti-TNF: n=8). * P<0.05 ** P<0.01 *** P<0.001 **** P<0.0001 two-sided Kruskal-Wallis Test showing median.

Supplementary Figure 9

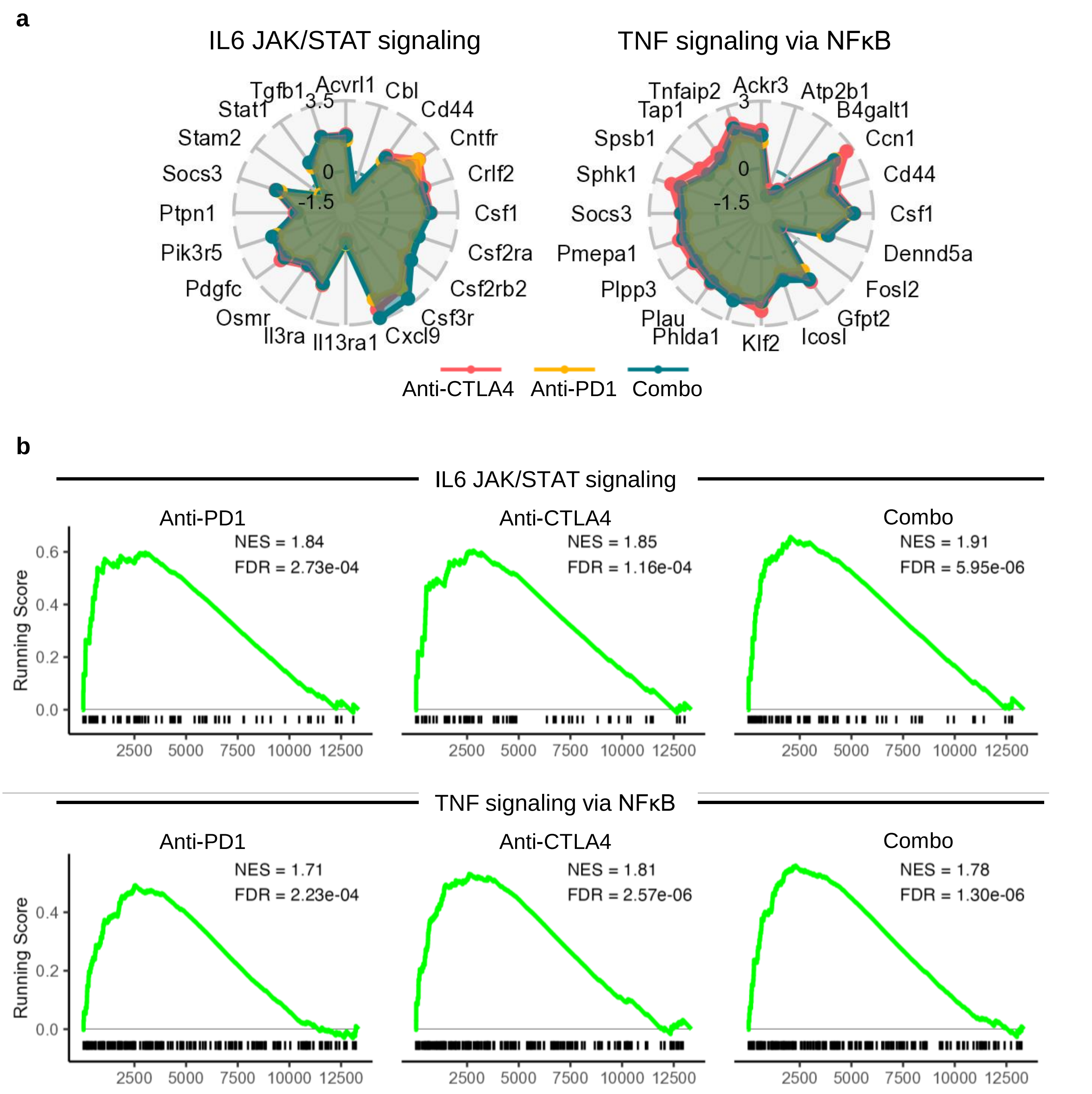

Supplementary Figure 9: Other inflammatory pathways are not altered in monotherapy or combination treated mice

(a) Radar plot showing log2 fold change for the top 20 genes associated to either the IL6 JAK/STAT pathway and TNF signalling via NFκB pathway in the gene expression changes in the distal colon of wildtype female 6-week-old mice with only anti-CTLA4 treatment (n=3), anti-PD1 treatment (n=3) or combination treatment (n=3), in comparison to control wildtype female 6-week-old mice (n=3). **(b)** GSEA running score for the IL6 JAK/STAT pathway and TNF signalling via NFκB pathway in the gene expression changes in the distal colon of wildtype female 6-week-old mice with only anti-CTLA4 treatment (n=3), anti-PD1 treatment (n=3) or combination treatment (n=3), in comparison to control wildtype female 6-week-old mice (n=3).

Supplementary Figure 10

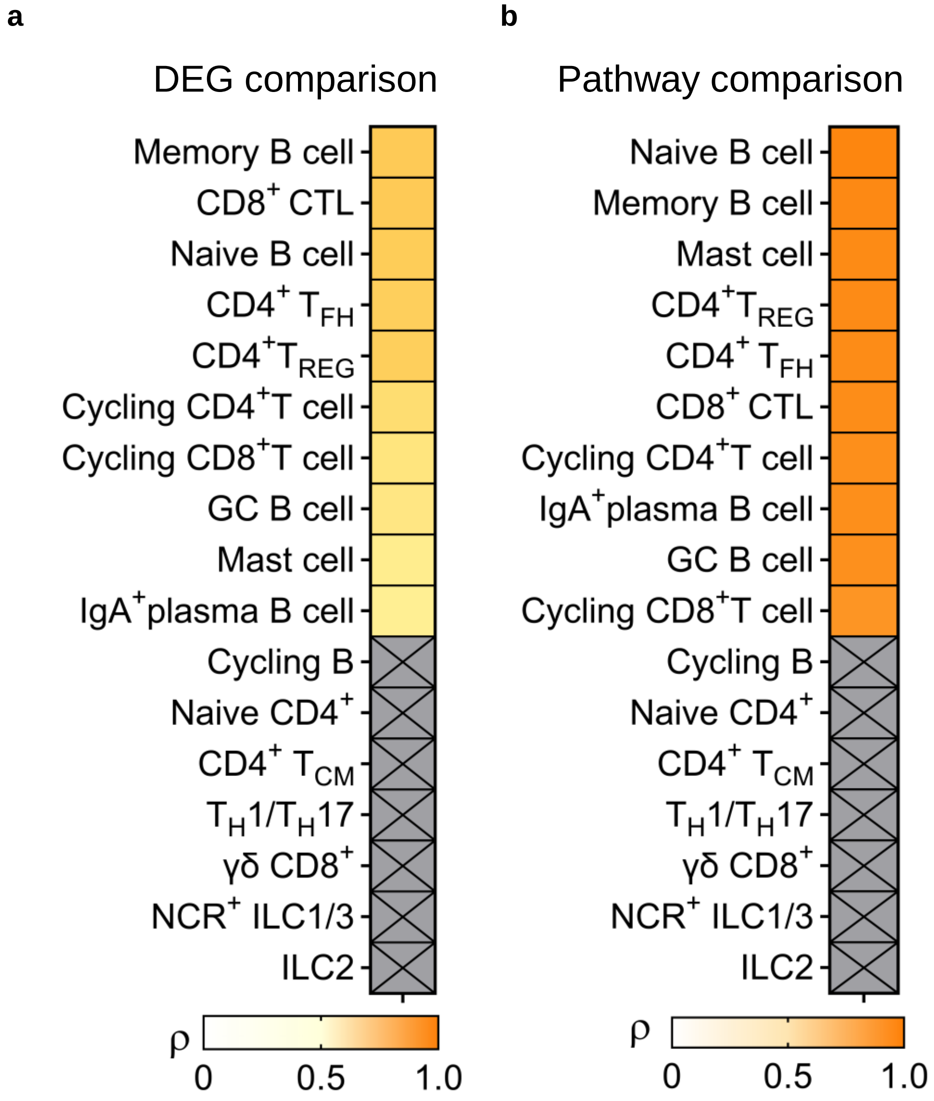

Supplementary Figure 10: Correlation between CPI-induced colitis in human and mouse at single cell RNA-seq level

(a) Heatmap showing the correlation of the average gene expression between the cell clusters identified in the mouse scRNA-seq dataset compared to the same corresponding human cell cluster **(b)** Heatmap showing the correlation of the Hallmark pathway between the cell clusters identified in the mouse scRNA-seq dataset compared to the same corresponding human cell cluster

Supplementary Figure 11

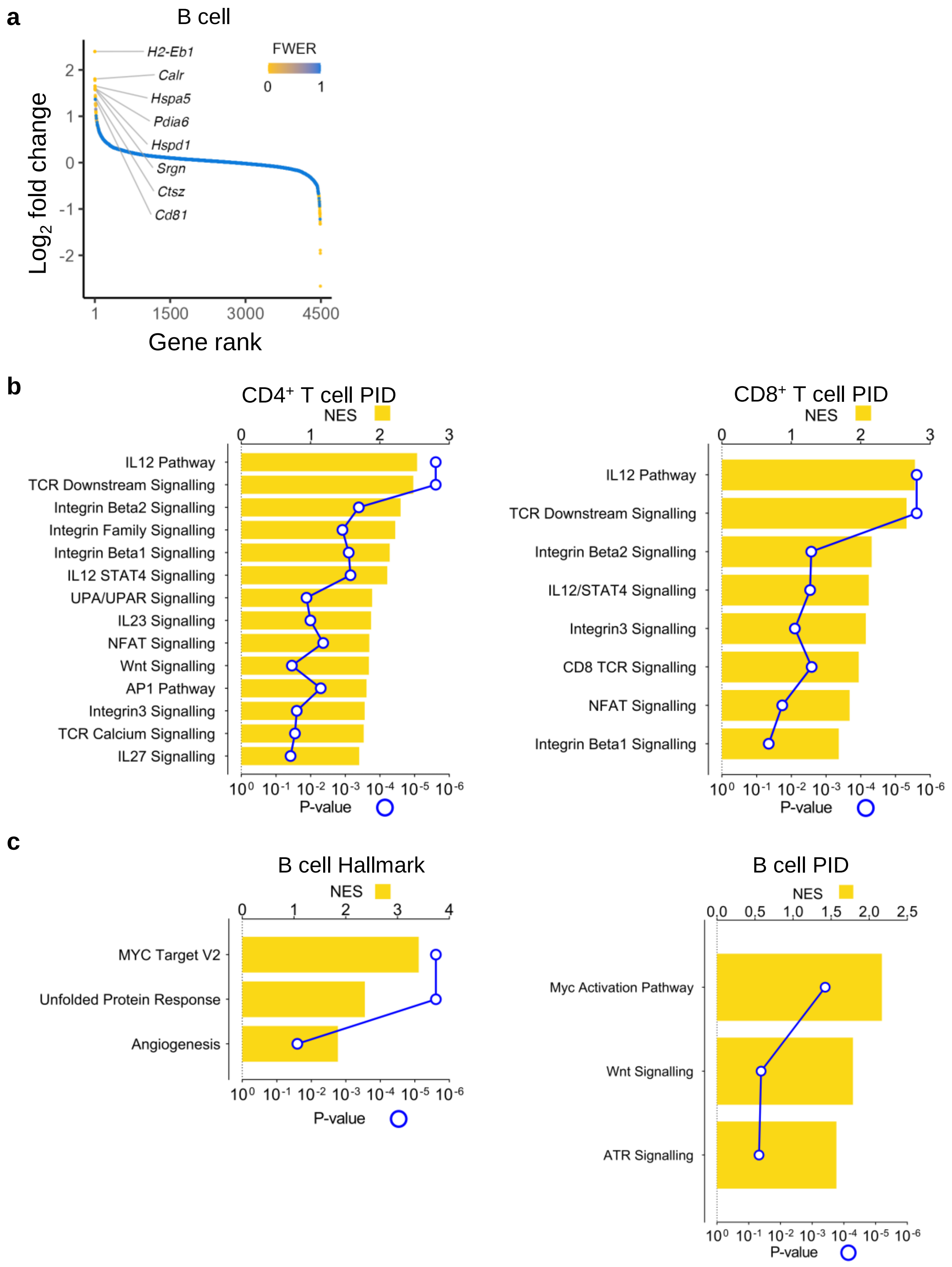

Supplementary Figure 11: Single cell RNA-seq analysis of different immune compartments in CPI-induced colitis

(a) Chair plots showing differentially expressed transcripts ranked by decreasing log fold change and coloured by estimated FDR across all B cell clusters in Balb/C wildtype female 6-week-old mice with CPI-induced colitis (n=3) vs control Balb/C wildtype female 6-week-old mice (n=3). **(b)** Pathways, identified using GSEA PID dataset, upregulated in across all CD4^{+} and CD8^{+} T cell clusters in Balb/C wildtype female 6-week-old mice with CPI-induced colitis (n=3) vs control Balb/C wildtype female 6-week-old mice (n=3). **(c)** Pathways, identified using GSEA Hallmark, upregulated in across all B cell clusters in Balb/C wildtype female 6-week-old mice with CPI-induced colitis (n=3) vs control Balb/C wildtype female 6-week-old mice (n=3). Pathways, identified using GSEA PID dataset, upregulated in across all B cell clusters in Balb/C wildtype female 6-week-old mice with CPI-induced colitis (n=3) vs control Balb/C wildtype female 6-week-old mice (n=3).

Supplementary Figure 12

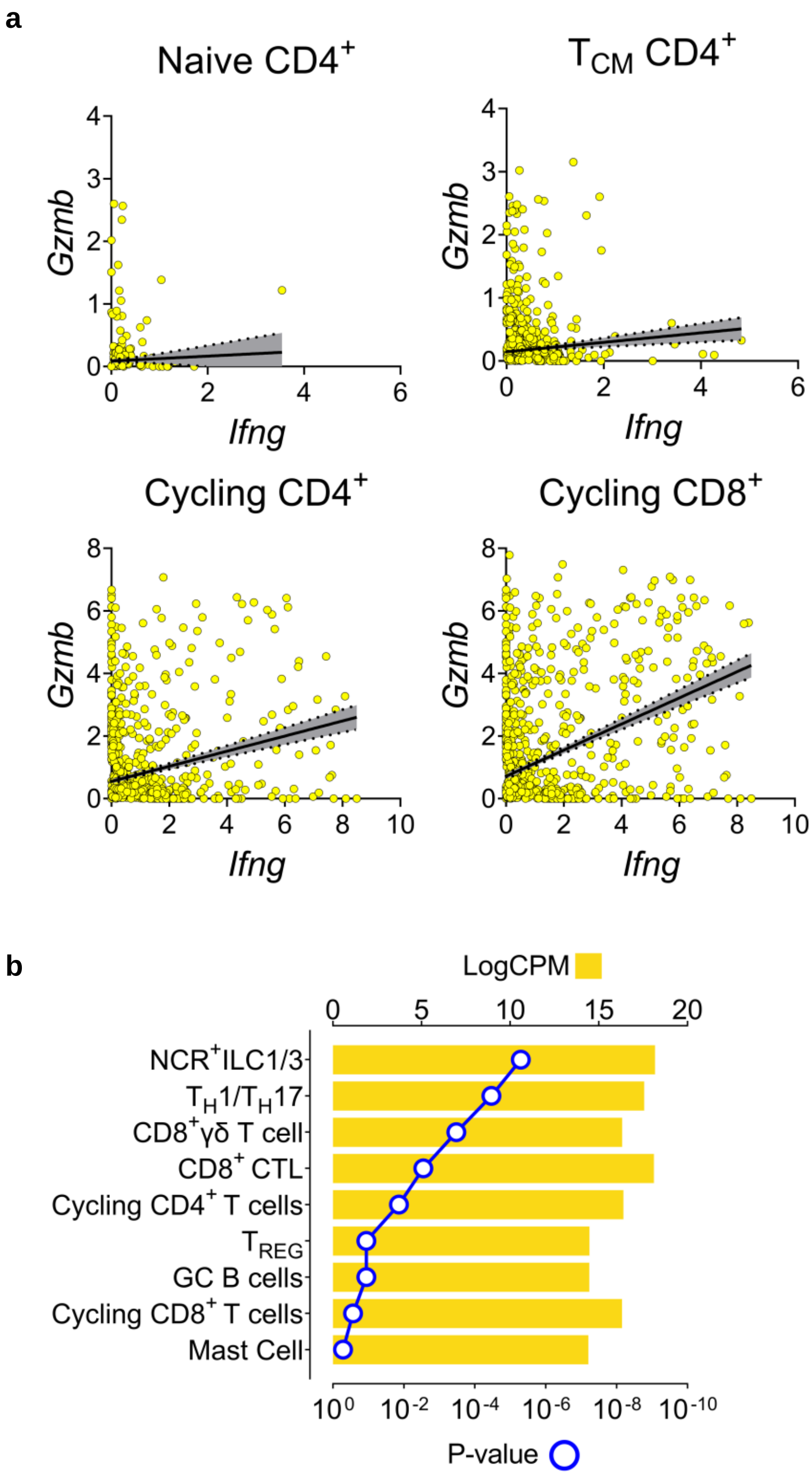

Supplementary Figure 12: Co-expression of granzyme B and IFNγ between individual cells within each cluster
(a) Scatter plot showing each cell and their relative co-expression of *Gzmb* and *Ifng* for naïve CD4$^+$ T cells, CD4$^+$ TCM, T_H1/T_H17 cells, cycling CD4+ T cells and cycling CD8$^+$ T cells. (b) Bar graph showing the relative differential abundance of *Gzmb*$^+$ *Ifng*$^+$ cells in each population.

Supplementary Figure 13

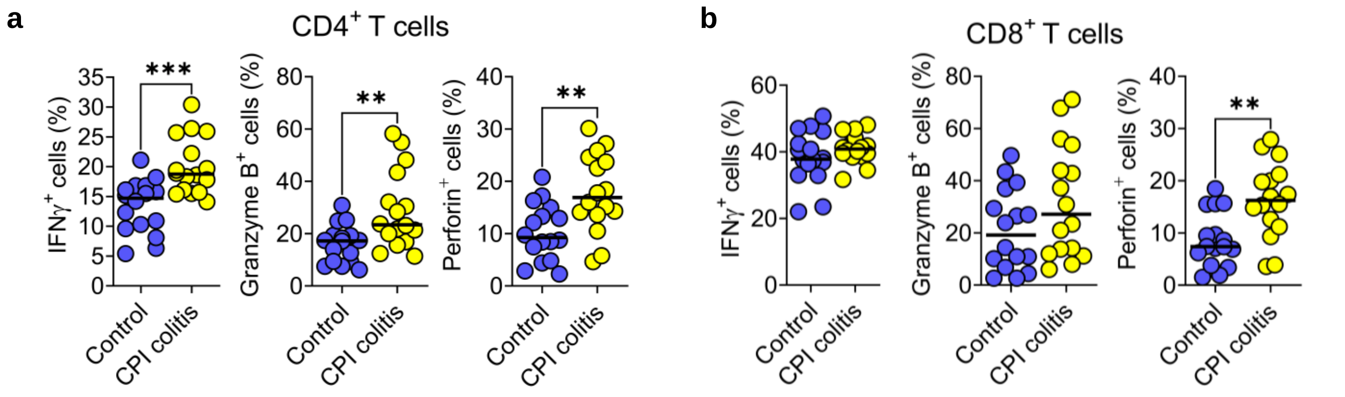

Supplementary Figure 13: Cytotoxic profile by flow cytometry of CD4^{+} and CD8^{+} T-cells

(a) Dot plots from flow cytometry data showing the proportions of IFNγ, granzyme B and perforin producing CD4^{+} and **(b)** CD8^{+} T cells in Balb/C wildtype female 6-week-old mice (n=16) and in Balb/C wildtype female 6-week-old mice treated with FMT and CPI (n=16).

Supplementary Figure 14

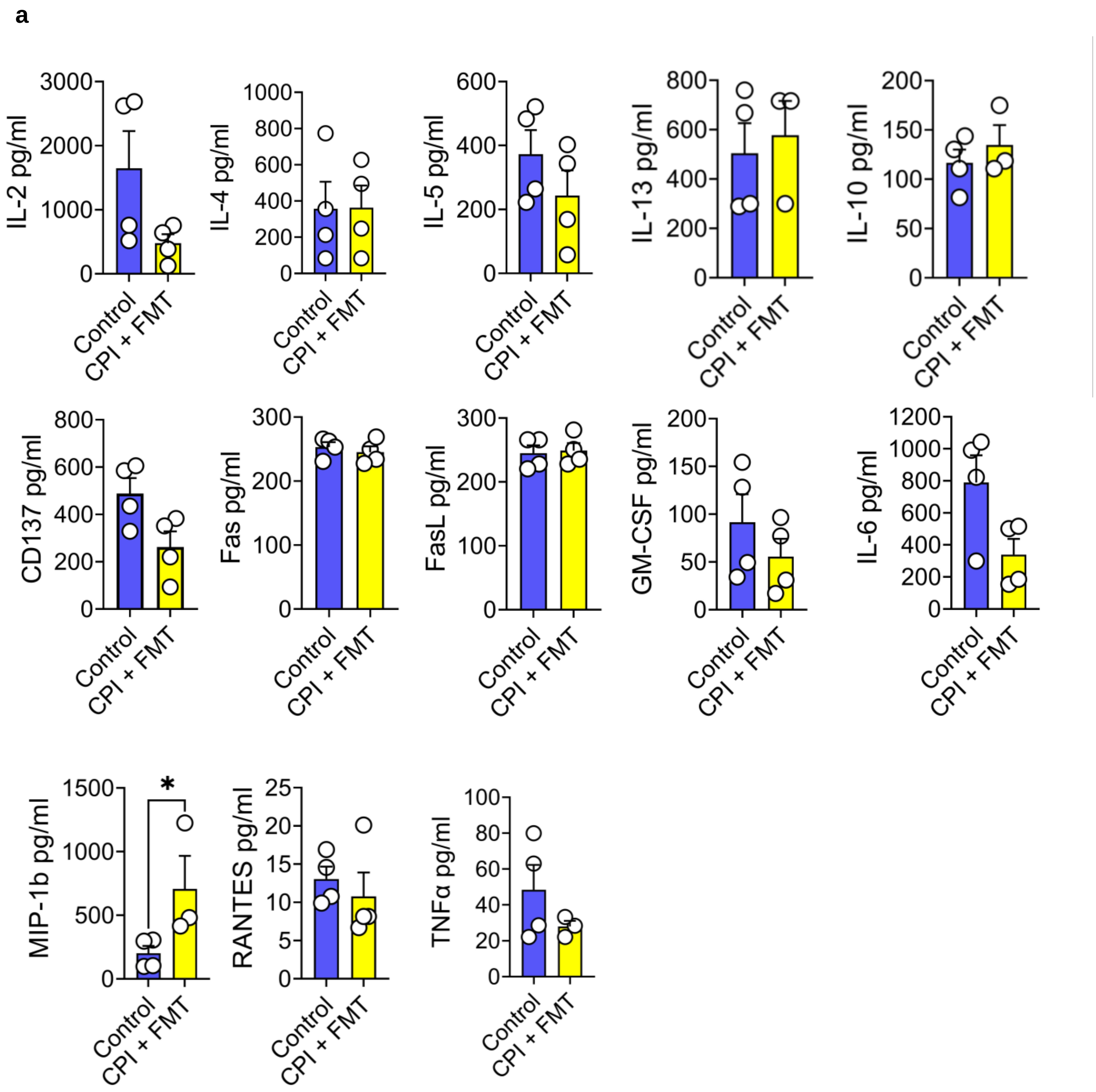

Supplementary Figure 14: Cytotoxic cytokine profile analysis by Luminex assay of cultured colonic lamina propria cells

(a) Concentrations of cytotoxic proteins were determined via Luminex of supernatants from cultured colonic lamina propria cells at 2x10^6/ml for 24 hours with plate bound anti-CD3 (2µg/ml) for untreated Balb/C wildtype female 6-week-old mice (Control: n=4) and Balb/C wildtype female 6-week-old mice treated with CPI-colitis model (CPI+FMT: n=4). Mean and standard deviation are shown. * P<0.05 two-sided Mann-Whitney U T test performed.

Supplementary Figure 15

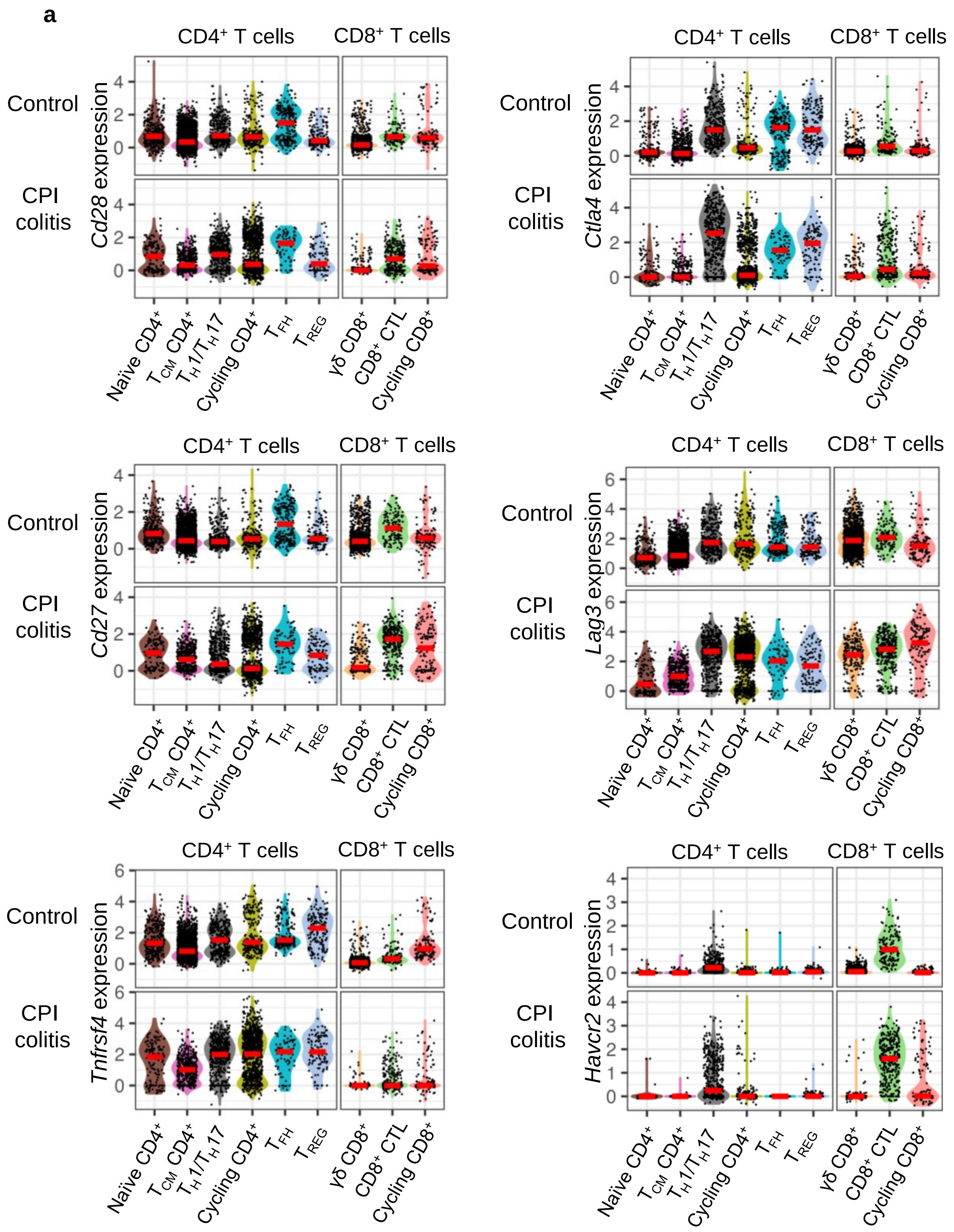

Supplementary Figure 15: Violin plots showing expression of different checkpoint genes in the CD4+ and CD8+ T cell clusters from the single cell RNA-seq dataset

(a) Violin plots showing the expression levels of checkpoint genes across CD4+ and CD8+ T cell clusters in Balb/C wildtype female 6-week-old mice with CPI-induced colitis.

Supplementary Figure 16

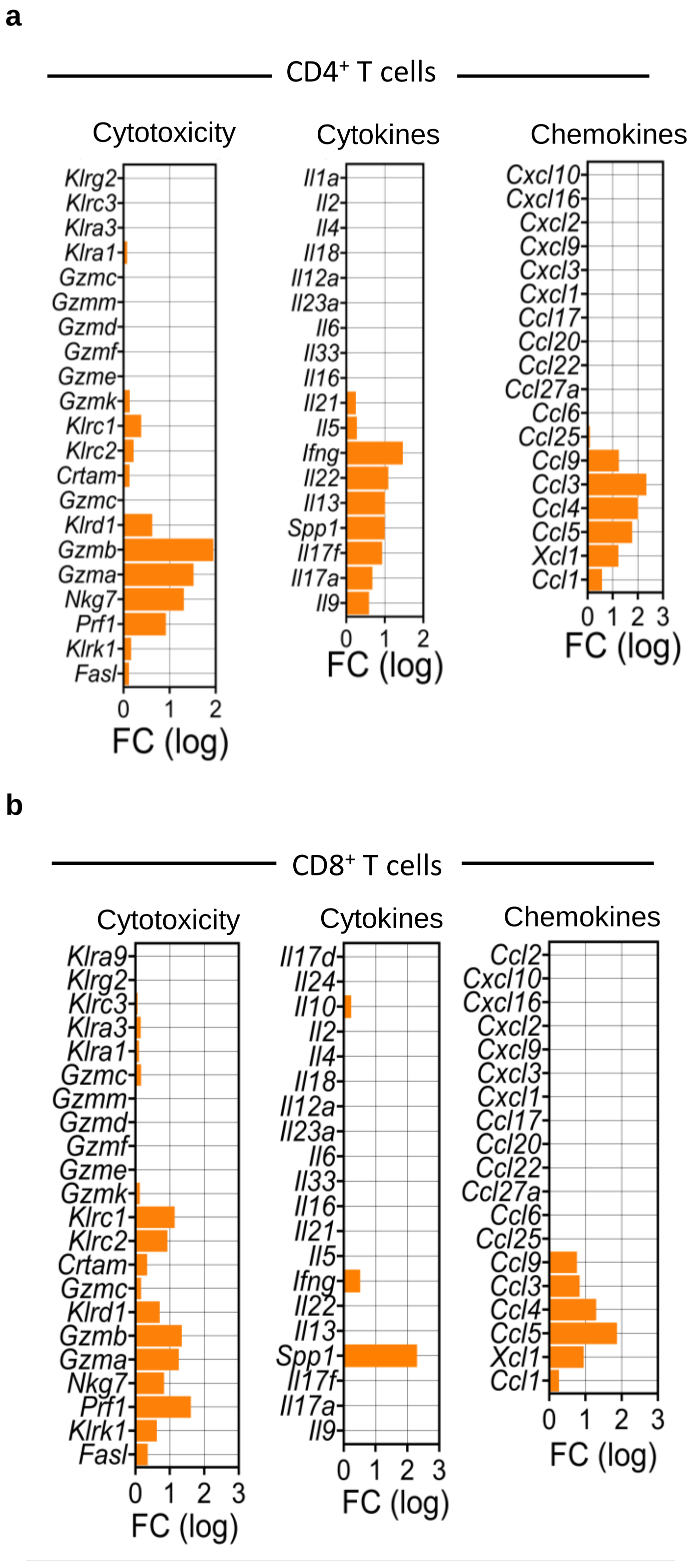

Supplementary Figure 16: IFNγ+ CD4+ and CD8+ T cells have increased expression of cytotoxic genes

(a) Bar graph of expression levels of significantly expressed cytotoxic, cytokines and chemokines genes in IFNγ+ CD4+ T cell clusters and **(b)** CD8+ T cell clusters compared to IFNγ- clusters.

Supplementary Figure 17

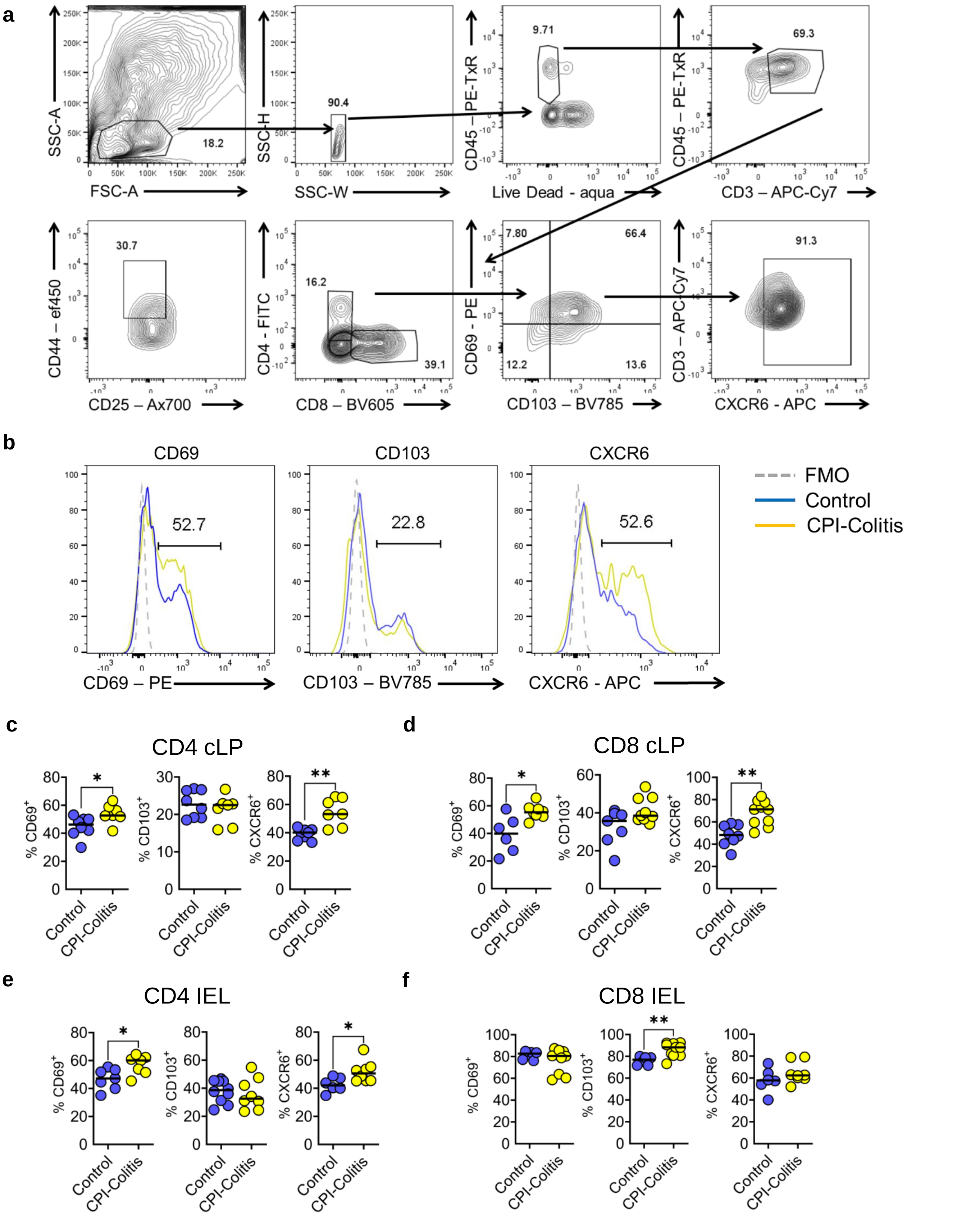

Supplementary Figure 17: Tissue resident memory T cells are expanded in mice treated with CPI-induced colitis. **(a)** Representative gating strategy to identify tissue resident memory $CD4^+$ and $CD8^+$ T cells in the intra-epithelial layer and colonic lamina propria. **(b-f)** Representative histograms and summary dot plots showing CD69, CD103 and CXCR6 expressing cells from $CD45^+$ $CD3^+$ $CD44^+$ $CD25^-$ gated cells for either $CD4^+$ or $CD8^+$ T cells from cLP and intra-epithelial layer. * P<0.05 ** P< 0.01 two sided Mann-Whitney U test.

Supplementary Figure 18

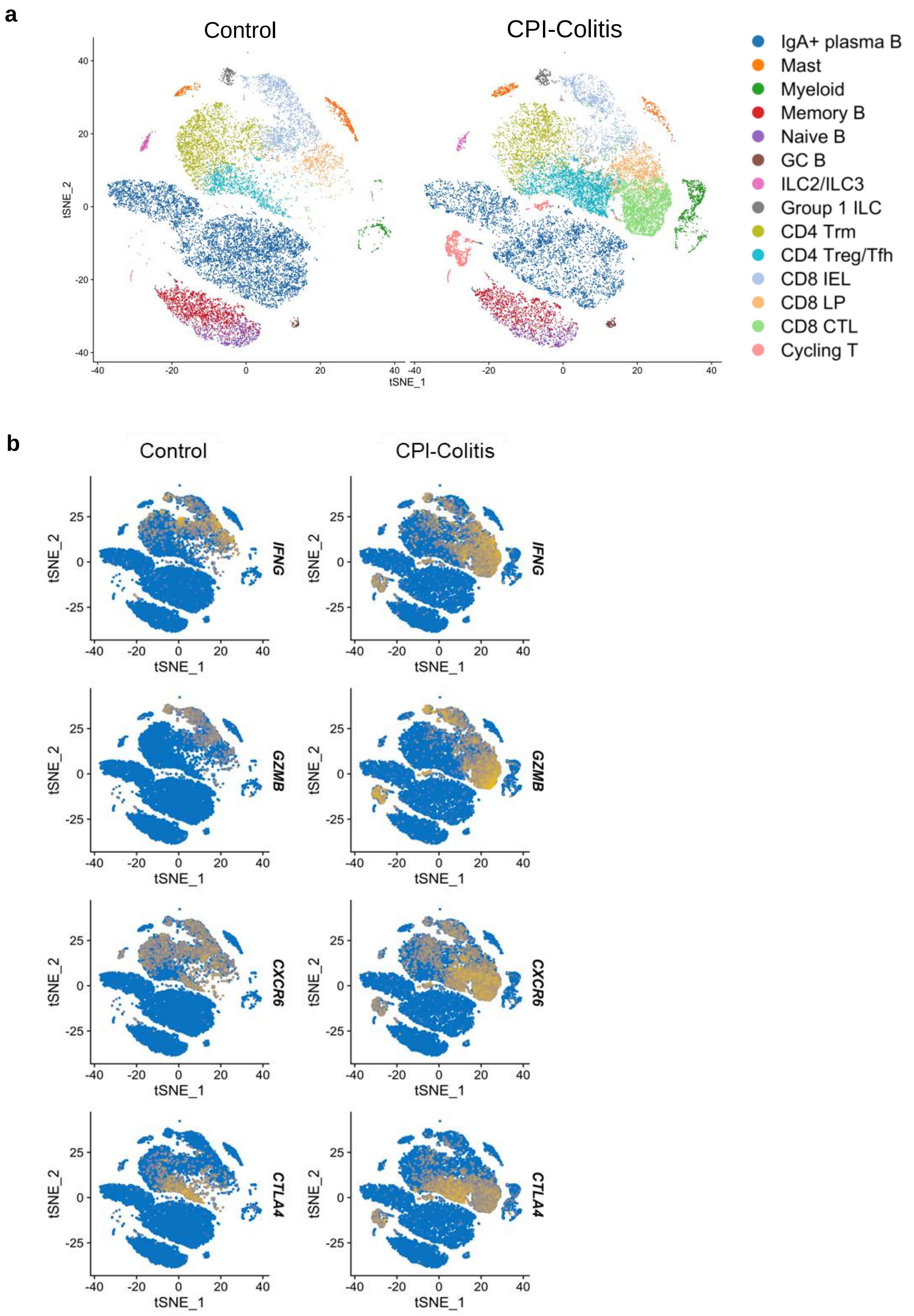

Supplementary Figure 18: Analysis of a human single cell RNA-seq database shows expansion in polyfunctional CXCR6⁺ T cells in patients with CPI-induced colitis

(a) Summary t-SNE plot showing the increased abundance of T cells in patients with CPI-induced colitis and **(b)** *IFNG*, *GZMB*, *CXCR6* and *CTLA4* expressing cells in CPI-induced colitis is increased within these T cell clusters.

Supplementary Figure 19

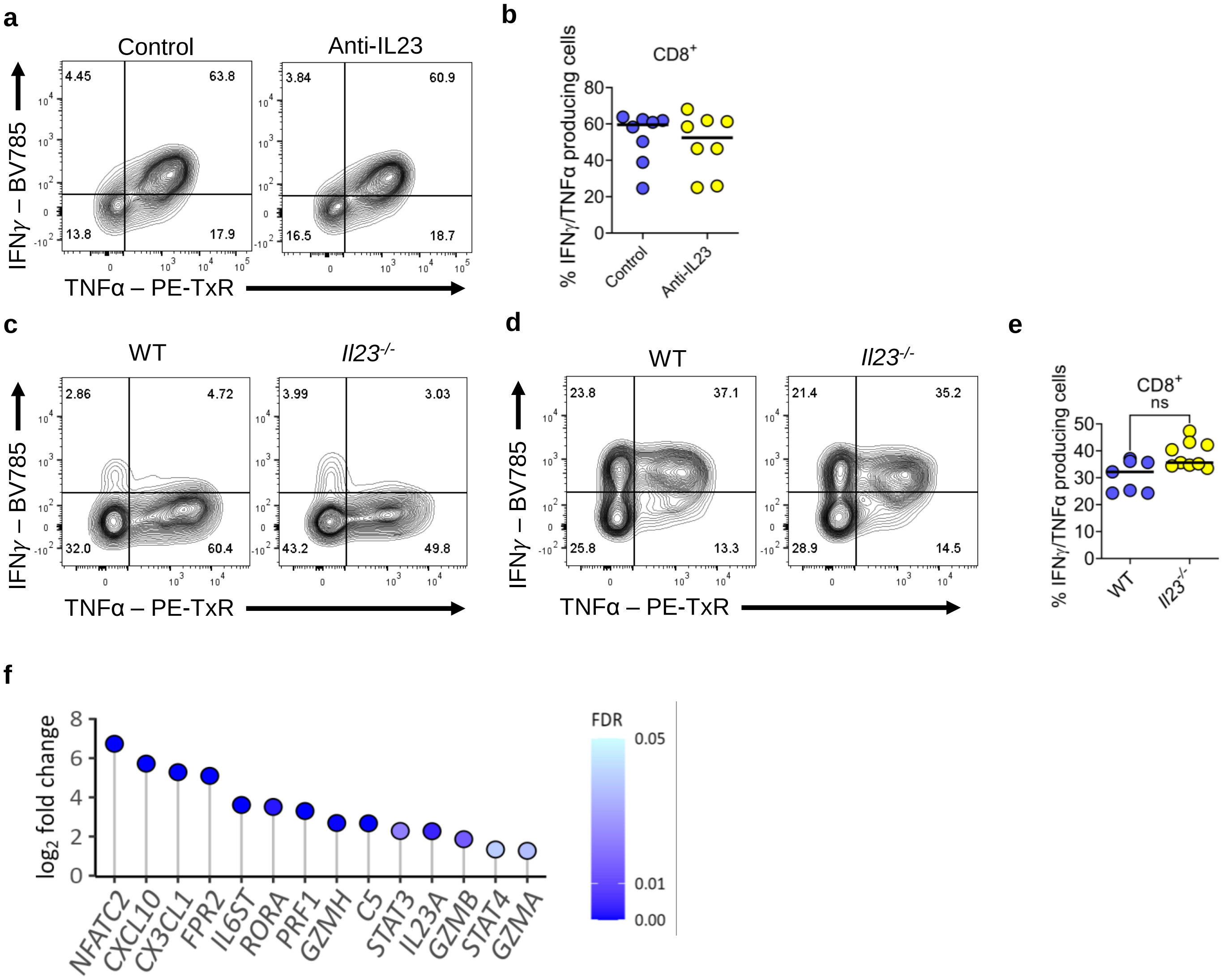

Supplementary Figure 19: CD8⁺ T cells remain unaffected by blockade or deletion of IL23

(a) Representative flow cytometry plot and **(b)** percentage of IFNγ⁺/TNFα⁺ CD8⁺ T cells from CPI-colitis mice treated with an isotype control (n=7) or an IL-23 blocking antibody (n=8). **(c)** Representative flow cytometry plot of IFNγ⁺/TNFα⁺ CD4⁺ T cells from CPI-colitis. **(d)** Representative flow cytometry plot and **(e)** percentage of IFNγ⁺/TNFα⁺ CD8⁺ T cells from CPI-colitis treated wildtype mice (n=8) or *Il23*$^{-/-}$ mice (n=12). **(f)** Significant gene expression changes in biopsies of CPI-colitis patients (n=4) compared to healthy controls (n=4) for selected cytokines, cytokine receptors, transcription factors and enzymes.

Supplementary Table 1: Log2 fold change of significantly (FDR<0.05) affected IFNγ-responsive genes across both monotherapy groups and combination treated mice in comparison to untreated wildtype mice

Gene	Control	Anti-PD1	Anti-CTLA4	Combo
Cxcl9	0	2.442055	2.985742	3
Ccl5	0	1.975357	2.239717	2.830492
Itgb7	0	2.211525	2.162763	2.7647
Ido1	0	0	3	2.72178
Il2rb	0	1.776591	1.791398	2.490711
Lcp2	0	1.731431	1.894874	2.454049
Cd74	0	1.655107	2.080909	2.325956
Il10ra	0	1.714783	2.142492	2.286464
H2-DMa	0	1.598593	2.133772	2.228742
Irf5	0	1.6683	1.795402	2.220111
Nlrc5	0	1.539597	1.996589	2.218237
Cd40	0	1.629376	1.566275	2.195199
Irf4	0	1.861109	1.534996	2.167964
H2-Eb1	0	1.422383	1.65802	2.162015
H2-Q7	0	1.969095	2.332177	2.083436
Ciita	0	1.378337	1.467853	2.05399
Mx2	0	1.698999	2.224597	2.023242
Gpr18	0	1.367034	1.262798	2.006762
Tnfaip2	0	1.557219	2.205365	1.980085
Csf2rb2	0	1.518564	1.670252	1.951819
Oas2	0	1.715371	2.555471	1.945127
Gbp8	0	1.121293	1.438611	1.91782
Cd69	0	0	0	1.786788
Bank1	0	0	0	1.721824
Gbp4	0	1.23357	1.629009	1.696665
Slamf7	0	1.175761	0	1.693258
Marchf1	0	0.858574	0	1.634277
Ifi44l	0	1.373994	1.151318	1.626029
Vcam1	0	1.134351	1.493942	1.617672
Socs3	0	1.344647	1.6042	1.570055
St8sia4	0	1.052078	0.908556	1.544892
C1s1	0	1.197122	1.485276	1.530626
Lysmd2	0	1.320063	1.628191	1.495499
Ifnar2	0	1.258924	1.350035	1.493078
Ptgs2	0	0.800654	1.167666	1.464929
Icam1	0	1.141279	1.206719	1.461921
H2-Aa	0	0.680802	1.071101	1.430381
Cmklr1	0	1.403111	1.578389	1.369303
Eif4e3	0	0.947876	1.221351	1.347406
Fcgr1	0	1.288068	1.402483	1.324412
Serping1	0	1.08401	1.327244	1.286541

Ly6e	0	0.963686	1.204624	1.273607
Cd86	0	1.053421	0.956802	1.265276
Vamp5	0	0.885774	0.882339	1.180152
Pde4b	0	0.734374	1.057771	1.169805
Lats2	0	0.786831	0.873933	1.150457
Bst2	0	0.789683	0.859102	1.079892
Gbp9	0	0.694197	1.005837	1.048438
Tap1	0	0.805159	1.330043	1.039837
H2-M3	0	0.603852	0.71959	1.036375
Ptpn6	0	0.619887	0.560505	1.034243
Stat1	0	0.735057	0.894371	1.02561
Gbp3	0	0	1.15745	1.024284
Tnfaip6	0	0.990353	1.452198	1.014267
Psmb8	0	0	1.014526	0.990346
Sp110	0	0.611828	0.689052	0.96737
Socs1	0	0.827171	1.128756	0.957038
St3gal5	0	0.904739	1.161123	0.939227
Ifitm3	0	0.579278	0.917364	0.901279
Zbp1	0	0	1.592997	0.88111
Psmb9	0	0	0.819521	0.86655
Pim1	0	0.572687	0.694194	0.864806
Rtp4	0	0	1.016945	0.848274
Ifit2	0	0	0.622656	0.815188
Cfh	0	0.632511	0.846689	0.798726
Trafd1	0	0.407234	1.045817	0.787452
Cd274	0	0	0.832534	0.765269
Irf8	0	0.698653	0.820393	0.758209
Irf9	0	0.55864	0.912911	0.738815
Mthfd2	0	0.900396	0.830027	0.701888
Xaf1	0	0	0.736264	0.667996
Il18bp	0	0	0.796051	0.655584
Ifitm2	0	0.622613	0	0.649738
Il4ra	0	0.676785	0.604983	0.632766
Sspn	0	0	0.708794	0.575189
Slc25a28	0	0.539421	0.794926	0.56711
Ifi35	0	0.438056	0.495371	0.540764
Ube2l6	0	0	0	0.525718
Ripk2	0	0.438517	0	0.48139
B2m	0	0	0	0.43537
Irf2	0	0	0.465967	0.401178
Ptpn1	0	0.303799	0.476127	0.379769
Pml	0	0	0.425424	0.374318
Nod1	0	0.366282	0.377606	0.369821
H2-D1	0	0	0.347983	0.341965
Ifi30	0	0	0	0.261304
Oas3	0	0	2.519131	0
Ccl2	0	1.557485	2.041919	0

H2-Q10	0	0	1.379032	0
Ifi44	0	0	1.270745	0
Ifit3	0	0	1.139178	0
Upp1	0	0	1.081647	0
Rsad2	0	0	1.011306	0
Isg15	0	0	0.999278	0
Nup93	0	0	0.766476	0
Lgals3bp	0	0	0.603341	0
Stat2	0	0	0.416621	0
Rbck1	0	0.256208	0.244616	0
Nfkb1	0	0.298916	0	0
Ddx60	0	-1.22004	0	0
Txnip	0	-0.4355	0	0
Jak2	0	-0.55445	0	0
Sri	0	0	-0.29557	0
Vamp8	0	0	-0.43038	0
Parp14	0	-0.79084	-0.47945	0
Samd9l	0	-0.66708	-0.59528	0
Casp1	0	-0.31386	-0.43583	-0.3154
Casp7	0	-0.35367	-0.32339	-0.33556
Sod2	0	-0.29084	-0.27979	-0.35506
Btg1	0	-0.50262	-0.41273	-0.36821
Nampt	0	-0.30313	0	-0.39128
Znfx1	0	-0.4619	0	-0.39281
Cd38	0	-0.3484	-0.46863	-0.4186
Ripk1	0	-0.43102	-0.42692	-0.46764
Auts2	0	0	-0.77888	-0.47469
Samhd1	0	-0.73971	-0.94242	-0.47988
Cdkn1a	0	-0.39541	-0.79134	-0.50879
Mvp	0	0	0	-0.52468
Helz2	0	-0.81042	-0.65778	-0.54883
Arl4a	0	-0.65968	-0.84888	-0.5546
Pfkp	0	-0.44155	0	-0.56591
Sppl2a	0	-0.60778	-0.71175	-0.57822
Tdrd7	0	-0.40045	-0.36701	-0.58428
Trim25	0	-0.78522	-0.6255	-0.62084
Rapgef6	0	-0.8882	-0.96949	-0.62422
Ncoa3	0	-0.83523	-0.49178	-0.63031
Ifih1	0	-0.8419	-0.62668	-0.67715
Eif2ak2	0	-0.73403	-0.62327	-0.69863
Tnfaip3	0	-0.7213	-0.92811	-0.7124
Rnf213	0	-0.94177	-0.83936	-0.71497
Adar	0	-0.64376	-0.72662	-0.7579
Mettl7b	0	0	-0.85728	-0.83031

Supplementary Table 2: Upstream regulators of CD4^{+} IFNγ^{+} T-cells in CPI colitis vs CD4^{+} IFNγ^{+} T-cells in control mice

Master Regulator	Molecule Type	Predicted Activation	Activation z-score	p-value of overlap
parathyroid hormone-related protein	biologic drug	Activated	4.003	3.55E-17
prosaptide d1	biologic drug	Activated	3.25	5.93E-21
afamelanotide	biologic drug	Activated	3.048	8.83E-20
lypressin	biologic drug	Activated	2.828	4.44E-23
filgrastim	biologic drug	Activated	2.777	1.03E-10
GLP-1 (7-36) amide	biologic drug	Activated	2.683	3.19E-27
filgrastim	biologic drug	Activated	2.682	1.55E-26
blinatumomab	biologic drug	Activated	2.236	0.00000139
enterotoxin B	biologic drug	Activated	2.183	8.23E-13
amino acids	chemical - endogenous mammalian	Activated	4.841	1.14E-39
2'-fucosyllactose	chemical - endogenous mammalian	Activated	3.719	2.61E-29
leukotriene D4	chemical - endogenous mammalian	Activated	3.181	3.23E-26
1-octanol	chemical - endogenous mammalian	Activated	2.955	5.03E-72
copper	chemical - endogenous mammalian	Activated	2.573	1.23E-39
heparan sulfate	chemical - endogenous mammalian	Activated	2.55	1.06E-26
histamine	chemical - endogenous mammalian	Activated	2.496	1.33E-09
diadenosine triphosphate	chemical - endogenous mammalian	Activated	2.449	1.55E-15
24R,25-dihydroxyvitamin D3	chemical - endogenous mammalian	Activated	2.321	1.69E-19
leukotriene D4	chemical - endogenous mammalian	Activated	2.111	0.00000071
phosphate	chemical - endogenous mammalian	Activated	2.086	1.9E-22

leukotriene E4	chemical - endogenous mammalian	Activated	2	0.0000245
E. coli B5 lipopolysaccharide	chemical - endogenous non-mammalian	Activated	2.897	6.35E-21
archazolid B	chemical - endogenous non-mammalian	Activated	2.885	1.89E-18
proteoglycan	chemical - other	Activated	4.885	3.68E-27
cardiotoxin	chemical - other	Activated	2	3.94E-09
lobeline	chemical drug	Activated	6.557	2.15E-56
SGI 1776	chemical drug	Activated	5.425	2.65E-57
CB-5083	chemical drug	Activated	5.248	5.88E-57
5-fluorouracil	chemical drug	Activated	4.636	1.38E-27
ST1926	chemical drug	Activated	4.271	1.19E-12
beta-hydroxy simvastatin acid	chemical drug	Activated	4.243	7.8E-62
CD 437	chemical drug	Activated	3.773	2.96E-13
sirolimus	chemical drug	Activated	3.545	1.08E-44
nomifensine	chemical drug	Activated	3.415	4.64E-58
verteporfin	chemical drug	Activated	3.179	1.11E-57
cysteamine	chemical drug	Activated	2.994	1.35E-23
highly active antiretroviral therapy	chemical drug	Activated	2.985	9.4E-14
(-)-isoproterenol	chemical drug	Activated	2.907	1.47E-21
amrinone	chemical drug	Activated	2.889	2.04E-23
levocabastine	chemical drug	Activated	2.689	3.6E-19
dobutamine	chemical drug	Activated	2.546	9.32E-18
xylometazoline	chemical drug	Activated	2.54	3.64E-31
SN-38	chemical drug	Activated	2.331	4.17E-27
ciprofloxacin	chemical drug	Activated	2.263	1.98E-16
AGN194204	chemical drug	Activated	2.16	1.44E-22
PLX5622	chemical drug	Activated	2.065	1.73E-13
benzo[1,3]dioxol-5-yl-[2-(4-fluoro-phenylamino)-4-methyl-thiazol-5-yl]-methanone	chemical reagent	Activated	6.557	1.84E-56
4OH-GTS-21	chemical reagent	Activated	6.557	1.84E-56
epibatidine	chemical reagent	Activated	6.123	1.77E-57
CGP 57380	chemical reagent	Activated	5.343	3.45E-61
D-Pen-2,5-enkephalin	chemical reagent	Activated	3.487	4.77E-26
SC144	chemical reagent	Activated	3.252	4.28E-57
dioctanoylphosphatidic acid	chemical reagent	Activated	2.964	4.02E-25
N6-cyclohexyladenosine	chemical reagent	Activated	2.61	4.81E-20
alpha-amino-3-hydroxy-5-methyl-4-isoxazolepropionic acid	chemical reagent	Activated	2.562	2.67E-19

ionomycin	chemical reagent	Activated	2.53	7.66E-22
MALP-2s	chemical reagent	Activated	2.449	0.0000392
galactosylceramide-alpha	chemical reagent	Activated	2.324	4.26E-11
trinitrobenzenesulfonic acid	chemical reagent	Activated	2.324	6.73E-11
compound 48/80	chemical reagent	Activated	2.321	1.95E-22
silver	chemical reagent	Activated	2.287	3.33E-14
CpG ODN D-19	chemical reagent	Activated	2.101	3.17E-22
NSC-295642	chemical reagent	Activated	2.023	1.2E-17
NSC-150117	chemical reagent	Activated	2.023	1.2E-17
Ige	complex	Activated	4.749	1.3E-32
Ige	complex	Activated	4.715	5.34E-23
Stat1 dimer	complex	Activated	4.372	1.79E-71
HLA-DQ	complex	Activated	3.775	1.39E-24
IL6 receptor	complex	Activated	3.442	1.69E-26
Fcer1	complex	Activated	3.153	9.53E-14
IL23	complex	Activated	2.887	6.28E-10
MHC Class I (complex)	complex	Activated	2.673	1.35E-21
IL17a dimer	complex	Activated	2.646	1.77E-08
IL23	complex	Activated	2.578	6.75E-30
IL-17f dimer	complex	Activated	2.449	1.13E-08
Gm-Csf Receptor	complex	Activated	2.325	3.35E-24
E2f-Dp1	complex	Activated	2.309	1E-10
NFAT (complex)	complex	Activated	2.021	1.42E-22
Glycoprotein 1B	complex	Activated	2.021	4.55E-16
IL12 (complex)	complex	Activated	2	2.8E-20
IL2	cytokine	Activated	5.047	1.65E-31
IL15	cytokine	Activated	3.775	1.16E-30
IL33	cytokine	Activated	3.656	4.51E-13
IL15	cytokine	Activated	3.382	5.18E-34
IL5	cytokine	Activated	2.949	4.15E-17
IFNL3	cytokine	Activated	2.714	7.4E-26
IL7	cytokine	Activated	2.667	3.94E-23
IL23A	cytokine	Activated	2.578	2.02E-31
IL4	cytokine	Activated	2.429	1.24E-20
IL20	cytokine	Activated	2.405	1.12E-21
IL25	cytokine	Activated	2.333	0.000000808
CCL11	cytokine	Activated	2.278	6.26E-25
MYDGF	cytokine	Activated	2.121	2.16E-10
IL18	cytokine	Activated	2	3.94E-23
IFNL1	cytokine	Activated	2	1.97E-25
FBXL14	enzyme	Activated	5.167	8.36E-56
NDST1	enzyme	Activated	3.732	1.01E-23
GNG3	enzyme	Activated	3.333	1.12E-15
PLA2G5	enzyme	Activated	3.255	9.88E-24
GPNMB	enzyme	Activated	3.201	4.55E-21
ATE1	enzyme	Activated	3.201	6.98E-18
ANXA1	enzyme	Activated	2.969	2.09E-24

CD38	enzyme	Activated	2.785	1.8E-12
CCS	enzyme	Activated	2.777	2.48E-15
PLCG2	enzyme	Activated	2.753	1.22E-26
PPIF	enzyme	Activated	2.673	1.52E-08
TKT	enzyme	Activated	2.673	7.59E-22
RC3H1	enzyme	Activated	2.425	1.31E-25
RNASE2	enzyme	Activated	2.333	1.9E-09
PDE6G	enzyme	Activated	2.287	3.88E-14
PLCD4	enzyme	Activated	2.252	3.18E-21
GNAI3	enzyme	Activated	2.251	5.45E-23
LTC4S	enzyme	Activated	2.236	0.00000024
RAP1B	enzyme	Activated	2.236	0.00000024
OTUD5	enzyme	Activated	2.138	0.000000028
GNAI3	enzyme	Activated	2.121	1.04E-09
FAAH	enzyme	Activated	2.121	0.000000718
UBE3C	enzyme	Activated	2.121	1.14E-13
METTL3	enzyme	Activated	2.065	1.56E-10
CRHR1	G-protein coupled receptor	Activated	3.58	3.43E-26
OPRM1	G-protein coupled receptor	Activated	3.392	1.81E-30
ADRA2C	G-protein coupled receptor	Activated	3.25	5.93E-21
GNRHR	G-protein coupled receptor	Activated	3.15	5.36E-19
CCR2	G-protein coupled receptor	Activated	3.087	7.69E-30
ADRA1A	G-protein coupled receptor	Activated	3	9.13E-29
ADRA2B	G-protein coupled receptor	Activated	2.954	2.13E-21
TRHR	G-protein coupled receptor	Activated	2.944	1.19E-24
ADRA1B	G-protein coupled receptor	Activated	2.626	1.2E-32
ADORA3	G-protein coupled receptor	Activated	2.6	3.84E-28
LTB4R2	G-protein coupled receptor	Activated	2.502	1.18E-27
ADORA3	G-protein coupled receptor	Activated	2.333	1.18E-12
HRH1	G-protein coupled receptor	Activated	2	0.0000706
Ap2	group	Activated	6.158	4.98E-69
TLR7/8	group	Activated	3.989	1.88E-28
Eotaxin	group	Activated	3.713	8.54E-29
apyrase	group	Activated	3.615	3.65E-56
Presenilin	group	Activated	3.111	7.62E-16
G protein alpha	group	Activated	3.109	1.77E-24

MIR124	group	Activated	3.073	1.19E-59
STAT5a/b	group	Activated	2.828	1.7E-10
PI3Kγ	group	Activated	2.708	5.31E-21
BET	group	Activated	2.619	1.13E-25
Profilin	group	Activated	2.4	1.38E-12
Fcer	group	Activated	2.309	5.01E-11
Nfatc	group	Activated	2.197	8E-16
STAT	group	Activated	2.117	2.92E-26
antigen	group	Activated	2	0.0000245
TGFB1	growth factor	Activated	4.619	4.68E-27
EDN2	growth factor	Activated	2.287	3.33E-14
KCND2	ion channel	Activated	3.536	1.82E-26
ILK	kinase	Activated	6.596	9.04E-34
BRAF	kinase	Activated	3.967	1.79E-23
PINK1	kinase	Activated	3.447	1.73E-20
TEK	kinase	Activated	3.317	1.19E-28
BTK	kinase	Activated	3.153	3.25E-10
AGK	kinase	Activated	2.964	9.66E-24
MOS	kinase	Activated	2.8	4.79E-24
NTRK2	kinase	Activated	2.762	1.27E-26
FGR	kinase	Activated	2.373	1.2E-24
CARD11	kinase	Activated	2.333	5.94E-11
STYK1	kinase	Activated	2.25	1.97E-22
NEK6	kinase	Activated	2.177	9.9E-23
SKAP1	kinase	Activated	2.064	8.73E-16
miR-125b-5p (and other miRNAs w/seed CCCUGAG)	mature microRNA	Activated	2.889	1.8E-57
miR-542-3p (miRNAs w/seed GUGACAG)	mature microRNA	Activated	2	0.000159
mir-24	microRNA	Activated	3.507	6.31E-19
mir-191	microRNA	Activated	2.524	6.42E-11
RICTOR	other	Activated	6.719	2.21E-33
AMBRA1	other	Activated	5.879	1.04E-60
CUL4B	other	Activated	5.691	5.12E-59
CDR2	other	Activated	5.683	2.34E-55
Ifi202b	other	Activated	5.06	1.08E-58
IGHE	other	Activated	3.983	1.44E-22
SDC2	other	Activated	3.637	5.61E-24
MFGE8	other	Activated	3.578	5.05E-23
C5	other	Activated	3.53	4.45E-30
UCN2	other	Activated	3.255	5.8E-21
RBM8A	other	Activated	3.17	1.19E-25
ANK2	other	Activated	3.15	4.55E-20
RICTOR	other	Activated	3.103	6.21E-41
Prl3d1 (includes others)	other	Activated	3	5.92E-23
TRG	other	Activated	2.714	2.67E-15
IAPP	other	Activated	2.712	5.63E-22

SH2D5	other	Activated	2.673	7.59E-22
ICOSLG/LOC102723996	other	Activated	2.646	0.000000149
IFNL2	other	Activated	2.574	2.31E-27
MSLN	other	Activated	2.462	9.47E-24
MUC16	other	Activated	2.405	1.97E-21
PTGFRN	other	Activated	2.287	3.33E-14
CNN3	other	Activated	2.287	3.33E-14
COL5A3	other	Activated	2.287	3.33E-14
NMU	other	Activated	2.236	0.0000496
RASSF5	other	Activated	2.236	0.00205
NPHP1	other	Activated	2.188	2.41E-16
MS4A1	other	Activated	2.183	1.19E-10
TNFAIP8	other	Activated	2.111	6.15E-08
GP9	other	Activated	2.021	4.55E-16
MOG	other	Activated	2	3.19E-17
RTKN	other	Activated	2	0.000159
METRNL	other	Activated	2	0.000159
USP10	peptidase	Activated	4.566	1.09E-55
KLK5	peptidase	Activated	4.15	1.48E-32
MMP14	peptidase	Activated	3.817	9.34E-29
MMP2	peptidase	Activated	2.858	4.7E-25
MMP17	peptidase	Activated	2.828	1.32E-25
FAP	peptidase	Activated	2.066	3.89E-22
CTDSP1	phosphatase	Activated	5.539	2.6E-55
PTPRD	phosphatase	Activated	3.238	4.91E-57
PTP4A1	phosphatase	Activated	2.654	1.19E-16
PTPN22	phosphatase	Activated	2.449	0.0000146
SP3	transcription regulator	Activated	4.383	4.61E-28
HIF3A	transcription regulator	Activated	4.111	5.41E-29
LMO2	transcription regulator	Activated	4.062	1.1E-58
TRIM24	transcription regulator	Activated	4.041	2.13E-16
ETV6	transcription regulator	Activated	3.709	1.11E-57
BHLHE40	transcription regulator	Activated	3.578	1.33E-10
IRF6	transcription regulator	Activated	3.077	4.98E-23
ZHX2	transcription regulator	Activated	3.048	5.61E-20
STAT6	transcription regulator	Activated	3	7.06E-19
IRF9	transcription regulator	Activated	3	3.29E-24
NKX2-3	transcription regulator	Activated	2.858	2.21E-08
ARNT	transcription regulator	Activated	2.828	5.06E-14
ELF4	transcription regulator	Activated	2.828	0.0000132
ETV5	transcription regulator	Activated	2.828	2.41E-25
TWIST2	transcription regulator	Activated	2.782	1.05E-21
HES5	transcription regulator	Activated	2.734	5.99E-24
ISL1	transcription regulator	Activated	2.689	8.54E-24
STAT3	transcription regulator	Activated	2.562	3.69E-21
TCF4	transcription regulator	Activated	2.557	1.01E-21
BCL3	transcription regulator	Activated	2.496	3.14E-09
WT1	transcription regulator	Activated	2.425	3.53E-22

GATA3	transcription regulator	Activated	2.302	9.54E-37
PRDM1	transcription regulator	Activated	2.263	3.24E-16
MTPN	transcription regulator	Activated	2.224	4.94E-22
TBX21	transcription regulator	Activated	2.132	4.53E-17
NFATC2	transcription regulator	Activated	2.121	1.89E-15
SMAD1	transcription regulator	Activated	2.121	0.000000718
LARP1	translation regulator	Activated	7.488	1.41E-68
IGF2BP2	translation regulator	Activated	2.287	4.51E-14
IGF2R	transmembrane receptor	Activated	4.156	3.82E-32
HMMR	transmembrane receptor	Activated	3.579	1.16E-25
CD69	transmembrane receptor	Activated	3.501	1.24E-18
IL10RA	transmembrane receptor	Activated	3.286	5.83E-12
MPL	transmembrane receptor	Activated	3.273	1.67E-27
IL10RA	transmembrane receptor	Activated	3.222	1.9E-27
Klrk1	transmembrane receptor	Activated	3.13	4.19E-18
CLEC6A	transmembrane receptor	Activated	2.835	3.45E-27
CNTFR	transmembrane receptor	Activated	2.734	5.13E-22
Klra7 (includes others)	transmembrane receptor	Activated	2.646	0.000000712
CD3E	transmembrane receptor	Activated	2.496	8.6E-12
ICOS	transmembrane receptor	Activated	2.496	8.6E-12
CD244	transmembrane receptor	Activated	2.449	0.000000292
CD86	transmembrane receptor	Activated	2.324	1.51E-15
IL13RA1	transmembrane receptor	Activated	2.294	1.53E-23
CD46	transmembrane receptor	Activated	2.25	8.8E-20
CD247	transmembrane receptor	Activated	2.138	1.27E-23
FCER1G	transmembrane receptor	Activated	2.121	0.00000012
TLR4	transmembrane receptor	Activated	2.058	8.23E-15
EDNRA	transmembrane receptor	Activated	2.047	5.18E-24

CD79B	transmembrane receptor	Activated	2.018	1.62E-24
CD19	transmembrane receptor	Activated	2	0.0000245
ULBP2	transmembrane receptor	Activated	2	0.0000706
CRLF2	transmembrane receptor	Activated	2	0.000159
TRPC4AP	transporter	Activated	5.66	3.5E-62
TNNI3	transporter	Activated	2.646	5.34E-10
RBP3	transporter	Activated	2.183	1.11E-09
FLVCR1	transporter	Activated	2	0.0000245
TRPC4AP	transporter	Activated	2	0.0000245

Supplementary Table 3: Flow cytometry antibodies used

Target	Clone	Tag	Isotype	Supplier	Dilution used
Granzyme B	NGZB	PE-Cy7	Rat IgG2b,k	Thermo Fisher	1:50
IFNγ	XMG1.2	BV785	Rat IgG1,k	BioLegend	1:100
IL-17A	17B7	PE-Cy7	Rat IgG2a,k	Thermo Fisher	1:100
Perforin	OMAK-D	APC	Rat IgG2a,k	Thermo Fisher	1:50
TNFa	MP6-XT22	PE/D594	Rat IgG1,k	BioLegend	1:200
CD3	17A2	APC-Cy7	Rat IgG2b,k	BioLegend	1:200
CD4	GK1.5	FITC	Rat IgG2b,k	Thermo Fisher	1:200
CD45	30-F11	PB	Rat IgG2b,k	BioLegend	1:200
CD8a	53-6.7	BV605	Rat IgG2a,k	BioLegend	1:100
Integrin b7	FIB504	BV650	CDF IgG2a,k	BD	1:100
CD11b	M1/70	PE-Cy7	DA/HA IgG2b,k	BD	1:200
CD11c	N418	BV785	Ar Ham IgG	BioLegend	1:50
CD69	H1.2F3	PE	Ar Ham IgG	Thermo Fisher	1:100
CXCR6	DANID2	APC	Rat / IgG2a	Thermo Fisher	1:100
F4/80	BM8	APC-Cy7	Rat IgG2a,k	BioLegend	1:50
FcgRI /or CD64	X54-5/7.1	APC	Ms IgG1,k	BioLegend	1:100
Integrin aE / CD103	2E7	Alexa 700	Ar Ham IgG	Biolegend	1:100
Gr-1	RB6-8C5	FITC	Rat IgG2b,k	BioLegend	1:200
Ly-6C	HK1.4	PEDazzle594	Rat IgG2c	Biolegend	1:100
MHC Class II / I-A/I-E	M5/114.15.2	BV605	Rat IgG2b,k	BioLegend	1:1000
Siglec-F / CD170	S17007L	PE	Rat IgG2a,k	BioLegend	1:100
Live Dead Fixable AQUA	L34966	BV510	N/A	Life Tech	1:400
