# Supplementary material for: Immune checkpoint inhibitor-induced colitis is mediated by polyfunctional lymphocytes and is dependent on an IL23/IFNγ axis
Source: Nat Commun. 2023 Oct 23;14:6719. doi: 10.1038/s41467-023-41798-2 (PMC10593820; doi:10.1038/s41467-023-41798-2)
Supplement: Supplementary file 2 — Reporting Summary [file 41467_2023_41798_MOESM2_ESM.pdf]

Corresponding author(s): DBPR NCOMMS-22-00603B

Last updated by author(s): Aug 31, 2023

Reporting Summary

Nature Portfolio wishes to improve the reproducibility of the work that we publish. This form provides structure for consistency and transparency in reporting. For further information on Nature Portfolio policies, see our Editorial Policies and the Editorial Policy Checklist.

Statistics

For all statistical analyses, confirm that the following items are present in the figure legend, table legend, main text, or Methods section.

n/a	Confirmed	
☐	☒	The exact sample size (*n*) for each experimental group/condition, given as a discrete number and unit of measurement
☐	☒	A statement on whether measurements were taken from distinct samples or whether the same sample was measured repeatedly
☐	☒	The statistical test(s) used AND whether they are one- or two-sided<br>*Only common tests should be described solely by name; describe more complex techniques in the Methods section.*
☐	☒	A description of all covariates tested
☐	☒	A description of any assumptions or corrections, such as tests of normality and adjustment for multiple comparisons
☐	☒	A full description of the statistical parameters including central tendency (e.g. means) or other basic estimates (e.g. regression coefficient) AND variation (e.g. standard deviation) or associated estimates of uncertainty (e.g. confidence intervals)
☐	☒	For null hypothesis testing, the test statistic (e.g. *F*, *t*, *r*) with confidence intervals, effect sizes, degrees of freedom and *P* value noted<br>*Give P values as exact values whenever suitable.*
☒	☐	For Bayesian analysis, information on the choice of priors and Markov chain Monte Carlo settings
☒	☐	For hierarchical and complex designs, identification of the appropriate level for tests and full reporting of outcomes
☐	☒	Estimates of effect sizes (e.g. Cohen's *d*, Pearson's *r*), indicating how they were calculated

Our web collection on statistics for biologists contains articles on many of the points above.

Software and code

Policy information about availability of computer code

Data collection	Reported in the Methods section of the manuscript. NGS data presented in the manuscript will behave been made publicly available through the Gene Expression Omnibus (GEO) database. The GEO IDs are: GSE222843 “Transcriptomic profiling of an in vivo mouse model of immune checkpoint induced colitis” for the bulk RNA-seq data comparing untreated, FMT only, CPI only and FMT+CPI treated wildtype mice in Figure 3, GSE241664 “Transcriptomic profile comparison of an in vivo model of dual combination immune checkpoint colitis or monotherapy immune checkpoint colitis” for the mouse RNA-seq data comparing the effects of mono and combination therapies in Figure 4, and lastly, GSE222959 “Single cell transcriptomics reveals colonic lymphocyte remodelling and emergence of polyfunctional, cytolytic lymphocyte responses in CPI-induced colitis” for the single cell RNA-seq data comparing the CPI+FMT treated mice and untreated wildtype mice for Figures 5-8. The 16S data in Figure 2 has been made publicly available and reposited at the ENA at the EBI with the accession number PRJEB65719.
Data analysis	Described in the Methods and Results sections of the manuscript.

For manuscripts utilizing custom algorithms or software that are central to the research but not yet described in published literature, software must be made available to editors and reviewers. We strongly encourage code deposition in a community repository (e.g. GitHub). See the Nature Portfolio guidelines for submitting code & software for further information.

Data

Policy information about availability of data

All manuscripts must include a data availability statement. This statement should provide the following information, where applicable:

- Accession codes, unique identifiers, or web links for publicly available datasets
- A description of any restrictions on data availability
- For clinical datasets or third party data, please ensure that the statement adheres to our policy

NGS data presented in the manuscript will behave been made publicly available through the Gene Expression Omnibus (GEO) database. The GEO IDs are: GSE222843 "Transcriptomic profiling of an in vivo mouse model of immune checkpoint induced colitis" for the bulk RNA-seq data comparing untreated, FMT only, CPI only and FMT+CPI treated wildtype mice in Figure 3, GSE241664 "Transcriptomic profile comparison of an in vivo model of dual combination immune checkpoint colitis or monotherapy immune checkpoint colitis" for the mouse RNA-seq data comparing the effects of mono and combination therapies in Figure 4, and lastly, GSE222959 "Single cell transcriptomics reveals colonic lymphocyte remodelling and emergence of polyfunctional, cytolytic lymphocyte responses in CPI-induced colitis" for the single cell RNA-seq data comparing the CPI+FMT treated mice and untreated wildtype mice for Figures 5-8. The 16S data in Figure 2 has been made publicly available and reposited at the ENA at the EBI with the accession number PRJEB65719.

Research involving human participants, their data, or biological material

Policy information about studies with human participants or human data. See also policy information about sex, gender (identity/presentation), and sexual orientation and race, ethnicity and racism.

Reporting on sex and gender	N/A
Reporting on race, ethnicity, or other socially relevant groupings	N/A
Population characteristics	N/A
Recruitment	N/A
Ethics oversight	N/A

Note that full information on the approval of the study protocol must also be provided in the manuscript.

Field-specific reporting

Please select the one below that is the best fit for your research. If you are not sure, read the appropriate sections before making your selection.

☒ Life sciences ☐ Behavioural & social sciences ☐ Ecological, evolutionary & environmental sciences

For a reference copy of the document with all sections, see nature.com/documents/nr-reporting-summary-flat.pdf

Life sciences study design

All studies must disclose on these points even when the disclosure is negative.

Sample size	Power calculations & statistical methods: Power calculations are informed by relevant in house experiments using our preclinic CPI-colitis model. For example one of our hypotheses is that cytokines, like IL23, are important amplifiers of IL27-primed CXCR6+ CD4 T-cells, and are functionally relevant in CPI-colitis. Administration of anti-IL23p19 antibodies (or control mAbs) significantly impacted key disease readouts as seen in Figure 8. Although IL23 neutralization did not completely "cure" CPI-colitis, it did significantly reduce disease features, including >20% reduction in colon mass, >65% reduction in colonic neutrophils and >60% reduction in the proportion of mucosal CD4+ T-cells co-producing interferon-γ and TNF. Based on the effect size observed in the most conservatively reduced phenotypic change (colon mass, M2-M1=64mg), and the variation observed (SD=32), we would require n=7 mice in each group to observe a significant (0.05) difference with 90% power. Validation of experimental observations will be achieved by performing at least 2 independent replicates, therefore, for each experiment proposed, n=14 mice will be required overall for each experimental evaluation. Although some interventions, such as IL27 neutralization/ablation may have a more profound impact on disease, for added confidence, we have still modelled sample size calculations based on the same modest reductions in outcomes as observed following IL23 blockade. Statistical analyses between 2 groups will include t-tests and ANOVA (parametric data) and Mann-Whitney U-tests and Kruskal-Wallis tests (non-parametric data).
Data exclusions	No data has been excluded

Replication	Experiments were repeated at least twice and performed independently. All attempts shown were successful and included in the Source Data
Randomization	Mice were randomised in the cage and to ensure no cage variation etc CPI+FMT were mixed in the same cage as CPI only or FMT only or PD-1 only was mixed with CTLA-4 only and combo, as examples. Only mice that were kept separate were control untreated mice due to the FMT and not wanting to alter the control mice microbiome within the cage environment. RNA samples were randomly chosen out of the main cohort of experiments as long as they passed QC.
Blinding	Investigators (bioinformaticians, histologists etc) were blinded to the condition of each mouse by being given generic numbers for the mouse sample e.g. listing the mice as WT1-16 instead of by condition when they were analysing the histology scoring, transcriptomics etc.

Reporting for specific materials, systems and methods

We require information from authors about some types of materials, experimental systems and methods used in many studies. Here, indicate whether each material, system or method listed is relevant to your study. If you are not sure if a list item applies to your research, read the appropriate section before selecting a response.

Materials & experimental systems

n/a	Involved in the study
☐	☒ Antibodies
☒	☐ Eukaryotic cell lines
☒	☐ Palaeontology and archaeology
☐	☒ Animals and other organisms
☒	☐ Clinical data
☒	☐ Dual use research of concern
☒	☐ Plants

Methods

n/a	Involved in the study
☒	☐ ChIP-seq
☐	☒ Flow cytometry
☒	☐ MRI-based neuroimaging

Antibodies

Antibodies used	Mice treated with immune checkpoint blockade drugs were intraperitoneally administered anti-CTLA-4 (9H10, BioXCell), using doses of 200µg, and anti-PD-1, (RMP1-14, BioXCell) at a dose of 250µg, once per week 66. Mice treated with depleting antibodies were intraperitoneally administered once a week, at the same time of giving anti-CTLA-4 and anti-PD-1 antibodies, either 500µg anti-TNFα (XT3.11, BioXCell), 500µg anti-IFNγ (H22, BioXCell) or 150µg anti-IL-23(p19) (G23-8, BioXCell). Control-isotype clones used were 2A3 (rat IgG2a) and HRPN (rat IgG1) at the same dose as the drug required
Validation	Antibodies were used from experience on previous experiments, (Wei SC, et al. Distinct Cellular Mechanisms Underlie Anti-CTLA-4 and Anti-PD-1 Checkpoint Blockade. Cell 170, 1120-1133.e1117 (2017), Powell N, et al. The transcription factor T-bet regulates intestinal inflammation mediated by interleukin-7 receptor+ innate lymphoid cells. Immunity 37, 674-684 (2012)) and published data by the company website (A D Christensen, Depletion of regulatory T cells in a hapten-induced inflammation model results in prolonged and increased inflammation driven by T cells Clin Exp Immunol. 2015 Mar; 179(3): 485–499.) and Ashley L. Steed, Gamma Interferon Blocks Gammaherpesvirus Reactivation from LatencyJ Virol. 2006 Jan; 80(1): 192–200. 10.1128/JVI.80.1.192-200.2006)

Animals and other research organisms

Policy information about studies involving animals; ARRIVE guidelines recommended for reporting animal research, and Sex and Gender in Research

Laboratory animals	Mouse strains (Balb/C wildtype, C56BL/6 wildtype and Il-23ko mice) used have been listed in the methods. The age at the beginning of experiments was typically around 6-7 weeks old. Sex of mice was kept the same in the data shown, for reproducibility purposes and without biased towards weights of organs measured etc. All mice were housed in accordance with the ASPA Home Office regulations at either Imperial College London or King's College London
Wild animals	This study did not involve wild mice
Reporting on sex	No sex biased was used here
Field-collected samples	This study does not include any field collected samples
Ethics oversight	The ethics and Home Office licence used were approved here at Imperial College London (Imperial AWERB team) and King's College London (AWERB team) under the licences approved by the Home Office for Home Office Licence Numbers PPL: 70/6792, 70/8127, 70/7869, P8999BD42

Note that full information on the approval of the study protocol must also be provided in the manuscript.

Flow Cytometry

Plots

Confirm that:

☒ The axis labels state the marker and fluorochrome used (e.g. CD4-FITC).

☒ The axis scales are clearly visible. Include numbers along axes only for bottom left plot of group (a 'group' is an analysis of identical markers).

☒ All plots are contour plots with outliers or pseudocolor plots.

☒ A numerical value for number of cells or percentage (with statistics) is provided.

Methodology

Sample preparation	Mouse colons were excised and placed in cold Phosphate Buffered Saline (PBS) solution. The tissue was digested in HBSS with 2% FCS and supplemented with 0.5mg/ml collagenase D, 10µg/ml DNase I and 1.5mg/ml dispase II (all Roche). The digested lymphocyte-enriched population was harvested using a 40%-80% Percoll gradient centrifugation for cLP.<br><br>Single suspension extracted cells, as described above, were plated into flow cytometry tubes (Sarstedt) at a concentration of 1 x 10^6 per ml. Cells were stimulated with 50ng/ml phorbol 12-myristate 13-acetate (PMA), 1µg/ml ionomycin, 2µM monensin (all Sigma Aldrich) for 3-4 hours. FcR receptor blocking antibodies were added before staining with antibodies. Surface staining antibodies were added with live/dead stain (Invitrogen). For intracellular staining, cells were fixed and permeabilised using the Foxp3 fixation/permeabilization buffer kit (Thermo Fisher) according to the manufacturer's instructions.
Instrument	Samples were acquired using a BD LSRFortessa (BD Biosciences).
Software	Sample data was recorded in FCS 3.0 data format using BD FACSDiva 6.0 software (BD Biosciences). Analysis of the data was performed using FlowJo software (Treestar Inc., Ashland, OR, USA).
Cell population abundance	Cell populations were sorted using a BD Aria and checked for <95% purity. Cells were measured with an automated cell counter to judge cell count and viability.
Gating strategy	Gating strategies are included in the Supplementary Figures

☒ Tick this box to confirm that a figure exemplifying the gating strategy is provided in the Supplementary Information.
